# The Ancestor and Evolution of the Giant Muscle Protein Connectin/Titin

**DOI:** 10.1007/s00239-025-10247-7

**Published:** 2025-04-27

**Authors:** Akira Hanashima, Yuu Usui, Ken Hashimoto, Satoshi Mohri

**Affiliations:** First Department of Physiology, Kawasaki Medical School, Kurashiki, 701-0192 Japan

**Keywords:** Connectin, Titin, Connectitin, Muscle evolution, Cnidarian, Ohnolog, Locomotion-vision theory

## Abstract

The emergence of connectin, also called titin, a muscular spring and the largest protein in living organisms, is critical in metazoan evolution as it enables striated muscle-based locomotion. However, its evolutionary history remains unclear. Here, we investigated the evolutionary process using genomic analysis and deduced the ancestor of connectin. The region between the *HOX* and *WNT* clusters in the human genome, where the connectin gene (*CON* (*TTN*)) is located, was quadrupled by two rounds of whole-genome duplication (WGD) in the ancestor of jawed vertebrates. However, connectin ohnologs were deleted before the advent of jawed vertebrates, resulting in a single connectin gene. Additionally, one of the connectin ohnologs created by the third round of teleost WGD disappeared, while the other was duplicated on the same chromosome. We also discovered that the connectin and connectin family genes consistently underwent local duplication on the same chromosome, though the underlying mechanism remains unknown. Using synteny analysis, we identified *KALRN* and its ohnolog *TRIO* as putative ancestral paralogs of the connectin gene. *TRIO/KALRN* has a connected structure of *SESTD1*-*CCDC141*-*CON* (*TTN*), and its synteny is conserved in the *Drosophila* genome. Furthermore, we confirmed that this connected structure, termed ‘connectitin,’ (connected-connectin/titin) is conserved in cnidarians and placozoans. Molecular phylogenetic analysis revealed that it diverged from *TRIO/KALRN* prior to the emergence of these animals, suggesting that metazoan muscle may have a single origin. These findings enhance our understanding of the evolutionary processes of striated muscles in the animal kingdom.

**Supplementary Information:**

The online version contains supplementary material available at 10.1007/s00239-025-10247-7.

## Introduction

The acquisition of locomotion using muscles is thought to have driven animal evolution. Muscle contraction is performed by the molecular movements of actin and myosin filaments, while extension requires passive mechanisms, such as the complementary actions of the biceps and triceps muscles in the elbow joints of humans. Therefore, controlling extensibility is important for each muscle to respond to the mechanical environment. Connectin, also called titin, is the largest protein in living organisms. It connects the Z-line to the M-line of the sarcomere in vertebrate striated muscles, including heart and skeletal muscles, and functions as a molecular spring during muscle relaxation, controlling muscular extensibility (Maruyama et al. 1976; Maruyama 1976; Wang et al. 1979; Granzier and Labeit 2025). Accordingly, exploring the evolution of connectin can provide important insights into the evolution of locomotion and cardiac function in animals.

Connectin is the third most abundant protein in skeletal muscle sarcomeres, following actin and myosin, and it accounts for 12% of the protein components by weight (Liversage et al. 2001). Connectin includes the I-band region, comprising Ig domains and unique elastic sequences; the A-band region, containing repeats of the Ig and FN3 domains; and a kinase domain (Labeit and Kolmerer 1995). Despite muscles requiring diverse mechanical properties to accommodate various physiological ranges of joint motion, mammals possess only one connectin gene. Splicing of the domains of the I-band region produces many isoforms. Skeletal muscle expresses a variety of N2A isoforms, from the small fast-twitch type (3,200 kDa) to the largest slow-twitch soleus type (3,800 kDa). Additionally, the heart expresses the long fetal N2BA isoform and the short adult N2B isoform, and by changing the expression ratio, myocardial extensibility is regulated depending on the mechanical environment. The human connectin gene is located in the chromosome 2q24.3, is 283 kb long and consists of 363 exons, the largest number of exons (Bang et al. 2001). Its largest exon is 17,106 bp. When all exons are spliced together, it spans 114 kb and codes for 38,138 amino acids, theoretically corresponding to a molecular weight of 4.2 MDa.

Human genes, such as nebulins and tropomodulins, functioning as muscle structural proteins, typically have four ohnologs (Hanashima et al. 2009; Bao et al. 2012; Nishikawa et al. 2019), which arose in the two rounds of whole-genome duplication (2R-WGDs) by quadruplication of a single ancestral gene in the ancestor of jawed vertebrates (Ohno 1970; Putnam et al. 2008; Yu et al. 2024; Marlétaz et al. 2024). These retained ohnologs may play important roles in the development of complex biological functions and suppressing inherited diseases caused by mutations. For instance, mutations in the connectin gene can cause myopathy and cardiomyopathy (Linke. 2023).

Connectin is also present in amphioxus and ascidians (Ohtsuka et al. 2011; Hanashima et al. 2012). In addition, connectin-like proteins are present in non-chordate muscles. For example, arthropods such as crayfish and *Drosophila* contain I-connectin/D-titin and projectin, which have domain structures similar to the I- and A-band regions of chordate connectin, respectively (Fukuzawa et al. 2001; Machado and Andrew 2000; Daley et al. 1998). Furthermore, connectin family proteins such as obscurin, which connects the center of myosin filaments in muscle sarcomeres to the sarcoplasmic reticulum, and MYLK, a kinase that phosphorylates myosin regulatory light chains, are widely present in bilaterians. Previous studies have suggested that the connectin ancestor became the largest protein by duplicating the Ig and FN3 domains many times, from which smaller family proteins such as MYLKs were derived (Higgins et al. 1994; Kenny et al. 1999). However, the origin and evolution of connectin remain unclear.

In this study, we investigated the process by which the connectin gene became singular after 2R-WGDs in the ancestor of jawed vertebrates. We also identified the ancestors and emergence of connectin. The acquisition of connectin by a common ancestor of cnidarians and placozoans likely played a significant role in enhancing muscular locomotion, thereby playing a pivotal role in the evolution of the animal kingdom in subsequent eras.

## Materials and Methods

### Synteny Analysis and Gene Search

Gene synteny was visually examined based on information from the following sources in the NCBI Gene database: Human (*Homo sapiens*, annotation RS_2023_10), Chicken (*Gallus gallus*, annotation 106), Xenopus (*Xenopus tropicalis*, annotation 104), Reedfish (*Erpetoichthys calabaricus*, annotation RS_2023_02), Spotted gar (*Lepisosteus oculatus*, annotation 101), Europian eel (*Anguilla anguilla*, annotation 100), Zebrafish (*Danio rerio*, annotation 106), Fugu (*Takifugu flavidus*, annotation RS_2023_06), Elephant shark (*Callorhinchus milii*, annotation 101), Ascidian (*Ciona intestinalis*, annotation 104), Amphioxus (*Branchiostoma floridae*, annotation 100), and Fly (*Drosophila melanogaster*, annotation Release 6.54). Ohnolog identification was performed using the OHNOLOG v2 database (Singh and Isambert. 2020). Genes encoding connectitin and trio/kalirin in non-bilaterians were identified using the NCBI database, including the Nucleotide collection (nr/nt) and Transcriptome Shotgun Assembly (TSA). A web-based BLAST search program (https://blast.ncbi.nlm.nih.gov/Blast.cgi) with the tblastn algorithm and default parameters was utilized. The domain structures of these proteins were identified using SMART (https://smart.embl.de; Letunic et al. 2021).

### Phylogenic Analysis

Amino acid sequence alignments using the MUSCLE program and phylogenetic tree construction using the Maximum Likelihood method were performed in MEGA 11 software version 11.0.13 (Tamura et al. 2021). Initial trees for the heuristic search were generated automatically using Neighbor-Join and BioNJ algorithms applied to a matrix of pairwise distances estimated using the JTT model. Bootstrapped analysis with 100 replicates were computed. Alignments generated using MEGA’s MUSCLE program were colored using BioEdit 7.7 (bioedit.software.informer.com) and visualized in Word 2016. Additionally, a sequence identity matrix illustrating alignment homology was created using BioEdit and presented in Excel 2016.

## Results

### Connectin Gene was Reduced to Single Copy in Human

To explore the process leading to the human connectin gene becoming singular, we examined the synteny surrounding the human connectin gene (chromosome 2q31), focusing on the *HOX* and *WNT* gene clusters, which are indicative of 2R-WGDs in vertebrates (Supplementary Fig. S1). In cephalochordate amphioxus and urochordate ascidian, which are closely related to vertebrates, the connectin and *WNT* genes are located on the same chromosome. This suggests that in the ancestors of chordates, the connectin gene was already co-located with the *WNT* gene cluster. Subsequently, following divergence from cephalochordates and urochordates, the *Hox* gene cluster likely aligned with the connectin gene and the *WNT* gene cluster on the same chromosome in vertebrate ancestors, which experienced a quadruplication event during 2R-WGDs.

**Fig. 1:**
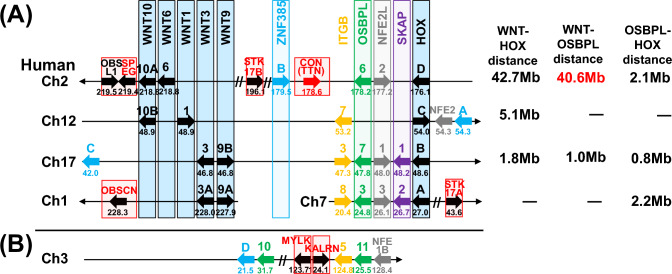
Synteny and ohnologs in the human genome. **A** Synteny between the *WNT-HOX* clusters and the connectin gene. The ohnologs are highlighted in boxes of their respective colors. **B** Synteny near *KALRN*. The connectin gene was reduced to one copy due to the deletion of a genomic region duplicated by the 2R-WGDs. However, the ancestral gene was originally present in the human genome

We investigated gene synteny between and near the *WNT* and *HOX* gene clusters on human chromosomes (Fig. 1A). One paralogon member lost synteny due to translocation, resulting in *WNT* on chromosome 1 and *HOXA* on chromosome 7. In contrast, chromosome 12 retains synteny, albeit with an inversion between *WNT* and *HOXC*. However, synteny between the *HOX* and *WNT* gene clusters was largely conserved, with paralogous genes *ITGB, OSBPL, NEF2L*, and *SKAP* located near the *HOX* gene clusters. The connectin gene is positioned 0.1 Mb from *OSBPL6* on chromosome 2, but no connectin ohnologs were found around *OSBPL7* and *OSBPL3* on their respective chromosomes. Furthermore, although the human connectin gene is located near *ZNF385B*, no ohnologs were found near *ZNF385A*, *ZNF385C*, or *ZNF385D*. The distances between *OSBPL6–HOXD* (2.1 Mb), *OSBPL3–HOXA* (2.2 Mb), and *OSBPL7–HOXB* (0.8 Mb) were relatively similar, whereas the distances between *WNT6*–*OSBPL6* (40.6 Mb) and *WNT9B*–*OSBPL7* (1.0 Mb) were substantially different. Therefore, it is likely that connectin ohnologs were lost due to large deletions between *WNT* and *OSBPL*s.

### Ohnologs of Connectin Family Created by 2R-WGDs were Reduced Before Jawed Vertebrates Diverged

**Fig. 2:**
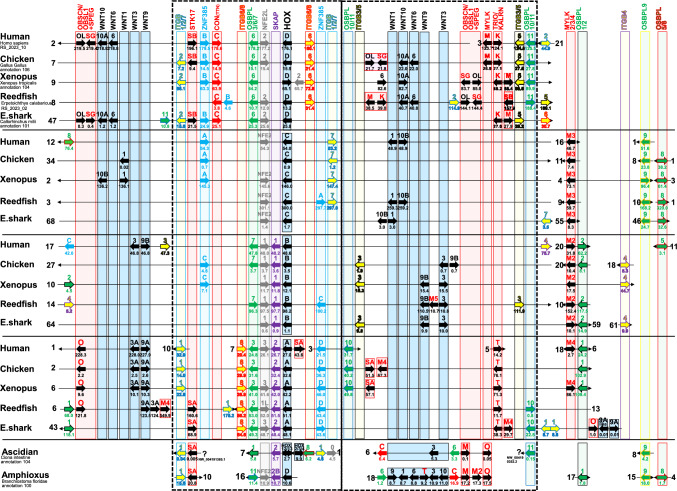
Synteny and ohnologs in jawed vertebrate genomes. Connectin genes are exclusively observed on the chromosome containing *HOXD*. The loss of connectin genes occurred prior to the divergence of gnathostomes. C, Connectin/titin. O, *OBSCN*. OL, *OBSL1*. SG, *SPEG*. SA, *STK17A*. SB, *STK17B*. M, *MYLK*. K, *KALRN*. T, *TRIO*. The ohnologs are highlighted in boxes of their respective colors. Dotted regions highlight syntenic similarities among chordates. For the elephant shark, the double letters (XX) in NCBI accession NW_0247047XX represent the chromosome number. Alignments of *WNT* ohnologs were referred to Konikoff et al. (2010), and the alignments of *ITGB* and *OSBPL* ohnologs are based on Fig. S4 and S5. A diagram illustrating genes moved by intrachromosomal rearrangements, reshuffled to emphasize similarities, is shown in Fig. S2

Furthermore, to examine the timing and scenario behind the reduction of connectin genes and their relationship with connectin family genes, we compared chromosomal synteny with *HOX* and *WNT* among various jawed vertebrates. These included mammals (human), birds/reptiles (chicken), amphibians (xenopus), bony fish (reedfish), and cartilaginous fish (elephant shark) —all of which underwent 2R-WGDs (Fig. 2). To further explore the process of gene shuffling following 2R-WGDs, we created diagrams illustrating the synteny of duplicated genes with moderate emphasis (Fig. S2-1) and with high emphasis (Fig. S2-2). The connectin gene is consistently located close to *OSBPL6* in all examined animals, but not near other *OSBPL* family genes. Moreover, although the connectin gene is also located near *ZNF385B*, no ohnologs were found near *ZNF385A*, *ZNF385C*, or *ZNF385D*. Additionally, no connectin ohnologs were identified in the OHNOLOG v2 database (http://ohnologs.curie.fr/). Therefore, jawed vertebrates, which underwent 2R-WGDs, initially possessed up to four connectin genes but ultimately retained only one connectin gene located on the HOXD-containing chromosome. There is now strong evidence in the literature, especially from the two hagfish genome articles (Yu et al. 2024; Marlétaz et al. 2024), that 1R generated the HoxA/B cluster and the HoxC/D cluster, whereupon 2R gave rise to A and B and to C and D (Fig. S1). Therefore, it is suggested that after 1R, the connectin gene disappeared from the *HOXA/B* cluster and was present only in the *HOXC/D* cluster. Following 2R, it disappeared from the *HOXC* cluster and was retained only in the *HOXD* cluster.

To study the evolution of connectin family genes, evolutionary trees for the *ITGB* and *OSBPL* families were constructed. These analyses included a broad range of species, including gnathostomes, agnathostomes, urochordates, and cephalochordates, with the aim of identify ohnologs (Fig. S3 and Fig. S4). The *ITGB* family is composed of four subfamilies: *1/2/7*, *3/5*, *6/8*, and *4*. Similarly, the *OSBPL* family consists of six subfamilies: *1/2*, *3/6/7*, *5/8*, *9*, *10/11*, and *OSBP1/2*. *ITGB6/8*, which maps to the connectin gene, is an ohnolog and is evolutionarily closer to *ITGB3/5*, which maps to the *KALRN*, and more distantly related to *ITGB4* and *ITGB1/2/7*. Among the *ITGB* subfamilies, *ITGB6/8* and *ITGB3/5* are particularly useful for discussing the evolution of connectin. On the other hand, *OSBPL3/6/7*, which maps to the connectin gene, is an ohnolog, and is evolutionarily closer to *OSBP1/2* and *OSBPL1/2*, while being more distantly related to *OSBPL5/8*, *OSBPL9*, and *OSBPL10/11*, which map to the *KALRN*. It is thought that the *OSBP* family, an intracellular lipid receptor, has evolved and increased in number more rapidly than the *ITGB* family, which is responsible for cell–cell adhesion.

Next, we constructed a molecular phylogenetic tree of the *MYLK* family, encompassing a wide range of bilaterian species to identify ohnologs of *MYLK* genes (Fig. S5). Vertebrate *MYLK* corresponds to ascidian *MYLK* (*Ci-MYLK*) and amphioxus *MYLK2* (*Bf-MYLK2*), while vertebrate *MYLK2/3/4* correspond to amphioxus MYLK (*Bf-MYLK*). Notably, *Bf-MYLK*, *Bf-MYLK2*, and *Ci-MYLK* are located on the same chromosome that includes connectin genes (Fig. 2, Fig. S2-1, and Fig. S2-2). Therefore, it is thought that *MYLK* and *MYLK2/3/4* formed a paralogon with *HOXA/B/C/D* in the last common ancestor of vertebrates. Since *MYLK* and *MYLK2* are adjacent on an amphioxus chromosome, it is likely they were also adjacent in the last common ancestor of vertebrates. This adjacency accounts for why *HOXD*-*MYLK* and *HOXA*-*MYLK4* are found in similar syntenic positions on their respective chromosomes even after the 2R-WGDs. *MYLK2* and *MYLK3* probably lost synteny with *HOXB* and *HOXC* after the 2R-WGDs.

*TRIO*/*KALRN*, *STK17A*/*B*, and *OBSCN*/*OBSL1* + *SPEG* are ohnologs. Based on the recent hagfish genome studies (Yu et al. 2024; Marlétaz et al. 2024), we can conclude that these genes were duplicated during the 1R-WGD, whereas the duplications that occurred during the 2R-WGD were subsequently lost. The chromosomes containing *HOXA* and *HOXD* retained these connectin family genes after the 2R-WGD. It is likely that an unknown process of natural selection maintains connectin family genes within these chromosomes. Notably, the chromosome containing *HOXD* includes *OBSL1* and *SPEG* (Fig. 2, Fig. S2-1, and Fig. S2-2), which had split before the emergence of gnathostomes but consistently remain adjacent to each other despite random gene orientation. This chromosome also contains the connectin gene, and *KALRN* is always found adjacent to *MYLK* in the opposite direction. On the other hand, the chromosome containing *HOXA* features *OBSCN* but not connectin, and *TRIO* is located on the same chromosome as *MYLK4*, though at a distance and with inconsistent gene orientation. Additionally, the chromosomes containing *HOXC* and *HOXB* do not possess any other connectin family genes. Consequently, *MYLK3* and *MYLK2* are likely to have been separated from these chromosomes. Therefore, connectin family ohnologs created by 2R-WGDs were reduced before the divergence of jawed vertebrates.

### Two Connectin Genes Exist on the Same Chromosome in Teleosts that Experienced Another WGD

**Fig. 3:**
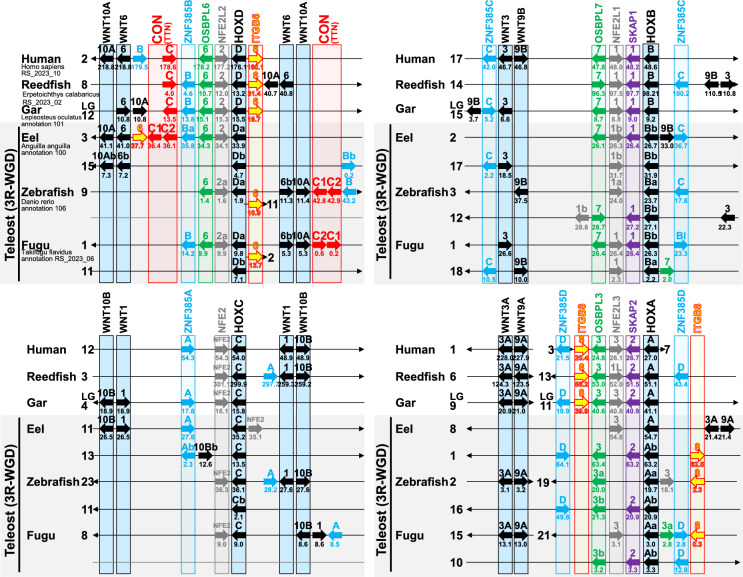
Synteny and ohnologs in teleost genomes. Two connectin genes exist on the same chromosome in teleosts. No connectin ohnolog generated by 3R-WGD was found. The ohnologs are highlighted in boxes of their respective colors. A diagram illustrating genes moved by intrachromosomal rearrangements, reshuffled to emphasize similarities, is shown in Fig. S6

We examined the region between the *WNT* and *HOX* gene clusters in teleost genomes (eel, zebrafish, and fugu), which experienced the third round of WGD (3R-WGD), and compared them with non-teleost genomes (human, reedfish, and gar) that did not undergo 3R-WGD (Fig. 3 and Fig. S6). *HOXA-D* in teleosts that underwent 3R-WGD were located on two chromosomes, except for the cases of zebrafish *HOXD* and fugu *HOXC*. However, the two connectin genes in teleosts were located close to each other on only one of the two chromosomes containing *HOXD*. Therefore, it is likely that one of the connectin ohnolog genes duplicated in 3R-WGD either moved next to the other ohnolog or one of them disappeared and was duplicated on the same chromosome as a paralog of the non-ohnolog before teleost diversification. The latter scenario is more plausible because one of the two chromosomes containing *HOXD* lost not only the connectin gene but also the adjacent genes.

### Connectin Family Genes Might have Been Duplicated on the Same Chromosome

**Fig. 4:**
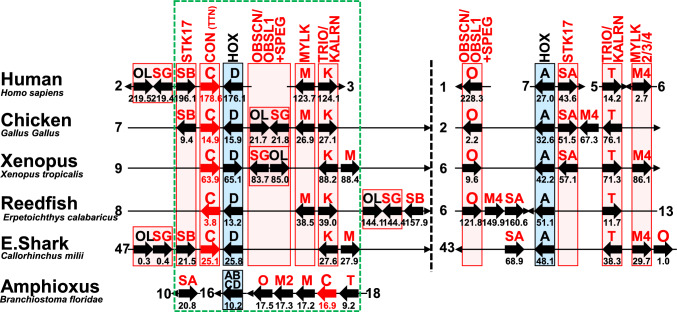
Connectin family genes on chordate chromosomes. All connectin family genes were located on one ancestral chromosome in the last common ancestor of vertebrates. This suggests that the connectin family genes may have originated through the duplication on one ancestral chromosome. O, *OBSCN*. OL, *OBSL1*. SG, *SPEG*. SA, *STK17A*. SB, *STK17B*. M, *MYLK*. K, *KALRN*. T, *TRIO*. In the case of the elephant shark, XX in NCBI accession NW_0247047XX represents the chromosome number. Dotted regions highlight syntenic similarities among chordates

We subsequently focused on the connectin family genes (Fig. 4). Each paralog of the connectin family genes, including *CON* (*TTN*), *OBSCN/OBSL1* + *SPEG*, *MYLK*, *TRIO/KALRN*, and *STK17*, which contain connectin-like kinases, exists on chromosomes containing *HOX* gene clusters. Interestingly, one ohnolog is always present on chromosomes containing *HOXD* in vertebrates. Amphioxus chromosome 18 also contains connectin family genes such as *CON* (*TTN*), *OBSCN*, *MYLK*, *MYLK2*, and *TRIO*. These findings suggest that the common ancestor of connectin family genes duplicated on the same chromosome to create each family gene before the emergence of chordates. This duplication mechanism resembles the duplication of connectin gene in teleosts, which occurred on the same chromosome rather than through WGD as ohnologs.

### Connectitin (Connected Connectin/Titin) Diverged from *TRIO/KALRN* Before Bilaterian Emergence

In humans, the ohnologs of gene families adjacent to connectin, such as *ITGB*, *OSBPL*, and *NFE2L*, are highly conserved in synteny (Fig. 1A). This conservation led us to hypothesize that searching these gene family sequences might reveal novel syntenic regions, potentially identifying a candidate for the ancestral connectin gene that existed before the 2R-WGDs. Fortunately, we found that *KALRN* is located just 0.9 Mb from *OSBPL11* on human chromosome 3q21 (Fig. 1B). *ITGB5* and *NFE1B/GATA2* are also located near *KALRN*.

**Fig. 5:**
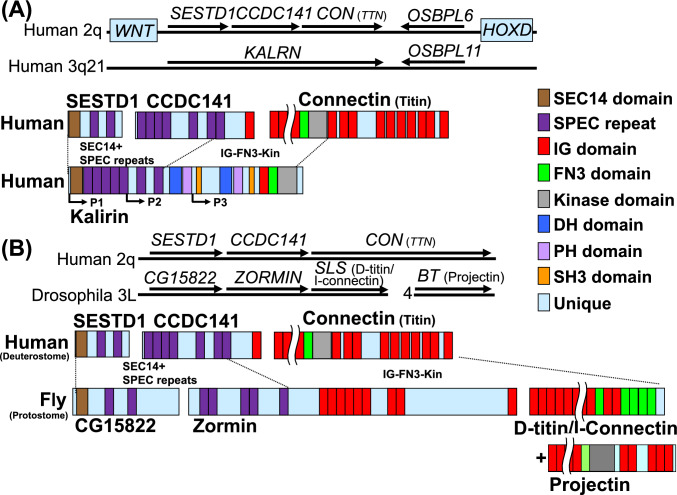
Connectitin and *KALRN*. **A** Domain structure commonality between human connectitin proteins (SESTD1, CCDC141, and connectin/titin) and kalirin. Isoforms are generated from internal promoter 1–3 (P1-3). Wavy lines denote omitted regions. **B** Domain structure commonality of connectitin proteins in human (SESTD1, CCDC141, connectin/titin) and *Drosophila* (CG15822, zormin, and I-connectin/D-titin). Drosophila features I-connectin/D-titin, which functions as a molecular spring like the connectin I-band region, and projectin, which binds to myosin filaments similar to the connectin A-band region. The domain structure of connectitin proteins resembles that of kalirin, and synteny in connectitin was established before the divergence of protostomes and deuterostomes

Kalirin regulates cytoskeletal dynamics in the nervous system. The C-terminus of kalirin contains an Ig-FN3-kinase domain homologous to that found in connectin (Fig. 5A). The central region of kalirin contains the DH*,* PH, and SH3 domains, similar to obscurin, another member of the connectin family. The N-terminus of kalirin contains an SEC14 domain and nine SPEC repeats. Therefore, we searched the 5’ upstream region of the human connectin gene and found *SESTD1* (with an SEC14 domain and three SPEC repeats) and *CCDC141* (with six SPEC repeats and an Ig domain) (Fig. 5A). *KALRN* is similar to the connectin gene in that it is splicing-regulated by *RBM20* (Guo et al. 2012), and its three internal promoters appear to correspond to the transcription start positions of *SESTD1*, *CCDC141*, and connectin genes.

**Fig. 6:**
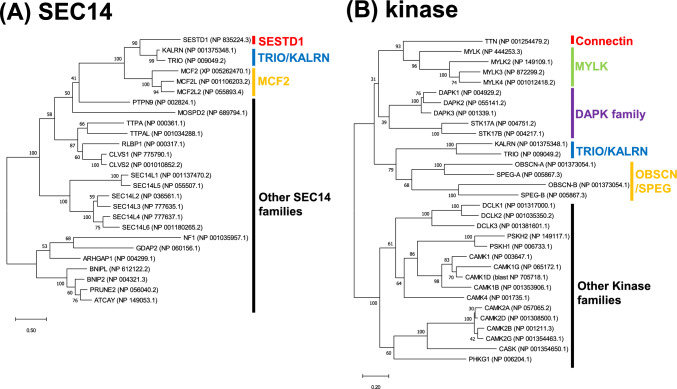
Molecular phylogenetic analysis of connectitin and *TRIO/KALRN* in human. **A** SEC14 domain. **B** Kinase domain. DDBJ/EMBL/GenBank accessions are listed after each gene name. Numbers at tree nodes indicate bootstrap support based on 100 replicates. Connectitin (*SESTD1*-*CCDC141*-*CON(TTN)*) and *TRIO/KALRN* appear in close proximity on the phylogenetic trees

Molecular phylogenetic analysis of human genes further revealed that in the phylogenetic tree of the SEC14 domain, *KALRN* and its ohnolog *TRIO*, which is more ubiquitously expressed and involved in cell migration by regulating actin cytoskeleton remodeling, were most closely located to *SESTD1* (Fig. 6A). In the phylogenetic tree of the kinase domain, *TRIO*/*KALRN* was most closely located to *OBSCN* /*SPEG*, the *DAPK* family (*DAPK* and *STK17*, which mediate programmed cell death)*,* the *MYLK* family, and the connectin gene (Fig. 6B). Therefore, *TRIO*/*KALRN* is considered an ancestral paralog of the *SESTD1*-*CCDC141*-*CON* (*TTN*), named connectitin (*SESTD1* and *CCDC141*-connected connectin/titin). Due to the conservation of synteny, connectitin is thought to have originated from the duplication of a genomic region containing *KALRN* and its surrounding genes.

To determine when connectitin diverged from *TRIO*/*KALRN*, we investigated the synteny in Drosophila, a protostome. We found that the 5’-upstream region of *SLS* (D-titin), a connectin ortholog, contains *CG15822* with a SEC14 domain and two SPEC repeats, and *ZORMIN* with four SPEC repeats and several Ig domains. These genes could be considered orthologs of *SESTD1* and *CCDC141*, respectively (Fig. 5B). The syntenic conservation of these genes suggests that connectitin diverged from *TRIO*/*KALRN* before the separation of protostomes and deuterostomes, i.e., before the emergence of bilaterians.

### Cnidarians and Placozoans have Connectitin with Similar Domain Structure to Trio/Kalirin

**Fig. 7:**
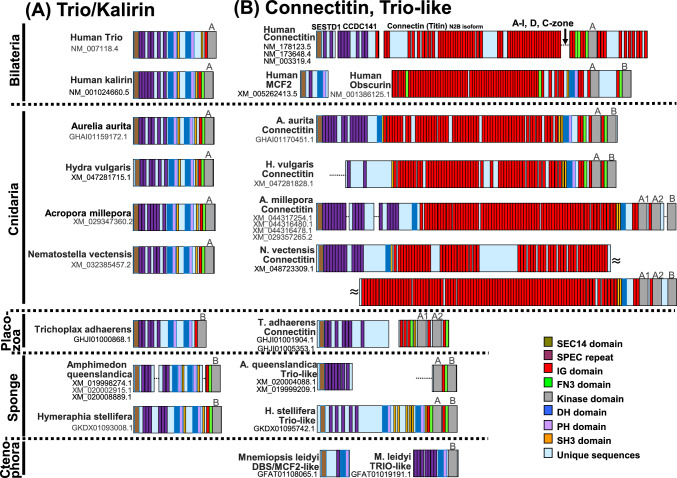
Connectitin and trio/kalirin in non-bilaterians. **A** Domain structures of trio/kalirin. **B** Domain structures of connectin-like protein. The domain structure of MCF2, which is closely related to trio/kalirin and connectitin, is also included for reference. The wavy lines in the Nematostella connectitin indicate that the sequence continues on the next line. Non-bilaterians, including cnidarian, placozoan, and sponges contain connectitin or trio-like protein in addition to a trio/kalirin

Next, we identified connectitin genes and *TRIO*/*KALRN* in non-bilaterians (Fig. 7). The cnidarian connectitin is similar to human connectitin and trio/kalirin, featuring a SEC14 domain and over 10 SPEC repeats at the N-terminus; numerous Ig domains; RhoGEF, PH, and SH3 domains in the central region; two kinase domains at the C-terminus in jellyfish and hydra; and three in coral and sea anemone. Placozoans also contain trio/kalirin and connectitin with two kinase domains. Sponges contain trio/kalirin and a trio-like protein with two kinase domains. In the ctenophore, the comb jelly *M. leidyi* contains a trio-like protein that is partially similar to trio/kalirin, with SPEC repeats, a DH domain, a PH domain, and a kinase domain.

### Connectitin Originated as a *TRIO*/*KALRN* Paralog Before the Emergence of Cnidarians and Placozoans and Diverged in Bilaterians

**Fig. 8:**
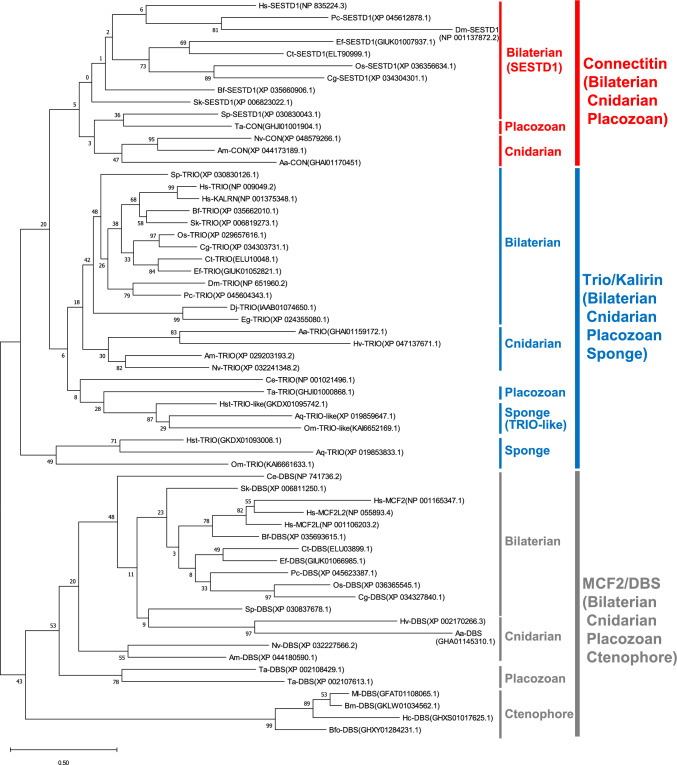
Molecular phylogenetic analysis of the SEC14 domain in connectitin and trio/kalirin. Numbers at tree nodes indicate bootstrap support based on 100 replicates. After *TRIO/KALRN* separated from *MCF2/DBS*, connectitin emerged as a *TRIO/KALRN* paralog before the emergence of cnidarians and placozoans and evolved into the connectin gene in bilaterians. In vertebrates, *KALRN* was copied from *TRIO* by WGD. Hs, Homo sapiens (human). Bf, Branchiostoma floridae (Amphioxus). Sk, Saccoglossus kowalevskii (Acorn worm). Sp, Strongylocentrotus purpuratus (Sea Urchin). Dm, Drosophila melanogaster (Fruit fly). Pc, Procambarus clarkia (Crayfish). Os, Octopus sinensis (Octopus). Cg, Crassostrea gigas (Oyster). Ef, Eisenia fetida (Earthworm). Ct, Capitella teleta (Ragworm). Ce, Caenorhabditis elegans (Roundworm). Eg, Echinococcus granulosus (Echinococcus). Dj, Dugesia japonica (Planaria). Aa, Aurelia aurita (Jellyfish). Hv, Hydra vulgaris (Hydra). Am, Acropora millepora (Coral). Nv, Nematostella vectensis (Sea anemone). Ta, Trichoplax adhaerens (Trichoplax). Aq, Amphimedon queenslandica (Demosponge). Hst, Hymeraphia stellifera (Demosponge). Om, Oopsacas minuta (Glass sponge). Ml, Mnemiopsis leidyi (comb jelly Lobata). Bfo, Beroe forskalii (comb jelly Beroe). Bm, Bolinopsis microptera (comb jelly Lobata). Hc, Hormiphora californensis (comb jelly Cydippida)

**Fig. 9:**
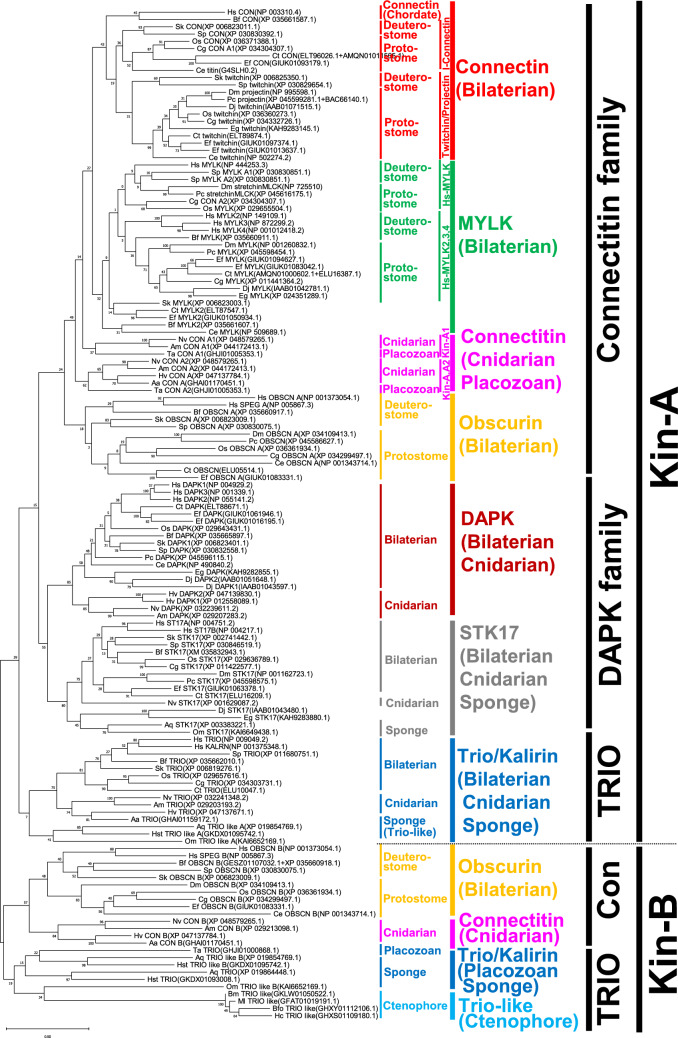
Molecular phylogenetic analysis of kinase domain in connectitin, DAPK, and trio/kalirin families. Numbers at tree nodes indicate bootstrap support based on 100 replicates. The NCBI/DDBJ/GenBank accession number is shown in parentheses. After *TRIO/KALRN* separated from *MCF2*, connectitin emerged as a *TRIO/KALRN* paralog before the emergence of cnidarians and placozoans and evolved into the connectin gene in bilaterians. Hs, Homo sapiens (human). Bf, Branchiostoma floridae (Amphioxus). Sk, Saccoglossus kowalevskii (Acorn worm). Sp, Strongylocentrotus purpuratus (Sea Urchin). Dm, Drosophila melanogaster (Fruit fly). Pc, Procambarus clarkia (Crayfish). Os, Octopus sinensis (Octopus). Cg, Crassostrea gigas (Oyster). Ef, Eisenia fetida (Earthworm). Ct, Capitella teleta (Ragworm). Ce, Caenorhabditis elegans (Roundworm). Eg, Echinococcus granulosus (Echinococcus). Dj, Dugesia japonica (Planaria). Aa, Aurelia aurita (Jellyfish). Hv, Hydra vulgaris (Hydra). Am, Acropora millepora (Coral). Nv, Nematostella vectensis (Sea anemone). Ta, Trichoplax adhaerens (Trichoplax). Aq, Amphimedon queenslandica (Demosponge). Hst, Hymeraphia stellifera (Demosponge). Om, Oopsacas minuta (Glass sponge). Ml, Mnemiopsis leidyi (comb jelly Lobata). Bfo, Beroe forskalii (comb jelly Beroe). Bm, Bolinopsis microptera (comb jelly Lobata). Hc, Hormiphora californensis (comb jelly Cydippida)

Finally, we examined the evolution of these genes using molecular phylogenetic analysis (Fig. 8, Fig. 9, Fig. S7, Fig. S8, and Supplementary file 1–4). In the phylogenetic tree of the SEC14 domain, connectitin in bilaterians, cnidarians, and placozoans, as well as trio/kalirin in bilaterians, cnidarians, and placozoans, and sponge trio-like proteins, were each classified into monophyletic groups (Fig. 8 and Fig. S7). Sponge trio/kalirin formed a sister group to these proteins, with metazoan MCF2-like proteins representing the ancestral group.

In the phylogenetic tree of the kinase domain, kinase-A (including A1 and A2, Fig. 7) of bilaterian connectin, bilaterian MYLK, bilaterian obscurin, and connectitin in cnidarians and placozoans were classified into a monophyletic group (Fig. 9 and Fig. S8). DAPK in bilaterians and cnidarians, along with STK17 in bilaterians, cnidarians, and sponges were placed in another monophyletic group. These sister groups were further categorized by kinase-A of trio/kalirin in bilaterian, cnidarian, and sponge trio-like proteins. Additionally, kinase-B of bilaterian obscurin and cnidarian connectitin formed a monophyletic group, with sister groups identified by kinase-B of trio and trio-like proteins in placozoans, sponges, and ctenophores. It is therefore suggested that connectitin originated as a *TRIO*/*KALRN* paralog before the emergence of cnidarians and placozoans, and subsequently diverged in bilaterians as connectin family genes.

## Discussion

### Retention and Loss of Connectin Family Genes after WGDs in Vertebrate

Recently, it has been reported that the *HOXA/B* and *HOXC/D* clusters were created in the 1R-WGD, with *HOXA* and *HOXB* generated from the *HOXA/B* cluster, and *HOXC* and *HOXD* from the *HOXC/D* cluster during the 2R-WGD (Yu et al. 2024; Marlétaz et al. 2024). Surprisingly, we found that chromosomes containing *HOXA* and *HOXD* duplicated during the 1R-WGD, showed greater similarity in the retention of connectin family orthologs compared to chromosomes containing *HOX* clusters duplicated during the 2R-WGD (Fig. 2). It is suggested that *CON* (*TTN*), *MYLK*, *TRIO*/*KALRN*, *OBSCN*/*OBSL1* + *SPEG*, and *STK17A*/*B* were present on the chromosome containing the *HOXA/B/C/D* cluster in the last common ancestor of vertebrates (Fig. 4). During the 1R-WGD, the chromosome containing the *HOXC/D* cluster retained all these ohnologs (*CON* (*TTN*), *MYLK*, *KALRN*, *OBSL1* + *SPEG*, *STK17B*), whereas the chromosome containing the *HOXA/B* cluster lost the ohnologs of *CON* (*TTN*) and *MYLK*, but retained *TRIO*, *OBSCN*, and *STK17A*. However, during the 2R-WGD, only the chromosomes containing *HOXA* and *HOXD* retained all the ohnologs, whereas those containing *HOXB* and *HOXC* retained none.

The translocation of *ITGB2* and *KALRN* from chromosomes containing *CON* (*TTN*)-*HOXD*, as observed in the human genome (Fig. 1, Fig. 2 and Fig. 3), likely occurred during the evolution from Primatomorpha to Primata (Damas et al. 2022). We confirmed that in non-Primata species of Primatomorpha, such as the Philippine flying lemur (*Cynocephalus volans*), connectin, *ITGB2*, and *KALRN* were present on the same chromosome. However, in Primata species, such as the gray mouse lemur (*Microcebus murinus*), they were located on separate chromosomes. This suggests that the chromosome containing *CON* (*TTN*)-*HOXD* was strongly constrained by the evolutionary pressure for synteny conservation during the evolutionary process up to Primatomorpha, which weakened at the K-Pg boundary, leading to translocation. It is intriguing to consider that the chromosomes containing *HOXD* in the common ancestor of primates might have been shattered by the impact of a giant meteorite. Elucidating the mechanism by which only one chromosome retains the connectin family ohnologs after WGD, and the constraint imposed by the evolutionary pressure for synteny conservation, is an important future challenge that will directly enhance our understanding of gene retention and loss.

### Connectin Ohnolog Absence in Vertebrates

We revealed that the connectin gene, which encodes a molecular spring in striated muscle, became a single gene after the 2R-WGD and before the divergence of jawed vertebrates (Fig. 2). Although it is not a precise reconstruction of the evolutionary process, it is speculated that, starting from the highly ordered state after 2R-WGDs (Fig. S2-2), entropy gradually increased (Fig. S2-1), leading to the current gene sequence (Fig. 2). Teleosts also lost the ohnolog amplified by 3R-WGD; however, the connectin gene duplicated on the same chromosome, resulting in two paralogs (Fig. 3 and Fig. S6). The connectin gene of zebrafish, *TTNA* (*TTN.2*) is essential for the assembly of cardiac sarcomeres and the subsequent establishment of cardiac contractility. Additionally, not only *TTNA* (*TTN.2*) but also *TTNB* (*TTN.1*) is required for sarcomere assembly in somites (Seeley et al. 2007). Having a single connectin gene may increase susceptibility to mutation-induced disease development compared to molecules with multiple genes, such as α-actinin genes (*ACTN1-4*). Moreover, the evolution of complex locomotion necessitates connectins with a variety of mechanical properties, making adaptation with a single gene seemingly disadvantageous. However, the human connectin gene contains two additional 10xIg super-repeats in the I-band elastic region, unlike the teleost connectin gene. These allow for the production of various isoforms through alternative splicing. These triplicate Ig regions are expressed in the human leg and diaphragm (Labeit and Kolmerer. 1995), suggesting that connectin evolution facilitated walking with limbs and lung respiration, which emerged in terrestrial life by enabling diverse muscle extension properties (Hanashima et al. 2017). Thus, it is clear that flexibility or adaptability can arise both by gene duplication and alternative splicing. During vertebrate evolution, the connectin gene has selected the latter, while in teleosts, it has favored the former.

### Connectin Evolution in Bilaterians

Similar to vertebrate connectins, urochordate and cephalochordate connectins possess an I-band region responsible for elasticity and an A-band region that binds to myosin filaments (Ohtsuka et al. 2011; Hanashima et al. 2012). In contrast, arthropods and Caenorhabditis elegans have proteins known as I-connectin/D-titin and Ce-titin, respectively, which correspond to the I-band of chordate connectins, and projectin/twitchin, which correspond to the A-band (Fukuzawa et al. 2001; Oshino et al. 2003; Benian et al. 1989; Flaherty et al. 2002). Therefore, there are three possible scenarios for the evolution of connectins in bilaterian animals: the division of a single gene into two in protostomes, the fusion of two separate genes in chordates, or the loss of one gene with a convergent gain of its function in chordates. This study demonstrated the commonality between human connectitin (*SESTD1*-*CCDC141*-*CON* (*TTN*)) and Drosophila connectitin (*CG15822*-*ZORMIN*-*SLS*) and suggested that I-connectin/D-titin (SLS protein) in arthropods corresponds to vertebrate connectin (Fig. 5). Molecular phylogenetic analysis also suggests that ancestral connectin separates into I-connectin/D-titin/Ce-titin and projectin/twitchin in non-chordates (Fig. 9). However, some phylogenetic trees indicated that the vertebrate connectin gene corresponds to projectin/twitchin, whereas the genes corresponding to I-connectin/D-titin/Ce-titin were absent. Therefore, it is not possible to conclusively determine which of these scenarios reflects the true series of evolutionary events. Future functional analyses of these proteins should clarify the evolutionary history of connectin in bilaterians.

### Bilaterian Connectitin

The conservation of the *SESTD1*-*CCDC141*-*CON* (*TTN*) across phyla in bilaterians (Fig. 5) suggests the existence of a common ancestor with a single gene that was duplicated from *TRIO*/*KALRN*, later enlarged, and passed on to extant animals. We named this hypothetical single gene possessed by ancient animals “connectitin” (connected connectin/titin). Although chordates and arthropods diverged more than 500 Mya, the human orthologs, *CCDC141* and *CON* (*TTN*), and the *Drosophila* orthologs, *ZORMIN* and *SLS,* have retained their adjacent relationships, as reported in transcripts spanning them (Labeit et al. 2006; Burkart et al. 2007). The functions of these three genes may be interrelated, similar to those of the *HOX* gene cluster, thereby preventing gene segregation. However, further investigation into the functions of *SESTD1* and *CCDC141* is required to elucidate the reasons for synteny conservation in connectitin.

### Connectin Family Evolution

The connectin family genes such as *CON/TTN*, *MYLK*, and *OBSCN*, *TRIO/KALRN*, and *STK17* are found on the same chromosome (Fig. 4), suggesting they may have originated as paralogs on the same chromosome, similar to the two connectin genes in teleosts (Fig. 3). However, pinpointing the exact order in which they appeared as paralogs is challenging. Molecular phylogenetic analysis indicates that *STK17* is also present in sponges (Fig. 9), suggesting that the common ancestor of the connectitin family and *DAPK* family diverged from *TRIO/KALRN* around the time sponge emerged. *MYLK*s are thought to have diverged from connectitin in the common ancestor of bilaterians, subsequently evolving into vertebrate *MYLK* (which functions in smooth muscle) and vertebrate *MYLK2-4* (involved in striated muscle) before bilaterian diversification (Fig. 9). In contrast, *OBSCN* is phylogenetically placed as a sister group to *MYLK*, bilaterian connectitin, and cnidarian connectitin, though it is absent in cnidarians (Fig. 9). *OBSCN* possesses two kinases (Fig. 7) and exhibits rapid amino acid sequence changes due to functional complementation, suggesting the possibility that *OBSCN* diverged from connectitin gene during bilaterian emergence. Additionally, *TRIO* originally contained only kinase-B, and before the appearance of sponges, kinase-A was duplicated, and then *TRIO*-like genes were duplicated (Fig. 7). Subsequently, sponge *TRIO*-like genes retained both kinases, whereas sponge and placozoan *TRIO* lost kinase-A, and cnidarian and bilaterian *TRIO* lost kinase-B. However, it remains unclear whether connectitin in cnidarians and bilaterians evolved directly from *TRIO*-like genes, diverged from *TRIO*, or diverged from a common ancestor of the *TRIO* and *TRIO*-like genes. Nevertheless, the evolution of the connectin family, especially the emergence of *OBSCN* and connectitin, requires further research that integrates various sources of information such as functional and molecular phylogenetic analyses.

### Connectitins of Cnidarians and Placozoans

In present study, we discovered for the first time that connectitin is present not only in bilaterians but also in cnidarians and placozoans (Fig. 7). We also identified *TRIO*/*KALRN* as the paralogous ancestor of the connectitin, indicating that its duplication occurred prior to the emergence of cnidarians and placozoans (Fig. 8, 9). This implies that cnidarian and bilaterian connectitin are monophyletic, contrasting with earlier findings that suggested an independent evolution of striated muscles in cnidarians and bilaterians (Steinmetz et al. 2012). This discrepancy arises because, in bilaterians, the components of striated and smooth muscles are not always distinctly different (Sulbaran et al. 2015), and their ancestors likely had both visceral smooth and somatic striated muscle cells, with smooth myocytes repeatedly adopting the striated contractile module (Brunet et al. 2016).

### The Muscle Origin and Evolution

The fact that connectin, crucial for striated muscles, is monophyletic across all the identified animal phyla suggests that striated muscles in extant animals may have a single origin. Furthermore, we revealed the evolutionary history of connectin, including the duplication of the ancestral gene *TRIO*/*KALRN* before the emergence of placozoans and cnidarians (Fig. 8 and Fig. 9), and its enlargement as a muscle molecular spring in cnidarians and later (Fig. 7). Since the evolution of muscle tissue is directly linked to the advancement in locomotion, its temporal identification is important when considering the evolution of multicellular organisms. However, the divergence time of cnidarians cannot be precisely determined at this point. Fossil materials indicate that they date back to the Ediacaran period (Dunn et al. 2022) at the latest, and molecular phylogenetic analysis suggests an even earlier Cryogenian period (Erwin et al. 2011).

On the other hand, skeletal muscles generate high tension by attaching to hard tissues: the endoskeleton of vertebrates and the exoskeleton of arthropods in extant animals. Therefore, hard tissues are important, in addition to muscles, when considering the evolution of locomotion. The sudden appearance of hard tissue fossils is the most famous event in the history of animal evolution known as the Cambrian Explosion, which occurred 543 million years ago. Parker attributed the sudden appearance of hard tissue in the Cambrian Explosion to the acquisition of vision, and proposed the light-switch theory that exoskeletal development was accelerated by the intensification of the predator–prey relationship due to the appearance of animals with advanced vision (Parker. 2003). In this fascinating theory, the question remained as to why vision was briefly acquired during the Cambrian Explosion when animals with photoreceptors have existed for much longer. A candidate answer to this question is the possibility of coevolution of locomotion and vision through the acquisition of hard tissue. The idea is that the acquisition of hard tissues by Cambrian animals, which Ediacaran animals did not have, improved locomotion, which in turn improved vision as a new selection pressure, and that the improved vision generated positive feedback that further improved locomotion: This is the locomotion-vision theory. Since connectins also appeared in the Precambrian, it is possible that the major muscle genes of extant animals were all present. If this is the case, changes in the energy environment, such as an increase in oxygen concentration (Williams et al. 2019) and an increase in phosphorus supply (Shimura et al. 2014), would trigger the acquisition of hard tissues. These changes in the global environment might have greatly improved locomotion and vision.

A limitation of this study is the unclear reason why jellyfish were the only cnidarians with striated muscles, even though all cnidarians shared connectitin. Additionally, the absence of connectitin in ctenophores contradicts earlier reports (Mackie et al. 1988) that documented the presence of striated muscles in the tentilla of Euplokamis, a type of ctenophores. However, it was recently reported that striations were not visible in confocal microscopy of phalloidin-stained muscle fibers in the tentilla of Euplokamis dunlapae (Norekian and Moroz. 2020), raising the possibility that ctenophores do not have striated muscles. To answer these questions regarding the origin and evolution of metazoan muscles, further research is necessary to elucidate the role of connectitin in cnidarians, decode the genome of ctenophores such as Euplokamis, and reconfirm the presence of striated muscles.

## Conclusion

In this study, we demonstrate that the connectin gene, which functions as a spring molecule in the striated muscles of extant animals, originated as a paralog of *TRIO*/*KALRN* before the emergence of cnidarians and placozoans. It evolved into connectitin in cnidarians, and further diversified into three genes (*SESTD1*, *CCDC141*, and *CON* (*TTN*)) in bilaterians. Additionally, connectin ohnologs created by 2R-WGDs were lost prior to the divergence of jawed vertebrates. One of the connectin ohnologs generated in the teleost 3R-WGD disappeared, while the other was duplicated on the same chromosome. We also showed that the duplication of connectitin from *TRIO*/*KALRN* and the formation of connectin family genes in bilaterians likely occurred via gene duplication on a single chromosome. These results offer new insights into the evolution of animal locomotion and cardiac blood pumping, both of which rely on striated muscles.

## Supplementary Information

Below is the link to the electronic supplementary material.

Supplementary file1 (DOCX 544 KB)

Supplementary file2 (DOCX 52 KB)

Supplementary file3 (DOCX 176 KB)

Supplementary file4 (XLSX 32 KB)

Supplementary file5 (XLSX 123 KB)

## Data Availability

All sequence data used in this study were obtained from the DDBJ/EMBL/GenBank databases.
