# Supplementary material for: The Ancestor and Evolution of the Giant Muscle Protein Connectin/Titin
Source: J Mol Evol. 2025 Apr 27;93(3):306–21. doi: 10.1007/s00239-025-10247-7 (PMC12198301; doi:10.1007/s00239-025-10247-7)
Supplement: Supplementary file 1 — Supplementary file1 (DOCX 544 KB) [file 239_2025_10247_MOESM1_ESM.docx]

**Fig. S1** **Connectin gene and *HOX-WNT* clusters in the human genome.** The connectin gene is hypothesized to quadruple along with *HOX-WNT* clusters by 2R-WGDs in the ancestor of jawed vertebrates, originating from amphioxus and ascidians. Recent research indicates that the *HOXA/B* and *HOXC/D* clusters were created during the 1R-WGD, with *HOXA* and *HOXB* deriving from the *HOXA/B* cluster, while *HOXC* and *HOXD* originated from the *HOXC/D* cluster during the 2R-WGD (Yu et al. 2024; Marlétaz et al. 2024). Despite these events, only one connectin gene is present in the human genome.

**Fig. S2-1** **Moderately reshuffled version of synteny and ohnologs of the jawed vertebrate genomes shown in Fig. 2.** Genes moved by intrachromosomal rearrangements were reshuffled in the same order across species to highlight similarities. The ohnologs are highlighted in boxes of their respective colors. Dotted regions highlight syntenic similarities among chordates. Dark grey boxes represent reshuffled genes. C, Connectin/titin gene. O, *OBSCN*. OL, *OBSL1*. SG, *SPEG*. SA, *STK17A*. SB, *STK17B*. M, *MYLK*. K, *KALRN*. T, *TRIO*. For the elephant shark, the double letters (XX) in NCBI accession NW_0247047XX represents the chromosome number. Alignments of WNT ohnologs were referred to Konikoff et al. (2010), and the alignments of *ITGB* and *OSBPL* ohnologs were based on Fig. S3 and S4. Connectin genes are exclusively observed on the chromosome containing *HOXD*. The loss of connectin genes occurred prior to the divergence of gnathostomes.

**Fig. S2-2** **Highly reshuffled version of synteny and ohnologs of the jawed vertebrate genomes shown in Fig. 2.** Genes moved by intrachromosomal rearrangements were reshuffled in the same order across species to highlight similarities. The ohnologs are highlighted in boxes of their respective colors. Dotted regions highlight syntenic similarities among chordates. Dark grey boxes represent reshuffled genes. C, Connectin/titin gene. O, *OBSCN*. OL, *OBSL1*. SG, *SPEG*. SA, *STK17A*. SB, *STK17B*. M, *MYLK*. K, *KALRN*. T, *TRIO*. For the elephant shark, the double letters (XX) in NCBI accession NW_0247047XX represents the chromosome number. Alignments of WNT ohnologs were referred to Konikoff et al. (2010), and the alignments of *ITGB* and *OSBPL* ohnologs were based on Fig. S3 and S4. Connectin genes are exclusively observed on the chromosome containing *HOXD*. The loss of connectin genes occurred prior to the divergence of gnathostomes.

**Fig. S3** **Molecular phylogenetic tree of the *ITGB* family.** *ITGB6/8*, which maps to the connectin gene, is an ohnolog and evolutionarily closer to *ITGB3/5*, which maps to the *KALRN*, and more distantly related to *ITGB4* and *ITGB1/2/7*. Among the *ITGB*s, *ITGB6/8* and *ITGB3/5* are considered useful for discussing the evolution of connectin. Hs, Homo sapiens (human). Xt, Xenopus tropicalis (Xenopus). Cm, Callorhinchus milii (Elephant shark). Pm, Petromyzon marinus (Lamprey). Mg, Myxine glutinosa (Hagfish). Ci, Ciona intestine (Ascidian). Bf, Branchiostoma floridae (amphioxus).

**Fig. S4** **Molecular phylogenetic tree of the OSBP family.** *OSBPL3/6/7*, which maps to the connectin gene, is an ohnolog and is evolutionarily closer to *OSBP1/2* and *OSBPL1/2*. It is more distantly related to *OSBPL5/8*, *OSBPL9*, and *OSBPL10/11*, which map to the *KALRN*. Hs, Homo sapiens (human). Xt, Xenopus tropicalis (Xenopus). Cm, Callorhinchus milii (Elephant shark). Pm, Petromyzon marinus (Lamprey). Mg, Myxine glutinosa (Hagfish). Ci, Ciona intestine (Ascidian). Bf, Branchiostoma floridae (amphioxus).

**Fig. S5 Molecular phylogenetic tree of the bilaterian *MYLK* family.** The kinase domain of human connectin/titin is included as an outgroup. Vertebrate *MYLK* forms a distinct subgroup that diverged from vertebrate *MYLK2/3/4* early in bilaterian evolution. Vertebrate *MYLK* corresponds to *Ci-MYLK* and *Bf-MYLK2*, while vertebrate *MYLK2/3/4* are ohnologs and correspond to *Bf-MYLK*. Hs, Homo sapiens (human). Ci, Ciona intestine (Ascidian). Bf, Branchiostoma floridae (Amphioxus). Sk, Saccoglossus kowalevskii (Acorn worm). Sp, Strongylocentrotus purpuratus (Sea Urchin). Dm, Drosophila melanogaster (Fruit fly). Pc, Procambarus clarkia (Crayfish). Os, Octopus sinensis (Octopus). Cg, Crassostrea gigas (Oyster). Ef, Eisenia fetida (Earthworm). Ct, Capitella teleta (Ragworm). Ce, Caenorhabditis elegans (Roundworm). Eg, Echinococcus granulosus (Echinococcus). Dj, Dugesia japonica (Planaria).

**Fig. S6** **Reshuffled version of synteny and ohnologs in the teleost genomes (shown in Fig. 3).** Genes moved by intrachromosomal rearrangements were reshuffled in the same order across species to highlight similarities. The ohnologs are highlighted in boxes of their respective colors. Dark grey boxes represent reshuffled genes. Two connectin genes exist on the same chromosome in teleosts. No connectin ohnologs generated by the 3R-WGD were found.

**Fig. S7 Collapsed molecular phylogenetic tree of the SEC14 domain in connectitin and trio/kalirin.** All bifurcations of Fig.8 below cutoff 30 were collasped. Hs, Homo sapiens (human). Bf, Branchiostoma floridae (Amphioxus). Sk, Saccoglossus kowalevskii (Acorn worm). Sp, Strongylocentrotus purpuratus (Sea Urchin). Dm, Drosophila melanogaster (Fruit fly). Pc, Procambarus clarkia (Crayfish). Os, Octopus sinensis (Octopus). Cg, Crassostrea gigas (Oyster). Ef, Eisenia fetida (Earthworm). Ct, Capitella teleta (Ragworm). Ce, Caenorhabditis elegans (Roundworm). Eg, Echinococcus granulosus (Echinococcus). Dj, Dugesia japonica (Planaria). Aa, Aurelia aurita (Jellyfish). Hv, Hydra vulgaris (Hydra). Am, Acropora millepora (Coral). Nv, Nematostella vectensis (Sea anemone). Ta, Trichoplax adhaerens (Trichoplax). Aq, Amphimedon queenslandica (Demosponge). Hst, Hymeraphia stellifera (Demosponge). Om, Oopsacas minuta (Glass sponge). Ml, Mnemiopsis leidyi (comb jelly Lobata). Bfo, Beroe forskalii (comb jelly Beroe). Bm, Bolinopsis microptera (comb jelly Lobata). Hc, Hormiphora californensis (comb jelly Cydippida).

**Fig. S8 Collapsed molecular phylogenetic tree of the kinase domain in connectitin, DAPK, and trio/kalirin families.** All bifurcations of Fig.9 below cutoff 30 were collasped. Hs, Homo sapiens (human). Bf, Branchiostoma floridae (Amphioxus). Sk, Saccoglossus kowalevskii (Acorn worm). Sp, Strongylocentrotus purpuratus (Sea Urchin). Dm, Drosophila melanogaster (Fruit fly). Pc, Procambarus clarkia (Crayfish). Os, Octopus sinensis (Octopus). Cg, Crassostrea gigas (Oyster). Ef, Eisenia fetida (Earthworm). Ct, Capitella teleta (Ragworm). Ce, Caenorhabditis elegans (Roundworm). Eg, Echinococcus granulosus (Echinococcus). Dj, Dugesia japonica (Planaria). Aa, Aurelia aurita (Jellyfish). Hv, Hydra vulgaris (Hydra). Am, Acropora millepora (Coral). Nv, Nematostella vectensis (Sea anemone). Ta, Trichoplax adhaerens (Trichoplax). Aq, Amphimedon queenslandica (Demosponge). Hst, Hymeraphia stellifera (Demosponge). Om, Oopsacas minuta (Glass sponge). Ml, Mnemiopsis leidyi (comb jelly Lobata). Bfo, Beroe forskalii (comb jelly Beroe). Bm, Bolinopsis microptera (comb jelly Lobata). Hc, Hormiphora californensis (comb jelly Cydippida).
