# Supplementary material for: The Ancestor and Evolution of the Giant Muscle Protein Connectin/Titin
Source: J Mol Evol. 2025 Apr 27;93(3):306–21. doi: 10.1007/s00239-025-10247-7 (PMC12198301; doi:10.1007/s00239-025-10247-7)
Supplement: Supplementary file 2 — Supplementary file2 (DOCX 52 KB) [file 239_2025_10247_MOESM2_ESM.docx]

**Supplementary file 1. Alignment of the SEC14 domains used for molecular phylogenetic analysis**

10 20 30 40 50 60 70 80 90 100

....|....|....|....|....|....|....|....|....|....|....|....|....|....|....|....|....|....|....|....|

**Aa-CON(GHAI01170451)**  **ILNLKLAYLSGGLDKNGRCIITIPS-NK-EQK--------ATAEATSRYEELSTTLQYLDKIPSQD---HKERGFMVILDLR--GNAWK-ETKNLLSII-**

**Aa-DBS(GHAI01145310.1)**  **LLQKKLAILPGGLDRDGYPLLIFYD-IA-LDI--------ASI----DNGEFLKLTEYLSAIARTL---WNVDYLTLVVDRR--RAKWQ-TVKSLFLKL-**

**Aa-TRIO(GHAI01159172.1)**  **LLIEKAAFVTGGKDQNGAIYITVPK-YE-END--------DSL----FIDQFSHLLNYLASTRSIE---EKAFGFSFLVDLR--AATWS-EIKPIMKLI-**

**Am-CON(XP_044173189.1)**  **TLRAKLAFTSGGRDRQGRGILTIPAGKT-ED-----------L----SVDQLGDTLSYLTQLPRDQ---TRTLVYTVIIDLR--GSSWK-SAKPLLRTL-**

**Am-DBS(XP_044180590.1)**  **LLQEKYAFLSGAQAKNGCLIITMPE-HP-KF---------GSL----SDRDAERIFRYLVGLARED---LFHRGFMLVLDRR--REKWG-TVKSCLNKV-**

**Am-TRIO(XP_029203193.2)**  **LLCEKIAFLHGGKDRRGGPIISFPA-KS-RA---------FEL----DRISLTRLLVYLASIPSNH---ERDLGFSLVLDMR--GNTWQ-GAKPMLKAL-**

**Aq-TRIO(XP_019853833.1)**  **LLQMQLAVMPGSQDNNGHSVIVIHA-VE-QQS--------F------TVFNLGKILCYLKEITSDQ---VRKFGFTILVDVR--QGTWS-MIKKTLEVI-**

**Aq-TRIO-like(XP_019859647.1)**  **ILELKLAILSGGRDEMGREIIVFPA-RP-AV---------K-Y----DHKQILQTLQYLSSIPSPE---ALELGFTVIVDGR--WLSWP-DIKLILRTT-**

**Bf-DBS(XP_035693615.1)**  **QLQARFAYLTGGKGRNGAPIITLPE-YP-RF---------QEI----QDQDFLSVMTYLTSIPSAE---EAELGYVIVVDRR--QDRWA-AVKATLIRI-**

**Bfo-DBS(GHXY01284231.1)**  **ILESGLIVYPGTTDRSGKPLLFANLTFL-SVL--------EHN----SATELNAFFSYFVSISKED---VRENGYVFVVDCT--KRLAS-SVTDFLELL-**

**Bf-SESTD1(XP_035660906.1)**  **VLRKRICFLSGGRDKRRGPIITFPT-HP-KP---------VDF----SPEEIGLNVAFLASVPSPE---SRSRGFSVIIDVR--APTWR-ANKSLVKVL-**

**Bf-TRIO(XP_035662010.1)**  **ILREKIAFLSGGRDMRGGPVLTFPQ-RN-NPE--------KAI----RYDDLKRLMTYLASIPSED---TKKHGFSVVVDMR--GSTWS-SVKPLLKAL-**

**Bm-DBS(GKLW01034562.1)**  **LLESGLIHFPGSVDRLRNPVLFGKLELL-SFL--------ENN----SAVELNKFFNYFVSISKED---VKENGFVFVLDST--KGSAT-GLSDFLELL-**

**Ce-DBS(NP_741736.2)**  **ILATRYAFISGARTTEGLTIVTFPD-SR------------STL----PFEDYSLLVKYLLQVPPLE---DSHKGFVIIIDRR--SDKWS-SVRTLLLQI-**

**Ce-TRIO(NP_001021496.1)**  **VLRDGIAVLPGGRCRAGQAVIVCPS-RE-QP-----------V----NQDNLRNVFLYLFEVTSKM---AREKGFLVVIDMRG-KQTWT-NVRHILKAL-**

**Cg-DBS(XP_034327840.1)**  **LIQSRYAYITGGKAKNGALILTFPE-NP-QH---------PQI----PDEEYRKLIHFLCCVPTVA---ESEQGFVIVLDRR--QASWN-DVRALLLKM-**

**Cg-SESTD1(XP_034304301.1)**  **ILKKEIVYLTGGRDRQGGPLLTFPP-QP-DN---------PDF----NSHDITICLKYLLQIPSEE---SKRRGFTAIVDSR--DGSWQ-NLVTVLGCL-**

**Cg-TRIO(XP_034303731.1)**  **ILRERVAYLSGGRDRRGGAILTFPS-LT-HP---------EKL----DADDLRRLMTYLASVPSNE---VRERGFTMIIDMR--GSTWQ-IVKPILKVL-**

**Ct-DBS(ELU03899.1)**  **VLQARHAYVSGGKSPAGCPILTFPDITS-IT---------EEL----SEDDYQSLVSYLTSIPALE---DIDAGFVVVIDRR--QDRWT-AVKNLLLRI-**

**Ct-SESTD1(ELT90999.1)**  **ILKREAVFLTGGRDRRGGPVLTFPA-SN-DS---------GQV----SYEDLAT--DYLCSLPSTE---SAKLGFTVLFDNR--EHHYK-EVKPLLKLL-**

**Ct-TRIO(ELU10048.1)**  **LLRDNVAFLSGGRDKRGGAVLTFPA-HP-GL---------EQM----KTEDLRSLVQYLSAVPSDD---VRERGFTVVIDMR--GSTWQ-NIKPILKVLQ**

**Dj-TRIO(IAAB01074650.1)**  **LLKHGIALLPGGRDKRGGPVLCFPA-NS-HA---------DEL----DIEDLYVLVRYLTFLPDEN---VKKLKFSVVIDMRA-GTTWH-SVKPILKVL-**

**Dm-SESTD1(NP_001137872.2)**  **ALQTRSAYLSGGFDRQKRIVFVVNA-FN-DL---------QLW----NRRYLQVTLDYLKRSLSAS---VLQNGVSVVVNAQ--ESSSR-ISRQQVRQI-**

**Dm-TRIO(NP_651960.2)**  **LLQERVVFLTGGRDRRGGPLLCFPA-TP-RR---------DRL----KPEDLRRLLSYLISIPSDA---AKNLGFTVIIDMRGNGNCST-NVKTILKVL-**

**Ef-DBS(GIUK01066985.1)**  **VLQAKYALLSGGKSRRNFQILTFPE-CP-VA---------NTI----SDEQYRNVVTYLASIPPLH---EADLGFVVIIDRR--LDKWG-SVKTLLLRI-**

**Ef-SESTD1(GIUK01007937.1)**  **ILRMKAVCLTGGRDRRGGPILLFPS-DS-RY---------SEL----TARDLSACLVYLGSVPSEE---SVKKGFTVLIDNR--KGTFDGDLSTLAANV-**

**Ef-TRIO(GIUK01052821.1)**  **LLKEKIAFLSGGRDKRGGAILTFPA-RP-SQPSDQKS---DQV----RYDDLKTLVMYLASVPSDD---VKKLGFTVIIDMR--GSTWH-NVKPILRVL-**

**Eg-TRIO(XP_024355080.1)**  **LLRGKYAMLPGGRTRNGGPILCFPA-NS-HA---------DFL----PLEELYFLVLYLTYLPEEN---VKKLGFAVIIDMRS-GTTWH-SVKPVLKVI-**

**Hc-DBS(GHXS01017625.1)**  **LLESGVLLFPGSVDKQRNPVIFVRLELL-KFL--------DNS----SATELRAFLSYFVNISRPN---VQENGYVFLVDCT--KASSPGNFSDFTELL-**

**Hs-KALRN(NP_001375348.1)**  **ILKEKVAFVSGGRDKRGGPILTFPA-RS-NH---------DRI----RQEDLRKLVTYLASVPSED---VCKRGFTVIIDMR--GSKWD-LIKPLLKTL-**

**Hs-MCF2(NP_001165347.1)**  **FLMQDIAFLSGGRGKDNAWIITFPE-NC-NF---------RCI----PEEVIAKVLTYLTSIARQN---GSDSRFTIILDRR--LDTWS-SLKISLQKI-**

**Hs-MCF2L(NP_001106203.2)**  **QLKKRFAYLSGGRGQDGSPVITFPD-YP-AF---------SEI----PDKEFQNVMTYLTSIPSLQ---DAGIGFILVIDRR--RDKWT-SVKASVLRI-**

**Hs-MCF2L2(NP_055893.4)**  **QLHRQFAILSGGRGEDGAPIITFPE-FS-GF---------KHI----PDEDFLNVMTYLTSIPSVE---AASIGFIVVIDRR--RDKWS-SVKASLTRI-**

**Hs-SESTD1(NP_835224.3)**  **ILKKKLAFLSGGKDRRSGLILTIPL-CL-EQ-----------T----NMDELSVTLDYLLSIPSEK---CKARGFTVIVDGR--KSQWN-VVKTVVVML-**

**Hs-TRIO(NP_009049.2)**  **ILKEKVAYLSGGRDKRGGPILTFPA-RS-NH---------DRI----RQEDLRRLISYLACIPSEE---VCKRGFTVIVDMR--GSKWD-SIKPLLKIL-**

**Hst-TRIO(GKDX01093008.1)**  **PLRTKLVILSGGRDKRGAPILTISA-TD-HS----------NL----RPINLGMILSYLAKVPDES---LQGLGFTVILDMR--HASWD-TTKRALKSL-**

**Hst-TRIO-like(GKDX01095742.1)** **LLQQEIAILSGGRDRQGQSIITFPS-KD-KG---------FVY----ERDTVRRLVQYLARIPTED---VREHGFSVIVDAR--TSNWH-NTKLILRAL-**

**Hv-DBS(XP_002170266.3)**  **VLQQKVMYISGGKDKEGHPLILCTT-SP-IL---------GSV----SEDTFKKAVIYLEKTACVL---WENQKFVIIVDSQ--NDKWA-NVKVLMKLL-**

**Hv-TRIO(XP_047137671.1)**  **DLLGNLAQITGGYDRKGFLFLTILK-NA-CQ---------EEA----KIEDVKKVILYLASTRSSE---EKQKGFSFLVCLKP-TPPFG-NVKPITKLL-**

**Ml-DBS(GFAT01108065.1)**  **LLESGLLHFPGSVDRLRNPVIFGKLELL-SFL--------ENN----SAVELNKFFNYFVSISKDD---VKENGFVFVLDCT--KGSAT-SLTDFLELL-**

**Nv-CON(XP_048579266.1)**  **TLREKTAFLSGGYDRQGRAIITILV--P-KH---------EDI----DTEKLSETLIYFTQIPSEE---VLNRKFTFVLDLH--EGTWK-AAKALLKTL-**

**Nv-DBS(XP_032227566.2)**  **LLQMKSVFLSGGKDKSGCLILTIPP-GPASL---------TQL----SDQDTEDLFRYLISLPRSE---EKTDGFTFIIDKR--KSKWS-SLNNTLSKL-**

**Nv-TRIO(XP_032241348.2)**  **LLRQQIAFISGGIDRRGGRIITLPA-HT-FQG--------EQQ----DEESIGQIVSYLANLPSED---ECRLGFSIILDMR--SCTWS-TAKPVLRSL-**

**Om-TRIO(KAI6661633.1)**  **LFLTNVAYLTGGRDTRGGAIIQINT-PT-IE-----------R----SPDELANLLNFIASIPDDT---IKMKGFSILIDCQ--SCPTH-VAKFTVKVL-**

**Om-TRIO-like(KAI6652169.1)**  **TIEKRLCIMAGGIDKKGRLILCIPA-QA-KE---------FCY----DADELRRLILYLADTPPLE---LKALGFIAVVDAR--HSPWT-SSKLILHTL-**

**Os-DBS(XP_036365545.1)**  **VLQSKYAFITGGKARNGAPILTFPD-VP-GI---------PEI----TDEQYKKVMIYLCTIPAKLRLYEVEKGFVIILDKR--NDGWG-TVKSILLKL-**

**Os-SESTD1(XP_036356634.1)**  **LLLKEIVHLTGGRDKEGGPILTFPM-HR-EP---------VNF----SQKDIHDCIEYLSQIPSEE---SQWLGFTVIIDNR--SETWS-DLYYLLEVL-**

**Os-TRIO(XP_029657616.1)**  **ILRERVAYLSGGRNKQGGPILTFPS-HT-HP---------ERL----KYEDLRRLMTYLASVPSDD---VREQGFTIILDMR--GSTWQ-TVKPILKAL-**

**Pc-DBS(XP_045623387.1)**  **LLHAQYAIITGGKSREGCPILTFPD-RG-NF---------AQL----ADEEYRKLIIYLTSVPSLQ---DADMGFVLVIDRR--NDKWN-SVKTVLLKI-**

**Pc-SESTD1(XP_045612878.1)**  **LLERRLAVLPGSRDPGGGPILIIPL-PQ-DP---------SSH----DSGAISATVKYLKTIPSVS---ARERGWVVVVDAR--VCHYR-LVKPTVSTV-**

**Pc-TRIO(XP_045604343.1)**  **ILHEKVAFLSGGRDQRPGPILTFPA-SS-RR---------DRL----KHDDYHPLLQYLMQIPSDE---VREAGFTVIIDMR--GSTWN-TVKPILKEL-**

**Sk-DBS(XP_006811250.1)**  **LLQANIAYISGGHTEEGFPIITFPE-CP-VF---------NTV----SDKDFVRVMTYLTTVPSLQ---EVDLGFVIIVDRR--EDKWS-SVKSALGKI-**

**Sk-SESTD1(XP_006823022.1)**  **ILRSQNAYLSGGKDHRGGPILSIPI-VS-DP---------SEF----SPQDFATCIAYLAKIPSDE---AKSHGFTILIDAR--GSTWN-SVKPLLRIL-**

**Sk-TRIO(XP_006819273.1)**  **ILKDKLAFLSGGRDKRGGPILTFPA-RS-NL---------DKI----KYDELRRLMMYLASIPTED---VRAIGFSVIIDMR--GSTWN-GVKPLLKVL-**

**Sp-DBS(XP_030837678.1)**  **QLNHRVAFITGGKTQEGYPIINMPE-CP-KF---------NLV----RDEDFVRLMTYLVRIPSRL---EFDRGFVIIIDRS--TNSWG-DVKTTLIRIA**

**Sp-SESTD1(XP_030830043.1)**  **LLKSRLAYLSGGKDRHGGPVISFPS-QA-SE-----------V----SIEDIATTITYLASLPSTE---SKAHGFSLLVDMR--GSAWQ-PIKVVMKVL-**

**Sp-TRIO(XP_030830126.1)**  **LLRDKLAFLSGGRDKRGGPILAFPS-GS-AL---------DKV----SPIDLRKLVFYLSGIPSDE---ARDLGFTVIIDMR--GSTWT-NIKPVLKAL-**

**Ta-CON(GHJI01001904.1)**  **ILRSRIAFLSGGVDKRGGPVITFPA-QA-RTQ--------QEI----SITELTSTILYLSKIPSDD---SRRHGFTVIIDVR--GRTSK-LLKPILRSL-**

**Ta-DBS(XP_002107613.1)**  **AIRMKFAYLTGTRTKTGCPILSMPL-FN-HFG--------TVV----TDDLLKEVIQYFVDSTMVH---SSSKRFAVVLDRR--SSSWN-SVKSLLRKL-**

**Ta-DBS(XP_002108429.1)**  **ILRIRYAYLSGGR-YCGSPIIIFPA-SS-QKFDFDSDDNQKTV----TEEELEILIRYLMRSISEH---DRENGFVFVLDRR--KSSSV-TIKTLLRTI-**

**Ta-TRIO(GHJI01000868.1)**  **ILKEKIAFLSGGRDRSGRAIITFLA-RV-TF---------VDI----ESERLIKLINYLASIPCQE---VKVKGLTVIVDGR--SVDWR-GLKPLLNIL-**

110 120 130 140 150 160 170

....|....|....|....|....|....|....|....|....|....|....|....|....|....|....|....

**Aa-CON(GHAI01170451)**  **---ADSLSC-VKQ-VLIIK---PDDFWEKRRSSFGYKIQR--SGLGFDT--VLLSTPAKIFQYC-EQTEIVSDFGGFLQ**

**Aa-DBS(GHAI01145310.1)**  **---KGINSFQIHK-ILLLK---PKGFFQKHFGNDKKEIQ---ELFDCKI--VFLDNQDSLFNHV-DVRQLPEDVGGVFE**

**Aa-TRIO(GHAI01159172.1)**  **---QDFFAFKVNN-AYVLK---PDGFGGLQRASFST------AKFTFEI--TLTS-SEGLTKYI-STRQLTPEYFGTLN**

**Am-CON(XP_044173189.1)**  **---QDTVPDRVRD-VYIIK---PDAFWEKRRSGTGLIKEQ--SSVVFET--TVLSSASKLHQFA-EDSQLTQELGGTLP**

**Am-DBS(XP_044180590.1)**  **---QKCFPAKISL-VCLIK---PQGFLQK-YSKPGFVTDGIIGDAKIQV--VSLNSVAELDGYI-EKSQLTADVGGTLD**

**Am-TRIO(XP_029203193.2)**  **---QECFLAKIHT-VYLVK---PDGFWESRRTSFGS------SKLIFET--VVTS-LDGLTKFI-DLDHLTSDLGGSFK**

**Aq-TRIO(XP_019853833.1)**  **---KVTFVEYVHQ-VIIMK---PKSMWQRGRSSVNFSFKK--SKYDFKC--ILLEHSADVFHYI-SRSNVPTYQNGDFE**

**Aq-TRIO-like(XP_019859647.1)**  **---QEALPGAVHV-AYVLQ---PNQFMKKKSVSLSLSKER--DKLEFTI--VTASSTDKLLRHI-DGRQIPVELGGAIN**

**Bf-DBS(XP_035693615.1)**  **---AGFFPKHIQV-VLVLR---PTGFFQRTFSDIGFKFVR--EDFRLKVPMVMLNSVSELHEHL-DRSQLTEELGGFIR**

**Bfo-DBS(GHXY01284231.1)**  **---HDYFPGRVSK-VYVVLNSPHDGYLGKAYLKMKSMLASMQGNNDFPR--VDVNCFTELHRFV-DPSQIPSVFEGRLN**

**Bf-SESTD1(XP_035660906.1)**  **---QQTLGRQLAQ-VIIVK---PDTFWDKQKTSFRYRNDK--AEGGYET--IMVT-ASKIFQYV-DQDQLTKDLGGTLP**

**Bf-TRIO(XP_035662010.1)**  **---QDCFPGNIHM-AHIIK---PDNFWQKHRTSLGS------SKFSFET--SMIA-LDGLTKCI-DQSQLTSEFDGTLV**

**Bm-DBS(GKLW01034562.1)**  **---HDYFPGKINK-IYVIQNPTEDGYVGRAYLRVKSFLNNMQGSADYPR--VTLNSYSELERVI-DPSQIPSFFDGHLD**

**Ce-DBS(NP_741736.2)**  **---SSFFPGKICV-TFVIK---PEGVLQRAL-EVGYRGAA--DTCSFQV--IQLESSAELRKYI-HHEFLTMDVGGLIK**

**Ce-TRIO(NP_001021496.1)**  **---SSIESSSTVQ-VFIIK---PEKFWEKQKAQMSL------GTWDFEV--EMIS-FESLIKII-DSSHLPKTVGGSYP**

**Cg-DBS(XP_034327840.1)**  **---SEFFPKHIQV-VFLLQ---PKGFFQRAFADMKSKFVK--EELEFKV--VLCNEPAEMFDYI-SEDQITTDVGGQLE**

**Cg-SESTD1(XP_034304301.1)**  **---KQSLGDYMKQ-VLVLK---ADQQMDRRSSSSSFRTRDR-NNPNLEP--QFVN-LQRLHTYV-DKNQLTHSYGGLLW**

**Cg-TRIO(XP_034303731.1)**  **---QDCFPDSINI-AYIIK---PEKFWEKKRTSLGS------AKYNFET--CMIS-VDGLSKFI-DCSQLTREFEGSLE**

**Ct-DBS(ELU03899.1)**  **---SGFFPGLVQQ-VYVLK---PSSFLQRNLADVGFKFVR--DDFKFKV--LMCGSADELHKSI-PVDQLTFDLGGSLE**

**Ct-SESTD1(ELT90999.1)**  **---QDALGSSVFA-VYIVQ---PE----KGKLNFSFRRE---RNLSIEP--QNIT-VAKLKNII-QPNQLSIELGGSLD**

**Ct-TRIO(ELU10048.1)**  **---QECFPHQIHA-AYLIK---PEKFWEKQKTSIGS------AKYKFET--TLIS-VDHLHKLI-HPSELTMDFDGTLP**

**Dj-TRIO(IAAB01074650.1)**  **---EECIGQCTSS-VYIIK---PDKFLEKQKAQMAC------SKFTFDI--QIVS-VNVLYQEI-DQSQLTAHFDGSLP**

**Dm-SESTD1(NP_001137872.2)**  **---YALFGGDINVDLYLVR---AEGFWEKHVEPCTK------SQVKGEP--LVLS-KARLFKFI-EPQNLPEELGGTLQ**

**Dm-TRIO(NP_651960.2)**  **---QEHFSANIHN-VVIIK---PDNFWQKQRASISS------HKYKFET--TTVS-IESLNKIV-ESHQLTGDFEGQQL**

**Ef-DBS(GIUK01066985.1)**  **---SGYFPALVQK-VYLLR---PSSFLQKTFADMGFKFLK--EDFKFKI--LMLNSVEELHDHV-DRDQLTDDLGGSLV**

**Ef-SESTD1(GIUK01007937.1)**  **---TRVLSGAVHA-IYVVQ-----VTKSKSVSDLNQ------NQFQFVT-------PSTLNNIC-EEGELSADLGGRLN**

**Ef-TRIO(GIUK01052821.1)**  **---QECFPHNIHT-AYIIK---PEKFWEKQKTSLGS------SKYKFET--NLLS-VDSLTRVI-HPSQLTEEFDGTLS**

**Eg-TRIO(XP_024355080.1)**  **---EECIGGNVAM-AYIIK---PDKYLEKQKVQMSI------GKFSFNI--QLVS-VEQLFLEV-DASQLTADLEGTLP**

**Hc-DBS(GHXS01017625.1)**  **---RDFFSAKINK-IYVVQRSPEESYVGRAYLRVKNLLSNMQGSVSYPQ--VTLTGYEDISRVV-DLNQVPSYFDGHMD**

**Hs-KALRN(NP_001375348.1)**  **---QEAFPAEIHV-ALIIK---PDNFWQKQKTNFGS------SKFIFET--SMVS-VEGLTKLV-DPSQLTEEFDGSLD**

**Hs-MCF2(NP_001165347.1)**  **---SASFPGNLHL-VLVLR---PTSFLQRTFTDIGFWFSQ--EDFMLKLPVVMLSSVSDLLTYI-DDKQLTPELGGTLQ**

**Hs-MCF2L(NP_001106203.2)**  **---AASFPANLQL-VLVLR---PTGFFQRTLSDIAFKFNR--DDFKMKVPVIMLSSVPDLHGYI-DKSQLTEDLGGTLD**

**Hs-MCF2L2(NP_055893.4)**  **---AVAFPGNLQL-IFILR---PSRFIQRTFTDIGIKYYR--NEFKTKVPIIMVNSVSDLHGYI-DKSQLTRELGGTLE**

**Hs-SESTD1(NP_835224.3)**  **---QNVVPAEVSL-VCVVK---PDEFWDKKVTHFCFWKEK--DRLGFEV--ILVS-ANKLTRYI-EPCQLTEDFGGSLT**

**Hs-TRIO(NP_009049.2)**  **---QESFPCCIHV-ALIIK---PDNFWQKQRTNFGS------SKFEFET--NMVS-LEGLTKVV-DPSQLTPEFDGCLE**

**Hst-TRIO(GKDX01093008.1)**  **---QLTFVEHIFQ-VYVVK---PKRFWQRQKTGMNFHMKR--SKFNYPV--VMLENGGDLFHYF-QVSQITRELGGMLS**

**Hst-TRIO-like(GKDX01095742.1)** **---QEVLPGQIHV-AYIIQ---PGHFWQKKATTRGHSKEK--SKLEFST--VMLSAVEKLIRHV-DSRQLTSDLGGFLP**

**Hv-DBS(XP_002170266.3)**  **---EDFEACYIHY-ILLIK---PQGFVQRHFSVKDTKQLQ--EFTKHPI--VILESIEKLYNYV-ELNELPADLGGSNN**

**Hv-TRIO(XP_047137671.1)**  **---QDGFPFKIFA-VYIIK---PEKYKEKILSSSMAS-----SKYRFEI--NVVS-KENVFTFI-APEELTEDLGGTYI**

**Ml-DBS(GFAT01108065.1)**  **---HDYFPGKINK-IYVIQRPTEDGYMSRAYLRMKSFLSNMQGTTDYPR--VTLDSYSDLQKII-EPSQIPSFLDGHLD**

**Nv-CON(XP_048579266.1)**  **---QEVVPDKIHQ-IVIIK---PDAFWEKRKSDTSI------KTTDHET--TVLSSSSKLTQIA-DEAHLTRDYGGTLP**

**Nv-DBS(XP_032227566.2)**  **---QKTFPDRVNC-VYVLK---PQGFMQRFIGGSLSSED---AQAAFRI--VVLGTVSDLLKYV-TRDQLTTDLDGEFD**

**Nv-TRIO(XP_032241348.2)**  **---QECFPEKIHS-VYIIK---PEGFWERHKTNLKT------SKLSFET--TLTS-LDGLAKFI-EPSQLTASLGGTLK**

**Om-TRIO(KAI6661633.1)**  **---QQLFNEKIST-VFLLK---PNNFWERRRSGMSFRSK---SKYSFPV--ELLNSIKDIQHFFPDSTQYLPQHGGSLV**

**Om-TRIO-like(KAI6652169.1)**  **---KDALSSHLLK-VYLLQ---PDQFWKKKQASIGHSKEK--TKLPFET--ILLSSLEKLARYL-DLKQLPAEMGGTLP**

**Os-DBS(XP_036365545.1)**  **---SAFFPTHIQV-VFLLQ---PHGFLQRALADFRSKFVK--EELEFKV--VMCNNQEELHEHI-DPSQLTKDLGGEIE**

**Os-SESTD1(XP_036356634.1)**  **---KKSLLARLKR-ILILK---TSPEDTPRHLNIKYS-----DAIQFVT-------LKQLTNFI-SPYQLTENLGGQLP**

**Os-TRIO(XP_029657616.1)**  **---QECFPGNIQM-AFIIK---PEKFWEKQRTSLGS------AKYNFET--NMLS-VDNLSKFI-DSSQLTREFDGTLD**

**Pc-DBS(XP_045623387.1)**  **---SGFFPGLITV-AYVLR---PAGFFQKAISEVSNKIFR--DEFKFRV--VVCNCVGDLHEYI-DKNQLTEDLDGCIP**

**Pc-SESTD1(XP_045612878.1)**  **---RATL-GYIRH-LFVVR---PEGFWDKQRVDCRK------SEVDSQP--IYVS-VSKLTRHM-EVSQLLVELGGTLD**

**Pc-TRIO(XP_045604343.1)**  **---AESFTPHIYS-VFIVK---PDNFWQKQRTSIGS------HKYKFET--NLIS-VETLAKTI-DPNQLTVDFDGTLP**

**Sk-DBS(XP_006811250.1)**  **---AGYFPGTVRE-VFVLR---PSGFFQRTFTDVGFKFYR--DEFKFKV--FMLESLKELHDHF-DKSQLTTQLEGDIS**

**Sk-SESTD1(XP_006823022.1)**  **---QQVLPEKIFL-ACIVK---PEAFWEKQRAGQSLKKEK--SQLDFEV--LMFSSVAKLHKYC-SADQLTPLFGGTLR**

**Sk-TRIO(XP_006819273.1)**  **---QECFPSSVHM-VYIIK---PENFWQKHRTSLGS------SKLKFET--SMIS-QEGMLRII-DQSQLTIEFDGHLP**

**Sp-DBS(XP_030837678.1)**  **DYYQGYFPGIIQG-VYVIK---PRGFLQKTFSEMRFKGLK--DEFKFKI--DLVDSTTELHARI-DPTQLTNEFGGTLN**

**Sp-SESTD1(XP_030830043.1)**  **---QETLAAGIST-IYILK---TD---ERQRSNLGIKKD---KSQMFET--IILSSPSKLTKYL-DTSQLTEEFGGSFD**

**Sp-TRIO(XP_030830126.1)**  **---QECVPDNIHM-VYIIK---PEKFWQKQRTSMGS------SKLKFET--AMIS-SENLVKMV-DPAQLTVDMEGTMH**

**Ta-CON(GHJI01001904.1)**  **---QACLVGQVYM-VCVIR---PDSFVERQKSNVKVKD----KSLSFEV--ITMNGTSKLHRYV-ANDQLTSDLHGNLA**

**Ta-DBS(XP_002107613.1)**  **---QELFGRYIDT-LYLLK---PQKMMA-VLGGVSK------SEFSFQI--MLLNTIDELHQNI-GGIFLTQEFGGFLK**

**Ta-DBS(XP_002108429.1)**  **---ETLFPTEIIK-LYVLR---SPKTYKKRLAEIGRQSTR--PNFSINV--VYVHSLEELHQEI-NIDQLSTELGGTLN**

**Ta-TRIO(GHJI01000868.1)**  **---EEAFLDTIAK-VFVIK---SDSFWKRGKAMITQ------MKTELNM--QVVS-ISNLVKQI-DITDLTEELQGILP**
