# Supplementary material for: The Ancestor and Evolution of the Giant Muscle Protein Connectin/Titin
Source: J Mol Evol. 2025 Apr 27;93(3):306–21. doi: 10.1007/s00239-025-10247-7 (PMC12198301; doi:10.1007/s00239-025-10247-7)
Supplement: Supplementary file 3 — Supplementary file3 (DOCX 176 KB) [file 239_2025_10247_MOESM3_ESM.docx]

**Supplementary file 2. Alignment of the kinase domains used for molecular phylogenetic analysis**

10 20 30 40 50 60 70 80 90 100

....|....|....|....|....|....|....|....|....|....|....|....|....|....|....|....|....|....|....|....|

**Aa_CON_A(GHAI01170451.1)**  **-Y-TLK--DEL-GRGKFGVVSQ-CVHKETSKE-YAAKFVKCRP--------------REKAAVYSEIKIM---NEL-H--------H-KRLVQLYDAY--**

**Aa_CON_B(GHAI01170451.1)**  **-Y-KFG--DEI-ARGKFAVVKK-CTDKRTGET-FAAKLVKYDE--------------DTKEVTKKEFQIW---NGL-S--------H-PNLLVLHDAY--**

**Aa_TRIO(GHAI01159172.1)**  **-Y-SIS--SEL-GRGRFSFVKK-CVEKATGKE-YAAKLVKKRV--------------LDKEMVENEIAIL---RVL-N--------H-PSLCTIHDTF--**

**Am_CON_A1(XP_044172413.1)**  **-Y-SPI--KEL-GKGKFGVVKK-VKEKSTGTE-YAAKFLKITK--------------ESREEVLAEMLIM---NQL-H--------S-KRLIYLHDAY--**

**Am_CON_A2(XP_044172413.1)**  **-Y-TVK--DEL-GKGRFGVVYK-CIDNKTGKE-YAAKFVKCSS-------------PKERQDVVHEAEIM---TSV-R--------H-KRLLRLQDFF--**

**Am_CON_B(XP_029213098.1)**  **-W-GIK--HEL-ARGRFSVIKR-CLDNKTKKP-YVAKLIKYDD-------------DTDLEDTLQEFEIQ---RSI-K--------G-DKVVMLRDAF--**

**Am_DAPK(XP_029207283.2)**  **-Y-DIL--DEI-GSGQFAVVKK-CSEKSTGTE-YAAKIMKKKR-------RRGSRRGVSTEEIIREASIL---LKT-R--------H-EGVIYLHAVF--**

**Am_TRIO(XP_029203193.2)**  **-H-NIY--AEL-GRGRFSVVKK-CVEKSTGNE-YAAKLVKKRM--------------VGKDEIEDEIHLL---RRL-K--------H-PNLTNLIDYF--**

**Aq_TRIO(XP_019864448.1)**  **-Y-LLK--NEI-AKGMFGTVYK-CLHKGTQQI-YAAKLIPINL---------------QINHCQNELDIL---ALI-C--------H-PNIVQLSAAY--**

**Aq_TRIO_like_A(XP_019854769.1)** **-YELLS--GEL-GRGRFGIVRS-CVSKVTGSA-AAAKYLKESH------------------DVLQEVKIL---RNLSS----GPSPS-PHVILFIDAL--**

**Aq_TRIO_like_B(XP_019854769.1)** **-Y-EII--NEI-GRGSFSVVYE-CTELTTDRP-FAAKFIKCSN-------------DDQFSVALREFEMI---KNV-T--------H-PRIASLEDAF--**

**Aq_STK17(XP_003383221.1)**  **-Y-DVG--AEL-GRGKFAIVKR-VTEKASGEQ-FAAKFLRKRR-----------GGKACRDDIIVEVDIM---RQSMG--------H-HRIIKLREVF--**

**Bf_CON(XP_035661587.1)**  **-F-DFK--EEL-GRGAFGAVYR-VVERSTRRT-FAATVVTCKT-------------SERKQLCRREIELL---HKL-N--------H-ARVLRLYDAY--**

**Bf_DAPK(XP_035665897.1)**  **-Y-IVG--EEI-GSGHFAVVKK-VVCKRSGTE-FAAKFIRKKR-------ASTSRRGARREDIEREISIL---QEL-N--------H-VNIIKLYDIF--**

**Bf_TRIO(XP_035662010.1)**  **-Y-SEI--SEI-GRGRFSVVKM-CCHMGSKRE-VAAKFISKKY--------------LTKEAADNEVSIL---QSL-Q--------H-PHLNTVHEAY--**

**Bf_MYLK(XP_035660911.1)**  **-Y-DVK--EKL-GEGRFGKVYK-AVQKSSGSE-FAAKQLCVSD-------------PCQMEQVLQEVEIM---RLL-D--------H-PKLIQLADVF--**

**Bf_MYLK2(XP_035661607.1)**  **-Y-DVK--DKL-GQGRFGTVYL-CEDKSTRRR-FAAKYVRCRR-------------SADRQSINHEIDIM---NQL-R--------H-PRILQLYDAF--**

**Bf_OBSCN_A(XP_035660917.1)**  **-Y-RIH--EEL-GKGAFGIIKR-VTHRKTGKK-FAAKYIRYKP--------------HRKADLQREVTVM---AKL-E--------H-AGIVQLAETF--**

**Bf_OBSCN_B(GESZ01107032.1+XP_0** **-Y-NFE--EDV-ARGRYAVVKK-CVDIITSKE-YVSKVIPITP--------------VNKLEKLREYEAL---SKL-R--------H-GKVLELRDAF--**

**Bfo_TRIO_like(GHXY01112106.1)**  **-Y-KFT--DIL-YYGHFSMFCT-CEHVRTGLR-FTAQVTPVVD-------------RQFYRSVQREVDIL---KQI-N--------Y-KRVSRLHEVI--**

**Bf_STK17(XM_035832943.1)**  **-------------RGKFAVVKK-CRRKSSGQE-YAAKFIRKRK-----------KGKDCRETILAEIRIL---EMSAE--------H-CRLIDLFEVF--**

**Bm_TRIO_like(GKLW01050522.1)**  **-Y-KFT--DIL-FYGHFSVFCT-CEHQRTGLK-FTAQVTPVVD-------------RQFYRSIQREVDIL---QQL-N--------Y-KRVSRLHEVL--**

**Ce_DAPK(NP_490840.2)**  **-Y-EIE--TEL-GSGQFAVVRR-VRDRKTGEK-YAAKFIKKRR-------YATSRRGVTRQNIEREVRVL---QKIRG--------N-SNVVELHAVY--**

**Ce_MYLK(NP_509689.1)**  **-Y-QVT--KLL-GDGKFGKVYC-VIEKETGKE-FAAKFIKIRK-------------EADRAEVEREVSIL---TQL-R--------H-PRIAQIYDAF--**

**Ce_OBSCN_A(NP_001343714.1)**  **-Y-EIS--EKDEKLAAEGAPFR-VKEKATGRE-FLAQLRPIDD------------------ALMRHVDIH---NSL-D--------H-PGIVQMHRVL--**

**Ce_OBSCN_B(NP_001343714.1)**  **-F-QIG--GLK-FKGRFSVIRD-AVDSTTEGHAHCAVKIRHPS-----------------SEAISEYESL---RDG-Q--------H-ENVQRLIAAF--**

**Ce_titin(G4SLH0.2)**  **-Y-IIH--EEL-GKGAYGTVYR-ATEKATGKT-WAAKMVQVRP-------------GVKKENVIHEISMM---NQL-H--------H-EKLLNLHEAF--**

**Ce_twitchin(NP_502274.2)**  **-Y-DIH--EEL-GTGAFGVVHR-VTERATGNN-FAAKFVMTPH-------------ESDKETVRKEIQTM---SVL-R--------H-PTLVNLHDAF--**

**Cg_CON_A1(XP_034304307.1)**  **-Y-IIC--EEL-GRGAFGVVHR-AIERATNKN-WAAKMIRCKP--------------HEKEVVRHEIDMM---NEL-H--------H-PKLLQLHEAF--**

**Cg_CON_A2(XP_034304307.1)**  **-Y-DLQ--EAI-GKGKFGKVHK-CFEKTSGKT-FAAKIVKCRT-------------QKEKENLKQEVEIM---NLL-S--------H-PKLLMLWDAF--**

**Cg_TRIO(XP_034303731.1)**  **-Y-KEL--EEI-GRGRFAVVKK-CLQKCSNQY-VSVKYLNRRQ--------------TRKEEVEMEFNIL---HLL-Q--------H-ENLVQLYDLY--**

**Cg_MYLK(XP_011441364.2)**  **-Y-DVT--DLLLGRGKFGEVKK-CKEKKTGRF-LAAKFIAVNG-------------LQERDDVINEVDIM---KTL-Q--------H-PRLLQLYDAF--**

**Cg_OBSCN_A(XP_034299497.1)**  **-Y-YVL--EEI-GRGRHGIVRR-VLDKFTGSQ-YAAKFIHVCD-------------EHQRRFFRTELNVL---RWL-N--------Q-RGVPKVVNAF--**

**Cg_OBSCN_B(XP_034299497.1)**  **-Y-SFS--NIL-WRGKFSSYVR-STDKTNKNN-YITKITPCSD--------------SNKDSLTRELEFL---RTM-N--------H-ERFVRLYGAY--**

**Cg_STK17(XP_011422577.1)**  **-Y-TIQ--GEI-GRGKFAVVKK-CIHNETGEE-VAAKFIRKRR-----------KGKSCREEILREVVML---ELGLE--------H-PRLVDLKEVF--**

**Cg_twitchin(XP_034332726.1)**  **-Y-DIL--EEL-GSGAFGVVHR-CVEKATGRV-FVAKFINTPY-------------PLDKATVKNEINIM---NQL-H--------H-PKLLQLKDAF--**

**Ct_CON(ELT96026.1+AMQN01011565** **-Y-IVC--EQL-GKGPNGSVHR-VLERATGRS-FIAKTVESTR--------------TERTSVLQEAAIL---SEI-Q--------H-PKVVGLHDVL--**

**Ct_DAPK(ELT88671.1)**  **-Y-HIG--EPI-GSGQFAVVRK-CKLKETNVE-YAAKFIKRKR-------TKSSRRGLSIEDIQREVSIL---SAI-D--------H-ENIVKLYDVY--**

**Ct_TRIO(ELU10047.1)**  **-F-EEK--EDL-GRGRYSVVNR-VIQKHDGKE-MAAKFVNPKL--------------IGQDAVATEISIL---QKF-E--------H-AGIVRFIDAY--**

**Ct_MYLK(AMQN01000602.1+ELU1638** **-F-QVL--GFL-GRGKFGEVKR-CSETSTGQE-FAAKFISAPR-------------PQDKKDVHHEIDIM---KKL-Q--------H-RRLIQLYQAF--**

**Ct_MYLK2(ELT87547.1)**  **-Y-TTG--GEI-GKGRFGSVFL-CKEKQSNRE-FAAKFIKVKM--------------GQRDELRNEVMIM---NAL-H--------H-PKLLLLWDAF--**

**Ct_OBSCN(ELU05514.1)**  **-YFHVI--DEL-GRGSYGVVRR-VIDKNSGNQ-YAAKFLRYND-------------NFLKEDLMSELEVM---ATL-D--------H-PNIISAIDGY--**

**Ct_STK17(ELU16209.1)**  **----------L-FRGKFAVVRR-VTHRTSGKS-YAAKFLRRRR-----------MGKDCEHVAFEEVRML---ETALG--------H-PHLVHVIEVF--**

**Ct_twitchin(ELT89874.1)**  **-Y-DVY--EEI-GAGAFGAVHR-CIEKATGRT-FVAKFLNTPH-------------PADKMAVKNEINIM---NQL-H--------H-QKLLNLHDAF--**

**Dj_DAPK1(IAAB01043597.1)**  **-Y-KLG--PHI-GKGHYATVKI-CIDIETQKK-YAAKFIKKKR-------IATSKIGTKNDDIIREARIL---KKL-D--------H-PNVIKIYEAF--**

**Dj_DAPK2(IAAB01051648.1)**  **-Y-KVG--EKL-GHGHFAEVKH-CIDKNNDTE-YAAKFIRVRQ------VLDSKSIGMSLEEIEREAHIL---KML-N--------H-QNIIKLFFCY--**

**Dj_MYLK(IAAB01042781.1)**  **-Y-RIG--DLL-GSGKFGEVKL-CVEKTTKRE-FAAKIIAIST-------------TEDKINVEREIQIM---KVL-R--------H-RRLLQIYDAY--**

**Dj_STK17(IAAB01043480.1)**  **-YYFHG--KEI-GRGHFSKVYH-VTCLTDNKE-GAVKVIRRRR-----------GGIDRIQTIRQEIKIM---QDLMN-------NNIQSVIKLNFVH--**

**Dj_twitchin(IAAB01071515.1)**  **-Y-EIL--EEL-GSGAFGQVYR-CRELSTGKI-FVAKFIDTPH-------------QVDKNAVRHEIDTL---NHL-H--------H-PKLLNLHDAF--**

**Dm_MYLK(NP_001260832.1)**  **-Y-DVL--GEV-GRGKFGTVYK-CRDKANGLQ-LAAKFVPIPK-------------REDKRNVEREVEIM---NSL-Q--------H-HLIIQLYAAY--**

**Dm_OBSCN_A(XP_034109413.1)**  **-Y-DIG--DEL-GRGTQGITYH-AVERSSGDN-YAAKIMYGRP--------------ELRPFMLNELEMM---NTF-N--------H-KNLIRPYDAY--**

**Dm_OBSCN_B(XP_034109413.1)**  **-Y-SFI--SEI-ARGEFSTIVK-GIQKSTDTV-VVAKILEVTD--------------ENEDNVVAEFDNF---KTL-R--------H-ERIPALFSAYKP**

**Dm_projectin(NP_995598.1)**  **-Y-DIL--EEI-GTGAFGVVHR-CRERSTGNI-FAAKFIPVSH-------------SVEKDLIRREIDIM---NQL-H--------H-QKLINLHDAF--**

**Dm_STK17(NP_001162723.1)**  **-Y-EVE--QTPFARGKFAAVRR-AIHKNTGSH-FAAKFLKRRR-----------RAQSSDKEIKHEIAVL---MLCEG--------E-DNIVNLNAVH--**

**Dm_stretchinMLCK(NP_725510)**  **-F-EII--EEL-GKGRFGIVYK-VQERGQPEQLLAAKVIKCIK-------------SQDRQKVLEEISIM---RAL-Q--------H-PKLLQLAASF--**

**Ef_CON(GIUK01093179.1)**  **-Y-IIC--EEL-GRGPYGAVHR-AIERSSGKN-WVAKFVVCTT--------------IERAMVKQEVDIM---NDL-R--------H-PKLLQLHEAF--**

**Ef_DAPK(GIUK01016195.1)**  **-Y-NIG--EKI-GSGQFAIVKQ-CRHKETGVE-YAGKFIRKRR-------AKASRRGAAMEDIEREVTIL---KEV-D--------H-KNIVKLHEVY--**

**Ef_DAPK(GIUK01061946.1)**  **-Y-EVG--EEI-GTGKFATVRK-CKHRTSGVE-YAAKFIRKRR-------MKTSRRGAPLQDIKREVAIL---RQA-Q--------H-ENIAKLYEVY--**

**Ef_MYLK2(GIUK01050934.1)**  **-Y-ILG--EEI-GKGKFGSVYK-CTEKTTDRL-FAAKFIRVKA--------------GQRDEFRKEIEIM---NEL-H--------H-PKLLLLWDSF--**

**Ef_MYLK(GIUK01094627.1)**  **-F-KVI--DFL-GRGKFGEVKR-CVETSTGTE-FAAKFICTPR-------------PQERKDVEHEVSMM---RKL-R--------H-RRLVQLYDAF--**

**Ef_MYLK(GIUK01083042.1)**  **-F-QIL--DFL-GRGKYGEVKR-CTGLTSGME-FAAKFICTPR-------------PQDKKDVEHEVEMM---RRL-R--------H-RRLVQLYDAY--**

**Ef_OBSCN_A(GIUK01083331.1)**  **-YFHVI--DEI-GRGSAGVVRR-VIDKNSGNQ-YAAKFLRYND-------------ILLKDELSQELDMM---SLL-Y--------H-NNIVQVVDGY--**

**Ef_OBSCN_B(GIUK01083331.1)**  **-YLTFG--SEI-WRGRFSIVRN-VEPKEGSKIKRVCKISEYNA--------------SDPVNSLREYEML---KCV-N--------Q-EHIVRLYEAY--**

**Ef_STK17(GIUK01063378.1)**  **-Y-DVS--AEI-GRGKFAVVKR-CKHKTSGKE-YAAKYLKKRR-----------KGKDCKKEVLHEVHML---ELAIT--------H-PHIITVVEVY--**

**Ef_twitchin(GIUK01013637.1)**  **-Y-DIC--EEI-GSGAFGVVHR-VVEKATGRN-FVAKFINTPN-------------ASDKATVRNEINVM---NNL-H--------H-QNLLNLHDAF--**

**Ef_twitchin(GIUK01097374.1)**  **-Y-DIY--EEL-GTGAFGVVHR-AVEKATGKT-FVAKFINTPY-------------PTDKQTVKNEIGLM---NQL-H--------H-PKLLNLHDAF--**

**Eg_DAPK(KAH9282855.1)**  **-Y-ILG--EKI-GDGNFAEVKI-CECRKTGQK-FAAKIVKMRQKTVFGYYDPNSR-GMTPQEVEREVQVL---RSI-D--------H-HAIVKLHQIY--**

**Eg_MYLK(XP_024351289.1)**  **-Y-RIH--EFL-GSGKFGDVNR-CEEKSTGYE-LAAKVVPYSC-------------LDEKEGVMNEVGIM---CRL-R--------H-PRLIQLYDVF--**

**Eg_STK17(KAH9283880.1)**  **KS-SMP--ACL-PTGEFRPSFELVNDIPDGKS-LAIKIIRRFR-----------CGRDSIEKIRNEIELI---RTL-QSSNKDYNVPSAVAPLLFSVH--**

**Eg_twitchin(KAH9283145.1)**  **-Y-NIL--EEI-GTGSFGVVHR-AKEKATGRT-YVAKFVPTST-------------EAERNAVNNEANIM---KQL-N--------H-PKLLHLHEVF--**

**Hc_TRIO_like(GHXS01109180.1)**  **-Y-KFL--NTL-FYGHFSMFCT-CEHLKTGLK-FTVQVTPVKD-------------RQFLRSIQREIDIL---QSL-N--------F-KRVSRLHEVQ--**

**Hs_CON(NP_003310.4)**  **-Y-MIA--EDL-GRGEFGIVHR-CVETSSKKT-YMAKFVKVKG--------------TDQVLVKKEISIL---NIA-R--------H-RNILHLHESF--**

**Hs_DAPK1(NP_004929.2)**  **-Y-DTG--EEL-GSGQFAVVKK-CREKSTGLQ-YAAKFIKKRR-------TKSSRRGVSREDIEREVSIL---KEI-Q--------H-PNVITLHEVY--**

**Hs_DAPK2(NP_055141.2)**  **-Y-DIG--EEL-GSGQFAIVKK-CREKSTGLE-YAAKFIKKRQ-------SRASRRGVSREEIEREVSIL---RQV-L--------H-HNVITLHDVY--**

**Hs_DAPK3(NP_001339.1)**  **-Y-EMG--EEL-GSGQFAIVRK-CRQKGTGKE-YAAKFIKKRR-------LSSSRRGVSREEIEREVNIL---REI-R--------H-PNIITLHDIF--**

**Hs_KALRN(NP_001375348.1)**  **-Y-TEL--NEI-GRGRFSIVKK-CIHKATRKD-VAVKFVSKKM--------------KKKEQAAHEAALL---QHL-Q--------H-PQYITLHDTY--**

**Hs_MYLK(NP_444253.3)**  **-Y-DIE--ERL-GSGKFGQVFR-LVEKKTRKV-WAGKFFKAYS-------------AKEKENIRQEISIM---NCL-H--------H-PKLVQCVDAF--**

**Hs_MYLK2(NP_149109.1)**  **-M-NSK--EAL-GGGKFGAVCT-CMEKATGLK-LAAKVIKKQT-------------PKDKEMVLLEIEVM---NQL-N--------H-RNLIQLYAAI--**

**Hs_MYLK3(NP_872299.2)**  **-V-CQH--EVL-GGGRFGQVHR-CTEKSTGLP-LAAKIIKVKS-------------AKDREDVKNEINIM---NQL-S--------H-VNLIQLYDAF--**

**Hs_MYLK4(NP_001012418.2)**  **-V-SKT--EIL-GGGRFGQVHK-CEETATGLK-LAAKIIKTRG-------------MKDKEEVKNEISVM---NQL-D--------H-ANLIQLYDAF--**

**Hs_OBSCN_A(NP_001373054.1)**  **-Y-EVK--EEI-GRGVFGFVKR-VQHKGNKIL-CAAKFIPLRS--------------RTRAQAYRERDIL---AAL-S--------H-PLVTGLLDQF--**

**Hs_OBSCN_B(NP_001373054.1)**  **-F-AFQ--TQI-QRGRFSVVRQ-CWEKASGRA-LAAKIIPYHP--------------KDKTAVLREYEAL---KGL-R--------H-PHLAQLHAAY--**

**Hs_SPEG_A(NP_005867.3)**  **-Y-DIH--QEI-GRGAFSYLRR-IVERSSGLE-FAAKFIPSQA--------------KPKASARREARLL---ARL-Q--------H-DCVLYFHEAF--**

**Hs_SPEG_B(NP_005867.3)**  **-Y-TFL--EEK-ARGRFGVVRA-CRENATGRT-FVAKIVPYAA--------------EGKRRVLQEYEVL---RTL-H--------H-ERIMSLHEAY--**

**Hs_ST17A(NP_004751.2)**  **-Y-SLCPGREL-GRGKFAVVRK-CIKKDSGKE-FAAKFMRKRR-----------KGQDCRMEIIHEIAVL---ELAQD--------N-PWVINLHEVY--**

**Hs_ST17B(NP_004217.1)**  **-Y-ILTS-KEL-GRGKFAVVRQ-CISKSTGQE-YAAKFLKKRR-----------RGQDCRAEILHEIAVL---ELAKS--------C-PRVINLHEVY--**

**Hst_TRIO(GKDX01093008.1)**  **-Y-ELM--DEI-GRGRYSTVYE-CLHRTTQQP-YAAKLLPVEV---------------GKEKAQQELQLL---SRL-T--------H-RNIVQMTSAY--**

**Hst_TRIO_like_A(GKDX01095742.1** **-Y-DILP-DEI-GRGRFGIVHK-CATKFSGLQ-LAAKYVRLRS--------------RKKPETRREVEIL---LKLRR--------KSPHILEFYDAF--**

**Hst_TRIO_like_B(GKDX01095742.1** **-Y-TLG--PEI-AKGVYAQVRK-CTENSTDRV-FAAKMILSNT-------------MEYQALGVYEYEMH---RQL-V--------H-PRIVALEDAF--**

**Hs_TRIO(NP_009049.2)**  **-Y-SEV--AEL-GRGRFSVVKK-CDQKGTKRA-VATKFVNKKL--------------MKRDQVTHELGIL---QSL-Q--------H-PLLVGLLDTF--**

**Hv_CON_A(XP_047137784.1)**  **-Y-VLK--EEL-GRGKFGVVNK-CVDKFSKIE-YAAKFLKYRP--------------SERSNILNEIDIM---NSL-N--------H-KRLINLVAAF--**

**Hv_CON_B(XP_047137784.1)**  **-Y-NFK--DEI-GRGKFAVVKV-CASKATGDT-YAAKLVKYDE--------------DTMEVTKKEYEIW---RSL-N--------H-PKLVLLRDAY--**

**Hv_DAPK1(XP_012558089.1)**  **-Y-DVM--QEL-GRGQFAVVKK-CISKENNQE-VAAKFIKVKR-------SKASKNGLSKELIEREAGIL---FSV-D--------H-SKIIKLYDLF--**

**Hv_DAPK2(XP_047139830.1)**  **-Y-EVK--EEL-GRGHFAVVKK-CVSKENNRE-VAAKFIKLKR-------SKASKIGMSKELIERESNIL---FAI-D--------H-AKIIKLYDIF--**

**Hv_TRIO(XP_047137671.1)**  **-Y-QIC--GEL-GRGRFSVVKR-CVRKNTKEE-FAAKIFRRRL--------------ISKETVESEVAVM---QSL-N--------H-PGVVKVFEIY--**

**Ml_TRIO_like(GFAT01019191.1)**  **-Y-KFT--DIL-FYGHFSMFCT-CEHQRTGLK-FTAQVTPVVD-------------RQFYRSIQREVEIL---QQL-N--------Y-KRVSRLHEVL--**

**Nv_CON_A1(XP_048579265.1)**  **-Y-DSL--EEL-GKGKFGVVKK-VKDKKTGEV-LAAKFIRTSS--------------ESKKEVMGEIAMM---NHL-H--------S-KRLIYLADAY--**

**Nv_CON_A2(XP_048579265.1)**  **-Y-TIK--DEL-GKGRFGVVCK-CVNKKTGKQ-FAAKFIKCSK-------------PQDREDVIHEMEIM---NTI-R--------H-KRLLRLADAF--**

**Nv_CON_B(XP_048579265.1)**  **-Y-SFK--EEV-GRGKFAVVKG-CQDVTTQKD-FVAKLIRYDE--------------EDREDAVQEFSVH---RGI-D--------C-NQVVRLHNAF--**

**Nv_DAPK(XP_032239611.2)**  **-F-DIG--DEI-GSGQFAVVKK-CSEKSSGLE-FAAKFMKKRR-------SKALRRGVTLEQIIREATVL---RSV-A--------H-QGIIYLHDIY--**

**Nv_TRIO(XP_032241348.2)**  **-Y-NIY--AEL-GRGRYAVVKK-CVEKSTGKE-FAAKMVKKRM--------------LDPVDIDREVTVL---RML-K--------H-PNLCIFLDAY--**

**Nv_STK17(XP_001629087.2)**  **-Y-DIH--DQI-GRGQYAVVRR-VTHKTTGLE-YAAKFVRKRR-----------KGQDCRSEVWHEVEVLWSTNHPYQ--------H-TKIIQLHEVY--**

**Om_TRIO_like_A(KAI6652169.1)**  **-Y-TYS--KEI-GRGRFAVVYT-CCFKGAKFD-LAAKIERVTT--------------DVRKPLQHEASIL---TGL-K--------H-PNIVELIEFE--**

**Om_TRIO_like_B(KAI6652169.1)**  **-F-ILC--GEL-TAGRSADIKL-CKEKLTGHK-FVAKFIPITS--------------RSQEDFKRETDIH---KEL-I--------H-PRVVQYNSRF--**

**Om_STK17(KAI6649438.1)**  **-Y-VVG--DVL-GRGKFATVKH-ITEKATGNC-YAAKYLRKRN-----------YAVNCRNTILQEAELL---QELLE--------H-RVIVDVKEVF--**

**Os_CON(XP_036371388.1)**  **-Y-IIY--EEL-GRGAFGVVHR-AVERATGKV-WAAKFIKCRP--------------HEKEAVEHEIYIM---NCL-H--------H-RKLLQLHEAF--**

**Os_DAPK(XP_029643431.1)**  **-Y-TIG--ENL-GSGQFAVVKK-CKQRETGKE-YAAKYIRKRR--------AGGRRGASLEDIRKEVEIL---CEL-D--------H-PNIVQLYECF--**

**Os_TRIO(XP_029657616.1)**  **-Y-NEL--EEL-GRGRFAVVKR-CVQKCSGQE-LAAKCINRKH--------------LSKEAIETEFNTL---QSL-F--------H-THLVKVFDLY--**

**Os_MYLK(XP_029655504.1)**  **-Y-ILK--EEV-GSGRFGVVHK-CIEKSSNRL-WAAKVIKCKE--------------KKKAEFRNEIEIM---KNL-T--------H-PKVLRLWDAY--**

**Os_OBSCN_A(XP_036361934.1)**  **-Y--IL--EEI-GRGRHGIIRR-VIEKSTGKE-FAGKFLTLSD-------------PKEKEFFQTELESL---RVL-D--------H-PHIMKIHEAY--**

**Os_OBSCN_B(XP_036361934.1)**  **-Y-TFL--EEI-TRGAFRQWKL-CRSKRTEEL-YYARITPYET---------------PTDEIMNEFNLL---VTV-Q--------H-VNVITVIGAF--**

**Os_STK17(XP_029636789.1)**  **-Y-SIQ--GEL-GRGKFAVVKR-CVNLETNEE-VAAKFIRKRR-----------RGKNCRDQILREIVML---ELSRP--------H-HRVVDLIEVF--**

**Os_twitchin(XP_036360273.1)**  **-Y-DIL--EEL-GSGAFGVVHR-CIEKSTGRV-FVAKFINTPY-------------PLDKVTVRNEINVM---NHL-H--------H-PQLMNLHDAF--**

**Pc_DAPK(XP_045596115.1)**  **-Y-EVY--EEI-GSGQFAVVRR-CVEKATRAE-YAAKYIRKRR-------VASSRRGLPLECIAREVRVL---QKLDN--------H-QNIISLHQVF--**

**Pc_MYLK(XP_045598454.1)**  **-F-DLK--KEI-GRGRFGTVYL-AEDKATGQK-FAAKFVNTKR-------------NQDRANAVREVAIM---KSL-N----SESPH-PRLIQLYDAY--**

**Pc_OBSCN(XP_045586627.1)**  **-Y-DVG--DEI-GRGTQGVVYH-CVEHHTGRN-FAAKSMWGKD--------------NFKTWMRLEYEMM---NLNNS--------C-KQILRLYDAY--**

**Pc_projectin(XP_045599281.1+BA** **-Y-DIL--EEI-GTGAFGVVHR-CRERKTGNI-FAAKFIPVAS-------------AMEKELIRKEIDIM---NHL-H--------H-PKLINLHDAF--**

**Pc_STK17(XP_045598575.1)**  **-Y-DVE--QTPFARGKFAAVRR-ARCLRTGTW-FAAKVMRKRR-----------RAQDVRHEILHEAAVL---LLARP--------S-SRIVSLHQLY--**

**Pc_stretchinMLCK(XP_045616175.** **-Y-NVH--EEV-GKGRFGVVYR-VTDKITGVR-RAAKIIRCIK-------------AKEKEKVREEIDIM---NSL-R--------H-PKLLQLGAAY--**

**Sk_CON(XP_006823011.1)**  **-Y-EIC--EEL-GRGAYGVVYR-AIEKTTTKT-WAAKFITVGE--------------DERKAVKKEIEIM---CQL-H--------H-KRLLQLHEVF--**

**Sk_DAPK1(XP_006823401.1)**  **-Y-DIG--EEI-GSGQFATVKR-VTNKTTAIE-YAGKFVKKKK-------MASSRRGAKKEDIVREVEIL---SEM-K--------H-RNVISLHEVY--**

**Sk_TRIO(XP_006819276.1)**  **-F-SEM--AEI-GRGRFSFVRK-CVQLTTGRD-VAAKLISKKL--------------TTVDRVENEIAIL---RSL-Q--------H-PHICEYYDAF--**

**Sk_MYLK(XP_006823003.1)**  **-Y-QIK--EEL-GRGKFGTVNK-CVEKKTKKI-LAAKFIKVNS-------------KADRDEVENEISIM---QIL-Q--------H-PKLLQLYDAF--**

**Sk_OBSCN_A(XP_006823009.1)**  **-Y-EVR--EEL-GRGAYGVVKH-AVSRKDGRD-CAAKFIRSKP--------------TMRREFRQEMDIM---SSL-D--------H-PRLIKLMDGY--**

**Sk_OBSCN_B(XP_006823009.1)**  **-Y-NFE--DEI-GRGRFGVVRK-CVEHNTNIE-YAAKLLRVKP--------------NNESQLLEEYELL---KDL-R--------Y-PRIGMLHDAY--**

**Sk_STK17(XP_002741442.1)**  **-Y-TVG--CEL-GRGKYSVVKK-CTENFTGKE-FAAKFLKLRK-----------RGKDCRNEILHEIAIL---EISKN--------N-PRLISLHEVY--**

**Sk_twitchin(XP_006825350.1)**  **-Y-DIG--EEL-GRGDFGVVYR-AVERSTQRN-FAAKFIDCKS-------------PIEKAAIKAEIKMM---NSL-Q--------Y-PKLLQLHDAY--**

**Sp_CON(XP_030830392.1)**  **-Y-QIL--EEL-GRGSYGIVYR-AIEKKTQKT-WAAKFMRCFG--------------KERDLVRREIEVM---KKL-H--------H-RRLLNLHEVF--**

**Sp_DAPK(XP_030832558.1)**  **-Y-QIG--EDI-GSGQFSEVKK-VTEKSTGKD-YAGKFIRKRR-------STASRRGVKREDIVREVSIL---EEL-S--------H-DNIISLHDAF--**

**Sp_TRIO(XP_011680751.1)**  **-F-NIT--REI-GRGRFSSTHA-CTRSQQQRT-LAAKFISKRL--------------MDLEQVQHEASMM---QPL-Q--------H-PLICSLHATY--**

**Sp_MYLK_A1(XP_030830851.1)**  **-Y-IVK--EEL-GKGKFGIVYR-CEEKSTGKT-WAAKYVKTIR-------------AKDKEAVQREIDLM---SEL-E--------H-PSLMALIEAY--**

**Sp_MYLK_A2(XP_030830851.1)**  **-Y-QIK--EVL-GKGRFGTVHK-CIEKVTGKA-YAAKMIKTIK-------------STDKESVKNEIEIM---NKL-H--------H-AKLLQCLDAF--**

**Sp_OBSCN_A(XP_030830075.1)**  **-Y-ELL--TEL-GRGAYGVVHK-GISKADGSE-CAVKTIRVKA--------------GMWEEVRNEIAVM---GIL-D--------H-KRLIKLFDAY--**

**Sp_OBSCN_B(XP_030830075.1)**  **-Y-EMQ--EEI-GRGRYSVVMR-CRKVATCKE-YAVKILAHTK--------------KTQDKCLAEYELL---KEL-S--------H-PHILQLREAF--**

**Sp_STK17(XP_030846519.1)**  **-Y-STL--NEL-GRGRFAVVRK-CKHKESNRH-FAAKFVRKRK-----------MGRDCREDILKEIRIL--ENSVLN----------QRLIGLHEVY--**

**Sp_twitchin(XP_030829654.1)**  **-Y-HIG--EEI-GRGPFGVVHR-CIERGTGRT-FAAKFMDVEP--------------KDKAFIKEEIEAM---NQL-Q--------H-PRLQQCHDAF--**

**Ta_CON_A1(GHJI01005353.1)**  **-Y-TLL--EEL-GKGRFGVVYR-CADE-SGNI-FAAKHIDLKN--------------SKEEDIHREIDVM---NCL-D--------H-ERLVRLYAVY--**

**Ta_CON_A2(GHJI01005353.1)**  **-Y-VMK--DEL-GRGKFGIVRA-CTEKSTGVE-YAAKMIKTKV--------------SDRTTVLQEIDIM---NQL-H--------H-PKLVFLHDAY--**

**Ta_TRIO(GHJI01000868.1)**  **-Y-NLY--DEI-GKGKFADVKH-CTHRETLKE-FAVKIYQKDI--------------ISKAGVQRELDIW---QPL-Q--------H-AKICSFHEVY--**

110 120 130 140 150 160 170 180 190 200

....|....|....|....|....|....|....|....|....|....|....|....|....|....|....|....|....|....|....|....|

**Aa_CON_A(GHAI01170451.1)**  **ETS-K--NIVLVLELVT----GGELF-EKLT---------------------ALEFISEHVVVYYMKQVLQGLSYMH-ERQILHLDLKPENIML-VNPN-**

**Aa_CON_B(GHAI01170451.1)**  **LVR-K--YLVLISEMIN----GVPIL-DHLG---------------------RMANPTEDDVAAFTVQLLEALEYLQ-MRNVVHLDIKPGNLLI-KGNV-**

**Aa_TRIO(GHAI01159172.1)**  **DTP-K--NLILVLELLS----GGRLF-DHIV---------------------IMDNLTEKIAIGFVKQILQALQYLH-ECGIAHLDVKPENLILSHGTT-**

**Am_CON_A1(XP_044172413.1)**  **ERP-K--EMIIVLELIS----GGELF-EKVC---------------------SDDDLTEKEVVRYMKQILQGVEHMH-KKRIVHLDLKPENVLCVIRPD-**

**Am_CON_A2(XP_044172413.1)**  **QTP-T--EMILVMELIS----GGELF-EKVV---------------------EDEFISETEVSYYMKQILEGIQHMH-QHDVLHLDLKPENIML-IRPD-**

**Am_CON_B(XP_029213098.1)**  **LMR-R--YLVLVMDLLE----GQDIL-SFIA---------------------AKPRPNEEDIAFTIRPLLDVVNYLH-GQKIVHLDVRPANIFI-AKSN-**

**Am_DAPK(XP_029207283.2)**  **ETT-V--DYTLVLELVS----GGELF-EFLS---------------------EQEYLTESEALGFTKQVVEAIHYLH-DNHIVHLDIKPENIVL-KDRE-**

**Am_TRIO(XP_029203193.2)**  **DTP-K--NYVLIIELLA----GGRLF-DHLV---------------------VMDNLTEKVAISYVREILEGVQHLR-DLSIVHLDLKPQNLLLAAGPL-**

**Aq_TRIO(XP_019864448.1)**  **LSH-Q--HFIIVMQYIQ----GLSPL-TYFN----------------------ERNSSEHDIAKFCRELCCGLKHLH-DHGIVHLAIKPDSVYV-VPNSF**

**Aq_TRIO_like_A(XP_019854769.1)** **ITD-E--NIVIITEVAD----GGQLI-DYIL--------------------RMYNSLNELLIVEYVKQLLSALDYIH-ERQIVHLDVKPENLLI-ASEV-**

**Aq_TRIO_like_B(XP_019854769.1)** **RSE-N--HVILVLQYVP----GGDLF-NYIT---------------------SSGPIDERQSARYIRHILEGLEYLH-NRNILLINIAPENLVLGVGLQ-**

**Aq_STK17(XP_003383221.1)**  **ESP-R--EMIIIIELAT----GGELF--RMI---------------------AVDPLPEDKARGVVLQLLEGVEHLH-SLSIVHLDLKPENILLYRKGQ-**

**Bf_CON(XP_035661587.1)**  **EAH-D--QLTMIREFIS----GRELL-EMVT--------------------DHRVHYTEADCIHYTRQICEGLEFLH-SKNILHLHLRPESIMCCTHVG-**

**Bf_DAPK(XP_035665897.1)**  **EDK-Q--DVTLILELVS----GGELF-DFIA---------------------ERDVLHESEATAFIAQVLEGLAHMH-LKNIAHLDLKPENILL-TNRA-**

**Bf_TRIO(XP_035662010.1)**  **DVA-Q--SLIIILELIP----HGRLL-DWIV-------------------LNHRGNYTEQHVVGYVVQVMEAVQYLH-NCRVAHLDIKPENIMVDGDSL-**

**Bf_MYLK(XP_035660911.1)**  **RQR-N--QLTLVLELVS----GGELF-ERVI--------------------DDDFVLTEKDCVIFTRQICQGVGFMH-DQGVLHLDLKPENVLC-VNRT-**

**Bf_MYLK2(XP_035661607.1)**  **DCG-K--EVAMIMELIT----GGELF-QLVI--------------------DEAVELSEKACVSYVRQLCEAVSYMH-EQNILHLDLKPENIMC-ISKK-**

**Bf_OBSCN_A(XP_035660917.1)**  **LET-K--FVVMVMEYVA----MGELL-EHLV---------------------KVPDLSEADIVPYLSQLLGALQYIH-SKDIVHLDIKPENLLL-TKSD-**

**Bf_OBSCN_B(GESZ01107032.1+XP_0** **ITS-K--YLILVCEKYY----GGDVL-RYLT---------------------TKPHYSEEEVAQFITQVLEALHYLH-TQKIVHLDVKPDNLLLESRRR-**

**Bfo_TRIO_like(GHXY01112106.1)**  **VDYEG--CVVILMSYQY----KLPLL-DFLL---------------------LKESHKEHESIDMIKSLLEGVWYLH-SRCIAHLDLKPESIIW-DKTT-**

**Bf_STK17(XM_035832943.1)**  **ETH-A--EMILVLEYAA----GGEIF-DHCV-------------------TEEDDCFKEKDVVRLMRQILEGVSYLH-ERNVIHLDLKPQNILL-TKPVP**

**Bm_TRIO_like(GKLW01050522.1)**  **LQYDN--CIVLIMSYQF----QLPLM-DFLL---------------------LKESNKEHETIEIIKNVLEGVSYLH-ARSIAHLDLKPENLIW-DKTT-**

**Ce_DAPK(NP_490840.2)**  **ETA-S--DVIIVLELVS----GGELF-DHVC---------------------AKECLDEVEAAAFIKQILLAVRHLH-SLHIVHLDIKPENVML-KQRG-**

**Ce_MYLK(NP_509689.1)**  **YTT-T-NDVVLIMEIVR----GGELF-DRVA--------------------EESYVLSELAVVMIICQLCEAIDYIH-KQNILHLDVKPENIMC-VSLT-**

**Ce_OBSCN_A(NP_001343714.1)**  **RDE-K--LALVVFDNANSTIDGLSSL-AHPG----------------VEIAEPKGVNRETCVRVFVRQLLLALKHMH-DLRIAHLDLRPETILL------**

**Ce_OBSCN_B(NP_001343714.1)**  **NNS-N--FLYLLSERL-----YEDVF-SRFV---------------------FNDYYTEEQVALTMRQVTSALHFLH-FKGIAHLDVNPHNIMFQSKRS-**

**Ce_titin(G4SLH0.2)**  **DMG-N--EMWLIEEFVS----GGELF-EKIL--------------------EDDSLMSEEEVRDYMHQILLGVSHMH-KNQIVHLDLKPENILLKAKNS-**

**Ce_twitchin(NP_502274.2)**  **EDD-N--EMVMIYEFMS----GGELF-EKVA--------------------DEHNKMSEDEAVEYMRQVCKGLCHMH-ENNYVHLDLKPENIMFTTKRS-**

**Cg_CON_A1(XP_034304307.1)**  **DQA-G--EMVMILELLT----GGELF-DRLV--------------------EQEYDLTEEDCITYMRQICQGVRHMH-QQNLVHLDLKPENVMCVTKES-**

**Cg_CON_A2(XP_034304307.1)**  **ETA-R--NVVLVMEYVA----GGELF-DRVA--------------------DEDLELTESDCVHFMRQICQGLQYMH-LRSILHLDLKPENILC-INKD-**

**Cg_TRIO(XP_034303731.1)**  **ETA-S--NLLIVMEFLE----VGRLF-EFIC---------------------QRQIFDEIEAADYIRQLLTALQYLH-NCRIVHLDVKPENLMVQNVMG-**

**Cg_MYLK(XP_011441364.2)**  **EKK-D--TFCIVTELIS----GGELF-ERVI--------------------NDDFILTEKACIMFMRQICEGIAFMH-CREVLHLDLKPENILC-LTRE-**

**Cg_OBSCN_A(XP_034299497.1)**  **VTE-R--RIVIITEIVT----DYDII-DSLL---------------------QSPTPTESMVAAHIKQLLLVIKELH-KAAVLHLDIKPSNIRFGTNND-**

**Cg_OBSCN_B(XP_034299497.1)**  **FYL-D--QYYWILEYLS----GVNVV-EHFS---------------------YKSKYTEDMVAIVIRQVLDGLQFLH-YHGYAHLNIQPSSIMMVNRRR-**

**Cg_STK17(XP_011422577.1)**  **ETP-N--ELVLITEYCA----GGELF-TECV---------------------IEESFTESDVIRFLIQILEGLAYLH-ERNIVHLDLKPQNILF-TKPFP**

**Cg_twitchin(XP_034332726.1)**  **EDR-H--EMILVLEFLS----GGELF-DRIA--------------------AEDYKMTEAEVINYLRQVCEGLKHMH-EHSIVHLDVKPENVMCETKKS-**

**Ct_CON(ELT96026.1+AMQN01011565** **ETPEG--HIIFIMELLS----GGDIL-QYIS--------------------SKDTSYTELHCINLMRQVCQAVSHIH-KHNIIHMEIRPENIMFQTSHS-**

**Ct_DAPK(ELT88671.1)**  **ENK-S--EVILVLELVC----GGELF-QFLA---------------------EREKVNEDEAVEFLKQILEGVRHLH-EHSIVHLDLKPENLML-LGQN-**

**Ct_TRIO(ELU10047.1)**  **ESP-T--NFIIILSYIS----GPPIF-DFLC---------------------TKPTFNECEASSYMYQLLNALQYIH-SYNVAHLDIKPENILFDTTTS-**

**Ct_MYLK(AMQN01000602.1+ELU1638** **ESK-T--EMCLILEIIY----GGELF-DRVI--------------------SEDFLLTEKACQCFIRQICEGLEYMH-TCSIIHLDMKPENILC-ISQT-**

**Ct_MYLK2(ELT87547.1)**  **ETR-R--EMVLIMEHVA----GGELF-ERII--------------------DEDYILTERESIHFMRQIVSGVHYMH-ENNILHLDLKPENILC-ISKN-**

**Ct_OBSCN(ELU05514.1)**  **EDK-K--RLVIVSEIVT----GGELF-NRLQ---------------------KEDSLTESEVAFYMRQLLLAVEHMH-AKNVVHLDLKPENLFLLSPSS-**

**Ct_STK17(ELU16209.1)**  **QAP-S--EIIIVTEYVS----GGELL-RHVV---------------------WDEMIEEPLAARIVRQTLHALAYLH-THNIVHMDVKPQNILL-TRSLP**

**Ct_twitchin(ELT89874.1)**  **DDK-Q--EMVLVLEYLS----GGELF-DRIA--------------------DEDYKMTEAEVINYIKQVCDGLAHMH-ENNIVHLDVKPENVMCETSKS-**

**Dj_DAPK1(IAAB01043597.1)**  **DKK-D--MSILILELVT----GGELF-EYIS---------------------NKVTLSEEEASKFIRQILHGVHHLH-SKNILHLDLKPENILV-EDPN-**

**Dj_DAPK2(IAAB01051648.1)**  **NLG-K--TVALVLELVS----GGELF-DHIS---------------------DSEKLSEEEASEFIEQILYGVQHMH-DRKVVHLDLKPENVMI-EDLD-**

**Dj_MYLK(IAAB01042781.1)**  **EWR-D--EIFMILELIQ----GGELF-ERIV--------------------DEKFDLTEDKCEEFMIEICQGVEYIH-SQKILHLDLKPENILC-LHRT-**

**Dj_STK17(IAAB01043480.1)**  **ESY-I--EVALVMEIAK----GGCLF-DVVR--------------------ESQNGLPENYVQQIIKRLLSALLAMH-KTNIVHLDIKADNILL-REPYP**

**Dj_twitchin(IAAB01071515.1)**  **DDK-D--EMCLIMEFLS----GGELF-DRIT--------------------EDGYNMNEAEAANYIRQVCEGLKFMH-DEGYIHLDLKPENIMCETSNS-**

**Dm_MYLK(NP_001260832.1)**  **EYQ-K--MMCVVLELIE----GGELF-DRVV--------------------DDEFVLTERVCRVFIRQVCEAMAFIH-GNGIVHLDLKPENILV-LTQK-**

**Dm_OBSCN_A(XP_034109413.1)**  **DTD-R--SVTLIMELAA----GGELVRDNLL---------------------RRDYYTERDIAHYIRQTLWGLEHMH-EMGVGHMGLTIKDLLISVVGG-**

**Dm_OBSCN_B(XP_034109413.1)**  **LNV-P--IAIFVMEKLQ----GADVL-TYFS---------------------SRHEYSEQMVATVVTQLLDALQYLH-WRGYCHLNIQPDNVVMASVRS-**

**Dm_projectin(NP_995598.1)**  **EDD-D--EMILILEFLS----GGELF-ERIT--------------------AEGYVMTEAEVINYMRQICEGIRHMH-EQNIIHLDIKPENIMCQTRSS-**

**Dm_STK17(NP_001162723.1)**  **ETR-S--DTALLLELAT----GGELQ-TILD---------------------NEECLTEAQARHCMREVLKALKFLH-DRSIAHLDLKPQNILL-AGERI**

**Dm_stretchinMLCK(NP_725510)**  **ESP-R--EIVMVMEYIT----GGELF-ERVV--------------------ADDFTLTEMDCILFLRQVCDGVAYMH-GQSVVHLDLKPENIMC-HTRT-**

**Ef_CON(GIUK01093179.1)**  **ETQ-G--EMAFILEMVS----GRDLL-SGLV--------------------DCHKVRTEADCINWMRQVCQGVSHMH-KNNIIHLDLRPENIMFTTSKSD**

**Ef_DAPK(GIUK01016195.1)**  **EDK-Q--DVVLVLELVS----GGELF-DYLL---------------------ERDKVGEAEAIVILLQILEGTKHLH-DRNILHMDLKPENLML-KSQD-**

**Ef_DAPK(GIUK01061946.1)**  **EGC-L--DIILILELVS----GGELF-NYLA---------------------EKDKVSEDEAVVFLKQILEGVQHLH-SLNIIHLDLKPENLVL-DGND-**

**Ef_MYLK2(GIUK01050934.1)**  **ESA-K--EIILVMEHVS----GGELM-ERII--------------------NDDYVLTERECIHFMRQICDGVQYMH-SSDILHLDLKPENILC-ITPN-**

**Ef_MYLK(GIUK01094627.1)**  **EAK-K--EMCLVLEIIY----GGELF-DRVV--------------------SDDFLLTEKACVCLVRQICEGVEFMH-SKNIIHLDLKPENILC-LTKS-**

**Ef_MYLK(GIUK01083042.1)**  **ESR-S--QMCIVLEIVN----GGELF-DRVI--------------------TDEFLLTEKACVCIVRQICEGIEYMH-SKNTIHLDLKPENIMC-LTKT-**

**Ef_OBSCN_A(GIUK01083331.1)**  **EDK-K--RLAIVMEIVT----GGELL-AKLV---------------------AEDAFRESEAAFYLRQLLLAVEYMH-SKNVLHLDLRPENLLLASPSS-**

**Ef_OBSCN_B(GIUK01083331.1)**  **LSD-D--FVYLVFEKLY----GENCV-RSIS---------------------LKNKYDEYLVTHIIQQVLDALQFLH-HRGIVHLNIKPDNVVMNSRRR-**

**Ef_STK17(GIUK01063378.1)**  **ETN-L--DLIIVTDFAK----GGELF-EHIM---------------------AEGQLEESVTIRLLRQILDGLSFLH-ANNIAHLDVKPQNILL-TDALP**

**Ef_twitchin(GIUK01013637.1)**  **DDQ-H--EMVMVFEYLS----GGELF-DQIA--------------------DEDNKMTEGDVVRYVRQVCDGISHMH-DQNIVHLDIKPENILCVNKKS-**

**Ef_twitchin(GIUK01097374.1)**  **EDQ-H--EMVMVLEYLS----GGELF-DQVA--------------------DENYRMTEAEVIHYMRQICEGLQHMH-DQNIVHLDVKPENVMCTTSKS-**

**Eg_DAPK(KAH9282855.1)**  **EHE-D--YSIILLELVN----GGELF-ARVA---------------------ELERLDEQEAVFFIAQILLGVNHMH-QLGIVHLDLKPENIMI-EDME-**

**Eg_MYLK(XP_024351289.1)**  **IQR-D--RITLIMELIT----GGELF-ERVI--------------------DDSFDLNEEICEKFMRQILQGVEYIH-SQHVIHLDLKPENILC-LSRT-**

**Eg_STK17(KAH9283880.1)**  **EDP-T--QVAIVMEFAE----GGSLF-DLCSPKTFNRPPLPSGDGDFSDGTTPQSRIPESYVSSVLSRITNALAFMHEKANIVHLDIKAENILL-RKPYP**

**Eg_twitchin(KAH9283145.1)**  **SEP-N--ETALVLEFLS----GGELF-DRIA--------------------DDGYTMNEAEVIKYIRQLLEGLQHMH-ENHIVHLDIKPENIMCETSRS-**

**Hc_TRIO_like(GHXS01109180.1)**  **TDYKD--CVCLIMSYQY----QLPLL-DFVL---------------------LKESHRELDTVEMMRALLEGVWYLH-TRNIAHLDIKPENLIW-DKTT-**

**Hs_CON(NP_003310.4)**  **ESM-E--ELVMIFEFIS----GLDIF-ERIN--------------------TSAFELNEREIVSYVHQVCEALQFLH-SHNIGHFDIRPENIIYQTRRS-**

**Hs_DAPK1(NP_004929.2)**  **ENK-T--DVILILELVA----GGELF-DFLA---------------------EKESLTEEEATEFLKQILNGVYYLH-SLQIAHFDLKPENIML-LDRNV**

**Hs_DAPK2(NP_055141.2)**  **ENR-T--DVVLILELVS----GGELF-DFLA---------------------QKESLSEEEATSFIKQILDGVNYLH-TKKIAHFDLKPENIML-LDKNI**

**Hs_DAPK3(NP_001339.1)**  **ENK-T--DVVLILELVS----GGELF-DFLA---------------------EKESLTEDEATQFLKQILDGVHYLH-SKRIAHFDLKPENIML-LDKNV**

**Hs_KALRN(NP_001375348.1)**  **ESP-T--SYILILELMD----DGRLL-DYLM---------------------NHDELMEEKVAFYIRDIMEALQYLH-NCRVAHLDIKPENLLIDLRIP-**

**Hs_MYLK(NP_444253.3)**  **EEK-A--NIVMVLEIVS----GGELF-ERII--------------------DEDFELTERECIKYMRQISEGVEYIH-KQGIVHLDLKPENIMC-VNKT-**

**Hs_MYLK2(NP_149109.1)**  **ETP-H--EIVLFMEYIE----GGELF-ERIV--------------------DEDYHLTEVDTMVFVRQICDGILFMH-KMRVLHLDLKPENILC-VNTT-**

**Hs_MYLK3(NP_872299.2)**  **ESK-H--SCTLVMEYVD----GGELF-DRIT--------------------DEKYHLTELDVVLFTRQICEGVHYLH-QHYILHLDLKPENILC-VNQT-**

**Hs_MYLK4(NP_001012418.2)**  **ESK-N--DIVLVMEYVD----GGELF-DRII--------------------DESYNLTELDTILFMKQICEGIRHMH-QMYILHLDLKPENILC-VNRD-**

**Hs_OBSCN_A(NP_001373054.1)**  **ETR-K--TLILILELCS----SEELL-DRLY---------------------RKGVVTEAEVKVYIQQLVEGLHYLH-SHGVLHLDIKPSNILM-VHPA-**

**Hs_OBSCN_B(NP_001373054.1)**  **LSP-R--HLVLILELCS----GPELL-PCLA---------------------ERASYSESEVKDYLWQMLSATQYLH-NQHILHLDLRSENMIITEYNL-**

**Hs_SPEG_A(NP_005867.3)**  **ERR-R--GLVIVTELC-----TEELL-ERIA---------------------RKPTVCESEIRAYMRQVLEGIHYLH-QSHVLHLDVKPENLLVWDGAA-**

**Hs_SPEG_B(NP_005867.3)**  **ITP-R--YLVLIAESCG----NRELL-CGLS---------------------DRFRYSEDDVATYMVQLLQGLDYLH-GHHVLHLDIKPDNLLLAPDNA-**

**Hs_ST17A(NP_004751.2)**  **ETA-S--EMILVLEYAA----GGEIF-DQCV-------------------ADREEAFKEKDVQRLMRQILEGVHFLH-TRDVVHLDLKPQNILL-TSESP**

**Hs_ST17B(NP_004217.1)**  **ENT-S--EIILILEYAA----GGEIF-SLCL-------------------PELAEMVSENDVIRLIKQILEGVYYLH-QNNIVHLDLKPQNILL-SSIYP**

**Hst_TRIO(GKDX01093008.1)**  **LTD-K--HFIMLFHMLQ----GVNLF-EYFT---------------------ASDSLTESSAAACIQQITSGLRYLH-SNDIAHLDIKPENIMAVEREG-**

**Hst_TRIO_like_A(GKDX01095742.1** **EMG-R--NLIIVTELVP----DGELI-ERII---------------------ADNALTEAVAVRYLSQLLGALECLH-LLSVAHLDIKPENILL-FGRE-**

**Hst_TRIO_like_B(GKDX01095742.1** **QTD-T--HTILVLEYMP----GGTLQ-SLVS---------------------RYESLPEEEVSRFVRKIVEGLQYLH-DMKIIHVFLRPDNILLTADQN-**

**Hs_TRIO(NP_009049.2)**  **ETP-T--SYILVLEMAD----QGRLL-DCVV---------------------RWGSLTEGKIRAHLGEVLEAVRYLH-NCRIAHLDLKPENILVDESLA-**

**Hv_CON_A(XP_047137784.1)**  **EQP-K--QIVLVLELVT----GGELF-EKLT---------------------EEEYISEKDVTFYMKQVLQGVQHMH-ENNILHLDLKPENIML-VNPR-**

**Hv_CON_B(XP_047137784.1)**  **IVR-K--YLILICDLVN----GKHVL-NYLI---------------------DLKAVNENIIANCINELLEALQYLH-SQDVCHLDIKPGNMMM-VGSK-**

**Hv_DAPK1(XP_012558089.1)**  **DIG-T--EIVLVLELLS----GGELF-DKIC---------------------ECEFLKEVDACFYMKQVLEAVYHIH-SLNIVHLDIKPENIVL-QSKN-**

**Hv_DAPK2(XP_047139830.1)**  **DIG-S--EMVLVLELLT----GGELF-DKIC---------------------ESEYMKESDACSYMIQVLEAVQHIH-SFNIVHLDIKPENIVL-QSKN-**

**Hv_TRIO(XP_047137671.1)**  **EAP-K--SLIIIQQMLS----GGRLF-DHIV---------------------VMDMLTENLAKLYIIQLLRAVQYIH-SLSIAHLDIKPENILLSDGPE-**

**Ml_TRIO_like(GFAT01019191.1)**  **LQYDN--CIVLVMSYQY----QLPLM-DFLL---------------------LKETHKEHETIEIIKNILEGLCYLH-ARSIAHLDLKPENFIW-DKTT-**

**Nv_CON_A1(XP_048579265.1)**  **ERP-G--EMIVIMEFVS----GGELF-EKIC---------------------NDDNLTEKEVIRYMRQILQGVEHMH-RKSIVHLDLKPENVLCVIRPD-**

**Nv_CON_A2(XP_048579265.1)**  **ETP-SQQEMILVMELVT----GGELF-EKVV---------------------EEEFISENDVTHYMKQILEGLEHMH-KQEVLHLDLKPENIMI-VKQD-**

**Nv_CON_B(XP_048579265.1)**  **LLR-S--YLVLIMDRLD----GEDVL-KFMS---------------------SKTKVTEEDAALVIRGILNALCYLH-ELNIVHLDIRPANIMV-QGSD-**

**Nv_DAPK(XP_032239611.2)**  **ETK-M--EFVLILELLS----GGELF-EFLS---------------------EQDFLTEDEAVGFLIQVIRAIEYLH-DLSIVHLDIKPENIVL-KNRT-**

**Nv_TRIO(XP_032241348.2)**  **DTP-K--NYIIVTELLA----GGRLF-DYLV---------------------VMDALTEKVAIGYMHQVVEGVQHLH-DLNIVHLDLKPQNLLLDGGPL-**

**Nv_STK17(XP_001629087.2)**  **ETR-T--ELILVLELAL----GGDLH-RHCV---------------ALDSDEPASSRSEKEVVYLLRQILEGIRHLH-KQNYVHLDIKPNNILL-MTDEI**

**Om_TRIO_like_A(KAI6652169.1)**  **QLS-D--TGRLILELVS----GEAML-DRIV---------------------SEDLLTEAVVVRYLHQILLAVLYLH-DSSIAHNDLRPSNILT-TSTE-**

**Om_TRIO_like_B(KAI6652169.1)**  **FTP-S--SYILVLEHMP----AGNLL-QYLS--------------------LEKTICTESQLISIISQLIELIVYLS-SVGVVHCNLEPECLLVGERGD-**

**Om_STK17(KAI6649438.1)**  **EGN-L--VMIIILELCT----GGDLF-HSIS----------------------AEPFTERESVFVLKQVIDALTFLH-KKSIIHLDIKPENILLINETK-**

**Os_CON(XP_036371388.1)**  **EQP-G--EIVMILEFLS----GGELF-DRLI--------------------SNDYTFTEEDCISYMRQICQGVQHMH-HKNIIHLDLKPENVMCVTKDS-**

**Os_DAPK(XP_029643431.1)**  **ETK-T--EVILILELVP----GGELF-DYLS---------------------ERDKLCEAEASAFIKQILDGLRHLH-DRQIAHLDLKPENILM-VNQT-**

**Os_TRIO(XP_029657616.1)**  **ETK-T--NYILLMEIIP----SGRLF-EFIC---------------------IRGHFDELQAAEYVQQILDAIQYLH-NCRIAHLDIKPENIVVDCGLN-**

**Os_MYLK(XP_029655504.1)**  **ESP-T--GFVIVTEFVC----GGELF-KRIV--------------------DDDFVLTEQDCAHFVHQICEGVRYLH-DKQIIHLDLKPENILC-IAKS-**

**Os_OBSCN_A(XP_036361934.1)**  **ETV-K--SYVLITELIH----GTELI-EHIL---------------------TTYNWTETDAAFYIQQLLLALEELR-IRGVAHLDIKPGNIIY-EEKT-**

**Os_OBSCN_B(XP_036361934.1)**  **LFN-N--IHTVIFEYVD----SENII-DHLS---------------------SRCSYTEDDAARIITQLLDALQYLH-FLCIIHLNIQPENILL-GRMC-**

**Os_STK17(XP_029636789.1)**  **ESP-T--EMILITEYCS----GGELF-HECV---------------------IEESFKESDVVRLLGEILEGVLFLH-RKNILHLDLKPQNILL-TKPFP**

**Os_twitchin(XP_036360273.1)**  **DDK-Y--EMVLIFEFLS----GGELF-DRIA--------------------AEDYKMSEAEVINYMRQVCEGLKHMH-ENSIVHLDIKPENIMCETKKS-**

**Pc_DAPK(XP_045596115.1)**  **DNG-Q--HVILVLELVR----GGELF-EHIS---------------------ERERLSEEEASAFLHQILQGVRHMH-SLGIAHLDLKPENVLL-LSKN-**

**Pc_MYLK(XP_045598454.1)**  **DMT-K--EMCLVLEIVD----GGELF-ERVI--------------------DDDFVLSERACTVFIRQICEGVEFIH-SKNILHLDMKPENILC-LSRE-**

**Pc_OBSCN(XP_045586627.1)**  **EGP-K--NMVLVTHLCG----GGDLL-GALT---------------------QRQHLTEYEVCVIIRQVLLGLDYMH-DHYIAHLGLNIGDILLVRPNG-**

**Pc_projectin(XP_045599281.1+BA** **EDD-D--EMVLIFEFLS----GGELF-ERIT--------------------AEGYVMSEAEVINYMRQICEGVKHMH-EKNIIHLDVKPENIMCQTKTS-**

**Pc_STK17(XP_045598575.1)**  **ETT-S--EIILVLELAE----GGELQ-RVID---------------------EEENLEEGVVCRYMINILGALRFLH-AHNIAHLDLKPQNLLL-MGQHP**

**Pc_stretchinMLCK(XP_045616175.** **ERP-R--EMVMVMEYIS----GGELF-ERVV--------------------ADDFALTERDCILFVRQICEGVQYMH-KSLIVHLDLKPENILC-VRRT-**

**Sk_CON(XP_006823011.1)**  **ETD-E--EIIMVLEFLS----GGELF-ERLI--------------------DENYVLTEPEVVIYMRQLCDGIKYMH-EKNILHLDIKPENILCATRTG-**

**Sk_DAPK1(XP_006823401.1)**  **ETP-T--EVVLILELVS----GGELF-EFLA---------------------EKDHVCEEEAAKFTRQMLEGVKHLH-EKNIVHLDLKPENVML-LNRN-**

**Sk_TRIO(XP_006819276.1)**  **DTA-M--NLILILELIP----NGRLF-DYIV---------------------SKPLFTESIAVGYLHQMISAIEYLH-NCRISHLDIKPENFAVEANNV-**

**Sk_MYLK(XP_006823003.1)**  **ATG-D--SLVLILEFVS----GGELF-ERVV--------------------AEDFQLTEKEAVFFLRQITEGVEFMH-EKHILHLDMKPENILC-VRPK-**

**Sk_OBSCN_A(XP_006823009.1)**  **ETK-T--ELIMIMEMVT----GGELF-EKLI---------------------QEDCLTESEAVYFLRQVLEGLEHMH-KRNVVHLDLKPENILLVKPCD-**

**Sk_OBSCN_B(XP_006823009.1)**  **LTP-R--FLILIMERYY----GGPVT-RYLA---------------------SKSFYSEDEVVEFLRQLLDAVGFIH-SQSIVHLDIRPDNVLLESRRR-**

**Sk_STK17(XP_002741442.1)**  **ETR-H--ELILVLELAA----GGELH-RHCV------------------CEKEETSFTERDVVRLLKQILEAVQYLH-ERNVVHLDIKPSNILLTHSQPA**

**Sk_twitchin(XP_006825350.1)**  **DSG-D--QLVMVLEFLS----GGDVF-DRVL--------------------DSNYVLTEQEVALYAKQIVEGLNFMH-SKSIMYLDLKPENVLYESKKG-**

**Sp_CON(XP_030830392.1)**  **ETN-E--EIIMILEFLS----GGELF-DRLV--------------------DENHILTEPEVIFYMKQTCEGVKHMH-ERHLVHLDLKPENIMLCARNS-**

**Sp_DAPK(XP_030832558.1)**  **ELQ-K--EVVLILELVT----GGELF-HYLA---------------------EEDHVNEEVAAQFVKKILEALKHMH-DRNICHLDLKPENIML-LNRN-**

**Sp_TRIO(XP_011680751.1)**  **ETH-G--QYILVLDLIP----DGRIL-EYLV---------------------SLSQVTERQVIGFIRQLVQAVNYLH-SNGIIHLDIKPENVMMEKALG-**

**Sp_MYLK_A1(XP_030830851.1)**  **QSS-R--QTVMILECIT----GGELF-ERIV--------------------DDTFDLTESEVISYMRQICAGVQHMH-HHNIMHLDLKPENIMC-VNRT-**

**Sp_MYLK_A2(XP_030830851.1)**  **ESP-K--QMIMVLEIVN----GGELF-ERVI--------------------DDDFGLTESDVIEFMRQICAGVHHMH-STNILHLDLKPENILC-IDKT-**

**Sp_OBSCN_A(XP_030830075.1)**  **ETK-R--EVVMAMEILT----GGELF-ERIV---------------------QRDSFSESEAVGFLKQLIDGLIYMH-DRNVVHLDLKPENILLVAPES-**

**Sp_OBSCN_B(XP_030830075.1)**  **LTN-R--HVMLVTERYY----GGTVL-KYLT---------------------KEDTYTESTVVRIVAQVLDALEHLH-MMNVVYLDLRHDNLLMESRRK-**

**Sp_STK17(XP_030846519.1)**  **ETS-T--EVILVLEYAS----GGELH-QYCV-------------------ADKEDGFCEKDVVRLLQQILEGVHYLH-SQNIAHLDLKPQNILLTSKETS**

**Sp_twitchin(XP_030829654.1)**  **DMD-D--KFVLITDFIS----GGDAF-QRAK---------------------ERGTLSEEVVARYTRQVCEGLLHIH-LQNHMHLALRPQSILFQTKRS-**

**Ta_CON_A1(GHJI01005353.1)**  **QTP-T--DYVMVLEFIS----GGELF-DRIV---------------------EKEYLSEKEAAEYITQVLEGVQHMH-QNNIIHLDLKPENILCLSNDS-**

**Ta_CON_A2(GHJI01005353.1)**  **QTD-E--YVVMIMEVLR----GGELL-DRLI---------------------KRETLLEVEVIYYMQQVLQGLKFMH-DSNILHLDLKPENLML-FEKD-**

**Ta_TRIO(GHJI01000868.1)**  **DCE-D--IYMIVMEYLP----GGRLF-DYLC---------------------DNDCINELQIQNYMKQIGDGLRFLH-QMSVVKVNLKPENIILEGCEH-**

210 220 230 240 250 260 270 280 290 300

....|....|....|....|....|....|....|....|....|....|....|....|....|....|....|....|....|....|....|....|

**Aa_CON_A(GHAI01170451.1)**  **-SN-Q-VKLIDFGLAKKY-NP-KETIQVLCGTPEFIAPEV-ISYDTLTTACDMW-SIGVITYVLLSGLSPF------MGDN-------------D--AET**

**Aa_CON_B(GHAI01170451.1)**  **------LKLIDYGSSRKI-NSVQGEVGEMVGSAEFMSPEM-INFEPVSSFSDVW-GVGVVTYALLSGFSPF------ACED-------------E--DET**

**Aa_TRIO(GHAI01159172.1)**  **-PR---LKLVDFGDAVQI-TQ-NPYMHELNGSPEFCAPEV-INGDAVSLATDLW-SVGVIIYVLISGVSPF------YSDN-------------Q--ERA**

**Am_CON_A1(XP_044172413.1)**  **-GK-EDLKLIDFGMARVL-EQ-GKSYKVACGTPEFVAPEV-ISYEPISLSSDMW-SVGVIAYVLLSGLSPF------MGDD-------------D--NET**

**Am_CON_A2(XP_044172413.1)**  **-SK-Q-IKLIDFGLARKY-NP-KENLKVMFGTPEFVAPEV-IKYERIGPATDVW-SIGVIAYILLSGLSPF------MGDS-------------D--AET**

**Am_CON_B(XP_029213098.1)**  **-LG---LKLIDYGSARRIKNWEAGDTLAIVDYVAFTAPEL-LNFTPVSGWTDMW-SIGVLLYCLISGELPFCVECTEDEDE-------------D--EKL**

**Am_DAPK(XP_029207283.2)**  **-EK-K-IKLIDFGLAKII-PP-GEIVRAIMGTPEFVAPEV-LNFEPVGTQTDMW-AVGVLTYILLSGASPF------LADD-------------D--NET**

**Am_TRIO(XP_029203193.2)**  **-PK---VKIVDFGCATEITDS-EPVIRQVFGNPEFSAPEL-VNRKPVHFTTDLW-SVGVITYVILSGVSPF------QEDT-------------V--EQT**

**Aq_TRIO(XP_019864448.1)**  **DFT---VKLIHLSEAKFV-HS-DVKQLSCKVVTGYEAPEV-ISSESVGTKTDMW-SVGVMLYHIFCGLLPC-----------------------------**

**Aq_TRIO_like_A(XP_019854769.1)** **-AP-I-LKLTDFGSAQEV--P-YAGLSVRPDLIEFSSPEV-VFDDNVTKCTDLW-STGVITYLLLSGVSPF------FHEY-------------P--SVS**

**Aq_TRIO_like_B(XP_019854769.1)** **--E---VKIIDFSHSKYL-HS-NPQLEILPQQINYLSPEA-LQCSPLALSTDLW-SVGAVALFMLSGQCPF------VSDT-------------R--DQL**

**Aq_STK17(XP_003383221.1)**  **-LD---IRIADFGLALQI-AP-GEQVKTLVGTAEYVAPEI-LNYEPLSVAADMW-SIGALTYALLTGYSPF------QGET-------------H--SDT**

**Bf_CON(XP_035661587.1)**  **-YY---IKITDFGRSCQA-KP-GQKVNMSYISAEFMAPEV-LNSESVGTSTDMW-SLGCIVYLLLGGTSPF------EGES-------------E--ADT**

**Bf_DAPK(XP_035665897.1)**  **-QA-I-IKLIDFGISRRI-ED-GKNEIQMLGTPEFVAPEV-IAYEPLGLYTDMW-AVGVITYILLSGCSPF------LGDN-------------K--QET**

**Bf_TRIO(XP_035662010.1)**  **-TP-K-IKLIDFGDAKQISNS-RFYIHNLLGSPEFAAPEL-VNGHPVCLNTDMW-SVGVLTYVLLSGVSPF------QDES-------------V--EET**

**Bf_MYLK(XP_035660911.1)**  **-GN-E-IKLIDFGLARRY-NP-QEELKVMFGTPEFVAPEV-INYDKIGYGTDMW-SVGVICYVLLSGLSPF------MGED-------------E--AET**

**Bf_MYLK2(XP_035661607.1)**  **-GT-A-IKLIDFGLARKF-DP-KEDNRVMFGTPEFVAPEV-INFDSIDYSTDMW-SIGVITYVLLSGLSPF------MGDT-------------D--QET**

**Bf_OBSCN_A(XP_035660917.1)**  **-SG-Q-LKLCDFGLARQL-LP-GTPEICRFGTPEFVAPET-VAKEPVHLTTDIW-STGVLLYVLLSGVSPF------MGNN-------------D--KET**

**Bf_OBSCN_B(GESZ01107032.1+XP_0** **-LD---VRLTDFGCARTF--P-SKTLGELQSTPEFVAPEV-VNYETAGPAADIW-SVGVIAFLCLSAHSPF------LGVG-------------D--HQT**

**Bfo_TRIO_like(GHXY01112106.1)**  **------VKIINYELAHYVDEE-NVYLEPSMLDSEFAAPEL-LLGRVSTSAADIW-SVGALAYLLLFSDSPF------YVQG-------------------**

**Bf_STK17(XM_035832943.1)**  **CGD---IKLVDFGLARRV-NV-HEEIREIVGTPDYVAPEV-LSFEPLSTATDMW-SIGVLAYVMLTGHSPF------LGDT-------------K--QET**

**Bm_TRIO_like(GKLW01050522.1)**  **------VKIINYELAHYVDEE-NVFLEPSMLDPEFAAPEL-LQGRVSTTAADIW-SVGVLTYLLLYSDSPF------FLQG----------------QII**

**Ce_DAPK(NP_490840.2)**  **-DS-Q-IKIIDFGLSREI-EP-GAVVKDMVGTPEFVAPEV-VNYEALSPATDMW-AVGVVTYILLSGGSPF------LGDN-------------R--DET**

**Ce_MYLK(NP_509689.1)**  **-GN-R-IKLIDFGLARHY-DG-TQELKYMAGTPEFAAPEV-IKFEKLDYHTDMW-SIGVITYILLSGYSPF------LGDN-------------L--GET**

**Ce_OBSCN_A(NP_001343714.1)**  **-QD-DKLKLADFGQARRL-LR-GLITGEIKGSPEFVSPEI-VRSYPLTLATDMW-STGVLTYVLLTGLSPF------HGDN-------------D--NET**

**Ce_OBSCN_B(NP_001343714.1)**  **-WV---VKLVDFGRAQKV----SGAVKPVDFDTKWASPEFHIPETPVTVQSDMW-GMGVVTFCLLAGFHPF------TSEY-------------DREEEI**

**Ce_titin(G4SLH0.2)**  **-NE---LKIIDFGLARKL-DP-KKSVKLLFGTPEFCAPEV-VNYQPVGLSTDMW-TVGVISYVLLSGLSPF------LGDS-------------D--EDT**

**Ce_twitchin(NP_502274.2)**  **-NE---LKLIDFGLTAHL-DP-KQSVKVTTGTAEFAAPEV-AEGKPVGYYTDMW-SVGVLSYILLSGLSPF------GGEN-------------D--DET**

**Cg_CON_A1(XP_034304307.1)**  **-KD---VKIIDFGLTQRL-EE-GKNVKVLFGTAEFCAPEI-INFEPVSFTTDMW-SLGVVTYVLLSGYSPF------AGET-------------D--HET**

**Cg_CON_A2(XP_034304307.1)**  **-NN-L-IKIIDFGLARRH-LE-GDSLRVMFGTPEFIAPEV-VNYEEIGFPTDIW-SVGVICYVLLSGLSPF------MGDS-------------D--VET**

**Cg_TRIO(XP_034303731.1)**  **-SA-C-LKLIDFGDARIV-YN-DNYIHEYAGSAEFRAPEV-IRGQAVSTLTDIW-SVGVILYVLLSGVSPF------LDES-------------Q--EET**

**Cg_MYLK(XP_011441364.2)**  **-GN-I-IKIIDFGLARKF-DP-SGDLRILFGTPEFMAPEV-VNFDPASPATDMW-SIGVICYVLLSGLSPF------VGDT-------------D--AET**

**Cg_OBSCN_A(XP_034299497.1)**  **------LTLIDFGFSERI-QR-NKEVRKNYGTPGFCSPEQ-VHNEPVSEASDVW-SIGATVYTLLTGLSPF------GGST-------------E--QEV**

**Cg_OBSCN_B(XP_034299497.1)**  **-LD---VRIVDFGLVQKV-IK-EGQIVPRDGNPEFMAPEV-VVKETTSYPADIW-SVGVLAFLLLSGESPF------KGQD-------------E--ETT**

**Cg_STK17(XP_011422577.1)**  **HGD---IKVCDLGFACLV-NT-GEDIRDIIGTPDYVAPEV-LSYEPLGLYTDMW-SLGVLTYVMLTAHSPF------AGKD-------------N--QET**

**Cg_twitchin(XP_034332726.1)**  **-TN---VKMIDFGLATKL-NP-DEIVKVTTATAEFAAPEI-VDSEPIGFYTDMW-AVGVLAYVLLSGLSPF------AGED-------------D--LET**

**Ct_CON(ELT96026.1+AMQN01011565** **-LD---IKLVDFGMSQML-NP-DKSVRVLFGSAEFSAPEI-VSYEPVSFASDVW-SLGVCAFILMTGYSPF------LGKT-------------I--QDT**

**Ct_DAPK(ELT88671.1)**  **-ST-R-LKIIDFGLSRKL-DE-GVEVKDITGTPEFVAPEI-VNYDPLCTATDMW-SIGVITYILLSGCSPF------LGDD-------------K--QET**

**Ct_TRIO(ELU10047.1)**  **--N---VVLVDFGDARLI-EN-DFNVLPLVGSPEFSAPEI-VNSSPVGLATDIW-AIGVLSYVLLSGISPF------LDES-------------P--DET**

**Ct_MYLK(AMQN01000602.1+ELU1638** **-GN-R-IKIIDFGLAREW-NP-KRDLRVLFGTPEFMAPEV-VQYEPITFATDMW-SVGVISYVLLSGLSPF------MGDT-------------D--ADT**

**Ct_MYLK2(ELT87547.1)**  **-SN-E-IKIIDFGLARKY-DP-TKSAKVMFGTAEFVAPEV-VNYDPISYTTDMW-SVGVICYMLLSGLSPF------MGEN-------------D--AET**

**Ct_OBSCN(ELU05514.1)**  **-DD---LKIIDFGYARRY-NP-ARRLYSKYGTPEFVSPEI-ASEDQVTLASDLW-SVGVIAYILVSGISPF------HRDT-------------A--RET**

**Ct_STK17(ELU16209.1)**  **TFD---VKLCDLGLARQV-NC-GQETRDLIGTPDYVAPEI-LNYEPIHTSCDIWYVVGVLTYVLLTGFSPF------AGDN-------------K--QET**

**Ct_twitchin(ELT89874.1)**  **-NT---VKMIDFGLASKL-DP-ESVVKVSTATAEFASPEV-VDNEPVGFYTDMW-AVGVLSYVLLSGLSPF------AGEN-------------D--QET**

**Dj_DAPK1(IAAB01043597.1)**  **-TQ-K-IKIIDFGFSKEVNDS---PILELQGTPEFVAPEI-INYDPLTFATDMW-SIGIIAYVMLTGTSPF------YHET-------------Q--VET**

**Dj_DAPK2(IAAB01051648.1)**  **-ER-K-IKLIDFGLAREI-KK-QDTIIEMQGTAEFLAPEA-INYDAISYATDMW-AIGVITYIMLTGVSPF------SGDD-------------Q--SST**

**Dj_MYLK(IAAB01042781.1)**  **-GF-Q-IKIIDFGLARQY-DS-HKDIRVMFGTPEFIAPEI-ITYEPISPGTDMW-AVGVICYVLLSGLSPF------MGEN-------------D--GET**

**Dj_STK17(IAAB01043480.1)**  **STD---IALCDFGLATQL-SS-GRVHRELVGTPDYIAPEI-INYEEISMFTDMW-SLGVLTYYLLTSESPF------MADT-------------K--EMT**

**Dj_twitchin(IAAB01071515.1)**  **-NR---IKIIDFGLSTKL-NP-NEPVKVTTATAEFAAPEV-AEIEPVGFYTDMW-AVGVLSYVLLSGLSPF------GGDD-------------D--YET**

**Dm_MYLK(NP_001260832.1)**  **-GN-R-IKIIDFGLARKF-DP-DKRLRVLFGTPEFVAPEV-VNFDCISYGTDMW-SVGVICYVLISGLSPF------MGEN-------------D--IET**

**Dm_OBSCN_A(XP_034109413.1)**  **-DI---IKVSDFGLSRKI-NR-HNLSTLDYGMPEFVSPEV-VNKEGVNFSHDMW-TVGLITYVLLGGHNPF------LGID-------------D--RET**

**Dm_OBSCN_B(XP_034109413.1)**  **-IQ---VKLVDFGSAKKV-NK-LGMKVTPCGSLDFQPPEM-INDEPIFPQSDIW-SLGALTYLLLSGCSPF------RGAD-------------E--YET**

**Dm_projectin(NP_995598.1)**  **-TN---VKLIDFGLATRL-DP-NEVVKITTGTAEFAAPEI-VNREPVGFYTDMW-ATGVLSYVLLSGLSPF------AGDN-------------D--VQT**

**Dm_STK17(NP_001162723.1)**  **EDG---LKLCDFGISRVV-CE-GINVREMAGTPDYVAPEV-LQYEPLSLLTDIW-SVGVLTYVLLSGFSPF------GGDT-------------K--QET**

**Dm_stretchinMLCK(NP_725510)**  **-SH-Q-IKIIDFGLAQRL-DT-KAPVRVLFGTPEFIPPEI-ISYEPIGFQSDMW-SVGVICYVLLSGLSPF------MGDT-------------D--VET**

**Ef_CON(GIUK01093179.1)**  **GND---LKVVNFGIAQRV-DP-NRTVRVLFSTIEYCCPEI-LMFAPVSFAADLW-SLGVLAYVLLSGVSPF------LGRT-------------T--DET**

**Ef_DAPK(GIUK01016195.1)**  **-SS-Q-LKIIDFGLSRQL-CS-NIECHEIVGTPEFVAPEV-INYDALCLATDMW-SIGVIAYILLSGYSPF------LGNN-------------K--QET**

**Ef_DAPK(GIUK01061946.1)**  **-SR-K-LKIIDFGLSRKL-EA-NSECRDITGTPEFVAPEV-INYDPLSSATDMW-SIGVITYIMLSGCSPF------LGDD-------------K--QET**

**Ef_MYLK2(GIUK01050934.1)**  **-SN-E-IKIIDFGLARKF-DP-AKEFKIMFGTAEFVAPEV-INYDAISYKTDMW-SVGVICYVLLSGLSPF------LGNN-------------D--KET**

**Ef_MYLK(GIUK01094627.1)**  **-GN-R-IKIIDFGLAREF-NP-KVDTRVMFGTPEFMAPEV-VQYDPIDFSTDIW-SIGVICYVLLSGLSPF------MGDT-------------D--VET**

**Ef_MYLK(GIUK01083042.1)**  **-GN-R-VKIIDFGLARVF-DP-KKDTRVLFGTPEFMAPEV-ILFDPIHYATDMW-SIGVMCYVLLSGLSPF------MGSN-------------D--TAT**

**Ef_OBSCN_A(GIUK01083331.1)**  **-DE---IKVIDFGFARRF-NP-TRRMMVKYGTPEFCAPEV-AREESVTPAADLW-SVGVIAYILLSGISPF------HKGS-------------P--RDT**

**Ef_OBSCN_B(GIUK01083331.1)**  **-FD---VKLIDFGQAHTITSA-EGQKVDKTGSTEFMAPEK-VSREDVGVAADVW-GVGVLTFVLLSGISPF------YSEA-------------D--EDT**

**Ef_STK17(GIUK01063378.1)**  **HGD---IKLCDFGLARLF-NC-GHDILEIIGTPDYVAPEV-LDYKPLGSACDMW-SIGVLTYVMLTGCSPF------AGDT-------------T--QET**

**Ef_twitchin(GIUK01013637.1)**  **-RD---VKLIDFGLSTKL-DP-EQAVKVSTATADFAAPEI-ADHEQVGFYTDMW-AVGVLSYVLLSGLSPF------AGAN-------------D--QET**

**Ef_twitchin(GIUK01097374.1)**  **-RD---VKLIDFGLSARL-DP-EQIVKVSKATAEFASPEI-AEHEPVGFYTDMW-AVGVLSYILLSGLSPF------AGAD-------------E--QET**

**Eg_DAPK(KAH9282855.1)**  **-TK-R-IKIIDFGLARRL-NP-NEVIQDMAGTPEFCAPEI-VNFDPITFATDMW-AIGVMTYILLSGISPF------AGDT-------------Q--VET**

**Eg_MYLK(XP_024351289.1)**  **-GF-K-IKIIDFGLAREV--T-NGDLRVMFGTPEFVAPEV-IAFDPVTYATDMW-SLGVVCYVLLSGLSPF------MGDN-------------E--SET**

**Eg_STK17(KAH9283880.1)**  **STD---VFITDFGLASVL-NK-SKPHKELAGTPDYAAPEV-ISYDPVSFATDMW-SVGVLAYFLLTGVSPF------LAES-------------K--ALT**

**Eg_twitchin(KAH9283145.1)**  **-TD---IKLVDFGLATKL-NP-QDEVRVSTATPEFAAPEI-ADHNPVGFYTDMW-AVGVLSYILLSGISPF------TGSD-------------T--MDT**

**Hc_TRIO_like(GHXS01109180.1)**  **------VKLINYELAHYVEDE-NVYLETSMLDDEFGAPEL-LLGRVSSTAADIW-AVGVLTYLLLYSDSPF------YSQG----------------HII**

**Hs_CON(NP_003310.4)**  **-ST---IKIIEFGQARQL-KP-GDNFRLLFTAPEYYAPEV-HQHDVVSTATDMW-SLGTLVYVLLSGINPF------LAET-------------N--QQI**

**Hs_DAPK1(NP_004929.2)**  **PKP-R-IKIIDFGLAHKI-DF-GNEFKNIFGTPEFVAPEI-VNYEPLGLEADMW-SIGVITYILLSGASPF------LGDT-------------K--QET**

**Hs_DAPK2(NP_055141.2)**  **PIP-H-IKLIDFGLAHEI-ED-GVEFKNIFGTPEFVAPEI-VNYEPLGLEADMW-SIGVITYILLSGASPF------LGDT-------------K--QET**

**Hs_DAPK3(NP_001339.1)**  **PNP-R-IKLIDFGIAHKI-EA-GNEFKNIFGTPEFVAPEI-VNYEPLGLEADMW-SIGVITYILLSGASPF------LGET-------------K--QET**

**Hs_KALRN(NP_001375348.1)**  **-VP-R-VKLIDLEDAVQI-SG-HFHIHHLLGNPEFAAPEV-IQGIPVSLGTDIW-SIGVLTYVMLSGVSPF------LDES-------------K--EET**

**Hs_MYLK(NP_444253.3)**  **-GT-R-IKLIDFGLARRL-EN-AGSLKVLFGTPEFVAPEV-INYEPIGYATDMW-SIGVICYILVSGLSPF------MGDN-------------D--NET**

**Hs_MYLK2(NP_149109.1)**  **-GH-L-VKIIDFGLARRY-NP-NEKLKVNFGTPEFLSPEV-VNYDQISDKTDMW-SMGVITYMLLSGLSPF------LGDD-------------D--TET**

**Hs_MYLK3(NP_872299.2)**  **-GH-Q-IKIIDFGLARRY-KP-REKLKVNFGTPEFLAPEV-VNYEFVSFPTDMW-SVGVITYMLLSGLSPF------LGET-------------D--AET**

**Hs_MYLK4(NP_001012418.2)**  **-AK-Q-IKIIDFGLARRY-KP-REKLKVNFGTPEFLAPEV-VNYDFVSFPTDMW-SVGVIAYMLLSGLSPF------LGDN-------------D--AET**

**Hs_OBSCN_A(NP_001373054.1)**  **-RE-D-IKICDFGFAQNI-TP-AELQFSQYGSPEFVSPEI-IQQNPVSEASDIW-AMGVISYLSLTCSSPF------AGES-------------D--RAT**

**Hs_OBSCN_B(NP_001373054.1)**  **------LKVVDLGNAQSLSQEKVLPSDKFKDYLETMAPEL-LEGQGAVPQTDIW-AIGVTAFIMLSAEYPV------SSEG-------------A--RDL**

**Hs_SPEG_A(NP_005867.3)**  **-GEQQ-VRICDFGNAQEL-TP-GEPQYCQYGTPEFVAPEI-VNQSPVSGVTDIW-PVGVVAFLCLTGISPF------VGEN-------------D--RTT**

**Hs_SPEG_B(NP_005867.3)**  **------LKIVDFGSAQPYNPQALRPLGHRTGTLEFMAPEM-VKGEPIGSATDIW-GAGVLTYIMLSGRSPF------YEPD-------------P--QET**

**Hs_ST17A(NP_004751.2)**  **LGD---IKIVDFGLSRIL-KN-SEELREIMGTPEYVAPEI-LSYDPISMATDMW-SIGVLTYVMLTGISPF------LGND-------------K--QET**

**Hs_ST17B(NP_004217.1)**  **LGD---IKIVDFGMSRKI-GH-ACELREIMGTPEYLAPEI-LNYDPITTATDMW-NIGIIAYMLLTHTSPF------VGED-------------N--QET**

**Hst_TRIO(GKDX01093008.1)**  **-MDYPDVKLIHFDRAREIEPNSKLVVKKLDNHTEYEAPEI-LSGKPVTLATDMW-SLGVVLYTLLSGSSPF------LVDS-------------L--DLT**

**Hst_TRIO_like_A(GKDX01095742.1** **-NP-V-VKLIDFGTARDLSSP-ELDPTVCASSPEFCAPEV-VNQEPLCLASDMW-SLGVLTYILLSGLSPF------FTDT-------------P-GQLV**

**Hst_TRIO_like_B(GKDX01095742.1** **--D---LKITDFGMARYI-EA-GNQLDEVPDHPDYMAPEA-VQLQPLTTAVDIW-SVGAIAHFMLTGRSAF------SGPT-------------S--SIT**

**Hs_TRIO(NP_009049.2)**  **-KP-T-IKLADFGDAVQL-NT-TYYIHQLLGNPEFAAPEI-ILGNPVSLTSDTW-SVGVLTYVLLSGVSPF------LDDS-------------V--EET**

**Hv_CON_A(XP_047137784.1)**  **-ST-Q-IKLIDFGLARRY-EK-GGTLRVLFGTPEFMAPEV-ISYDEVTKVTDTW-SIGVITYVLLSGLSPF------AGDD-------------D--SET**

**Hv_CON_B(XP_047137784.1)**  **------LKLIDYGVSRKIVSK-EGEVGEMVGTAEFMAPET-INFEPVNNRTDIW-SVGVVTYALLSGVSPF------ATDD-------------E--DET**

**Hv_DAPK1(XP_012558089.1)**  **-RN-E-IKLVDFGLAQRL-TP-GKDLKEMMGTPEFVAPEI-VSYETIGCYTDMW-AIGVLAFILLSGCSPF------LGEN-------------N--QET**

**Hv_DAPK2(XP_047139830.1)**  **-SS-E-IKLVDFGLAQRL-VP-GKDVREIMGTAEFVAPEI-VNYEPIGCYTDMW-AIGVLAYILVSGTSPF------LGDT-------------N--EET**

**Hv_TRIO(XP_047137671.1)**  **-NN---IVLSDFGDAIRL-RS-VPYQHEMNGNPEFLAPEI-ITGKAVSLLTDMW-SIGVVVYVLLSGVSPF------FSDN-------------S--TRL**

**Ml_TRIO_like(GFAT01019191.1)**  **------VKIINYELAHYVDEE-NVFLEPSMLDGEFAAPEL-LQGRVSTTAADIW-SVGVLTYLLLYSDSPF------YLQG----------------QII**

**Nv_CON_A1(XP_048579265.1)**  **-GK-EDLKLIDFGMAHII-EK-GKDLKLACGTPEFVAPEV-LAYEPIKLAADMW-SIGVIAYVLLSGLSPF------MGDD-------------D--NET**

**Nv_CON_A2(XP_048579265.1)**  **-SR-Q-IKLIDFGLARKY-NP-KENLKVMFGTPEFVAPEV-LTYDRITPATDMW-SIGVIAYVLLSGLSPF------MGDN-------------D--AET**

**Nv_CON_B(XP_048579265.1)**  **------VKLIDFGNARKLKSR-HGEVGVVVGNPSFTAPEV-LSFEPVNMAADMW-SVGIVTYALLSGQLPF------PGDN-------------D--DAV**

**Nv_DAPK(XP_032239611.2)**  **-RPLH-LKLIDFGLARKI-SK-GEPVREMMGTPEFVAPEI-IDFEVVGFPTDMW-SIGVLTYIMLSGASPF------LGDD-------------N--NET**

**Nv_TRIO(XP_032241348.2)**  **-PK---VKIIDFGSAHIL-SG-SPVNHKVYGSPEFAAPEL-IMEEPLTFKTDTW-SIGVITYVMLSGVSPF------QADT-------------T--DEM**

**Nv_STK17(XP_001629087.2)**  **YPE---IKIIDFGLARRI-KP-GEQICLIVGTPEYVAPEI-LEFEPVGKPSDIW-SIGVLAYVMLTGMSPF------AGDD-------------K--HET**

**Om_TRIO_like_A(KAI6652169.1)**  **-PP-Q-IKLADFGSAFKI--D-NFSQTIIPLTTEFTSPEA-LEDLPLSGQVDLW-ALGVICYIMLTGVSPF------FAES-------------HEFSHT**

**Om_TRIO_like_B(KAI6652169.1)**  **------IKVTDFSLSRRSGKH-VPASSLSLSARGFVAPEV-LRGACVSSKSDIW-TLGCLCILLTTGTI--------LSNS----------------QTG**

**Om_STK17(KAI6649438.1)**  **-LD---IKLADFGLAVKL-SP-IEEFKRIIGTPEYVAPEV-INFEPLSTSSDMW-SLGALTYALLCGYSPF------LPEDDDSTDEEVSRLVEQ--ADI**

**Os_CON(XP_036371388.1)**  **-TG---IKLIDFGLAQKI-ED-GKSIKVLFGTAEFCSPEI-INFEPVSFSSDMW-SLGVVAYVLLSGYSPF------AGDD-------------D--QET**

**Os_DAPK(XP_029643431.1)**  **-SQ-R-IKLIDFGLSRKL-KP-GIDSRAMMGTAEFVAPEV-VSYEPLTLATDMW-SVGVITYILLSGTSPF------LGDS-------------P--QET**

**Os_TRIO(XP_029657616.1)**  **-SG---VKLIDFGDARHI-YN-NYYIHPVVGNPEFMAPEL-VSGTPVGLLTDIW-SMGVILYVLLSGVSPF------LDES-------------Q--EET**

**Os_MYLK(XP_029655504.1)**  **-NN-F-IKIIDFGLARKL-QV-GDSIKVLFGTPDFIAPEV-VNYDEISFATDLW-SLGVICYVLISGLAPF------TGET-------------N--VET**

**Os_OBSCN_A(XP_036361934.1)**  **-DK---IKLIDFGFAKKIHNA---PLKLNYGTPEFASPEV-VCSEVISESTDLW-SVGVLTYILLSGISPF------HCES-------------V--SET**

**Os_OBSCN_B(XP_036361934.1)**  **-PI---LKLKDFTLAQKIVTP-IGKHVPQCGSPEFMSPEV-VVGEPAGVAADVW-GVGVISTLLLSGETPF------VGST-------------V--EET**

**Os_STK17(XP_029636789.1)**  **EGN---IKICDLGLACLV-ND-GEDIREIVGTPDYVAPEV-LSYEPLGLSTDMW-SLGVLTYVMLSACSPF------DGES-------------K--QET**

**Os_twitchin(XP_036360273.1)**  **-TQ---VKIIDFGLATKL-NP-DEIVKVTTATAEFAAPEI-VDREAVGFYTDMW-AVGVLAYVLLSGLSPF------AGED-------------D--LET**

**Pc_DAPK(XP_045596115.1)**  **-RQ-H-IKLIDFGLSRVI-TQ-AEEVRDMMGTAEFVAPEI-VNYEPLCLATDMW-AIGVITYILLSGASPF------LGDT-------------Q--QDT**

**Pc_MYLK(XP_045598454.1)**  **-GN-R-IKICDFGLARRY-DP-RKKLQVLFGTPEFVAPEV-VNFEPISFGTDMW-SVGVICYVLLSGLSPF------MGHN-------------Y--VET**

**Pc_OBSCN(XP_045586627.1)**  **-LE---LKIGDLSLARQI-KM-NDLQPLDYGMPEFVAPEV-ANNEGVAFSADMW-AVGIITYILLSGTSPF------RGEN-------------D--RET**

**Pc_projectin(XP_045599281.1+BA** **-TN---VKLIDFGLATKL-DP-NEVVKISTGTAEFAAPEI-VEREPVGFYTDMW-AVGVLAYVLLSGLSPF------AGEN-------------D--IDT**

**Pc_STK17(XP_045598575.1)**  **QSD---VKLCDFGISRII-LS-DIEVREVLGTPDYVAPEI-LQYEPISLATDMW-SVGVLTYVLLTGHSPF------GGDT-------------K--QET**

**Pc_stretchinMLCK(XP_045616175.** **-SH-Q-IKLIDFGLARRF-NP-DDPCRVLFGTPEFIAPEI-INYELIGFASDMW-SVGVICYVLLSGLSPF------MGDN-------------D--AET**

**Sk_CON(XP_006823011.1)**  **-YD---IKLIDFGLARHM-DP-GEQIKVMFGTPEFVAPEV-VNFEPIGLPTDMW-TVGVMAYILLSGLSPF------LGDD-------------D--QET**

**Sk_DAPK1(XP_006823401.1)**  **-SQ-N-IKLIDFGLSRRI-VE-GTEIRDMIGTPEFVAPEV-VNYEALGLYTDMW-AVGVITYILLSGASPF------LGDN-------------Q--QET**

**Sk_TRIO(XP_006819276.1)**  **-TC-R-VKLIDFGDAINI-GS-KLFVHGLVGSPEFAAPEL-VNGHPVCLNTDMW-GVGVLTYVMLSGVSPF------LDES-------------I--EET**

**Sk_MYLK(XP_006823003.1)**  **-SN-K-IKIIDFGLARKY-NP-KESLKVMFGTPEFVAPEV-INYDQISEATDMW-SVGVICYVLLSGLSPF------MGDN-------------D--AET**

**Sk_OBSCN_A(XP_006823009.1)**  **-DN---IKLIDFGLARKI-LS-DKDVFVKFGTPEFVAPEV-VNKQPVTTATDLW-SLGIIAYVMLSGISPF------MGED-------------D--KDT**

**Sk_OBSCN_B(XP_006823009.1)**  **-ND---IRLIDFGSARRLPEK-GGVKVDADLIPEFMAPEA-VEGQLLNTSADIW-SIGILAFIMLSGVSPF------LFKD-------------K--SET**

**Sk_STK17(XP_002741442.1)**  **FGD---VKLVDFGLARLV-NA-NEEIREILGTLDYVAPEI-LSYEPITLATDMW-SIGVLTYVMLTGISPF------AADD-------------K--QET**

**Sk_twitchin(XP_006825350.1)**  **-SN---VKLIDFGMATKI-DP-EQKAKMVFGSPDFVAPEV-LNKDSVGFTTDMW-TVGVLCYMLLSGKHPF------GGDK-------------------**

**Sp_CON(XP_030830392.1)**  **-DD---IKIIDFGLTMEL-DP-DKPVKIMFGTPDFIAPEV-VNHEPIGLPTDMW-ALGVIAYLMLSGISPF------EGET-------------D--RET**

**Sp_DAPK(XP_030832558.1)**  **-TQ-N-IMLIDFGLSRRI-KP-GEDIRDIMGTAEFVAPEI-INFEPLSLNTDMW-AIGVITYILLSGLSPF------LGDD-------------Q--QET**

**Sp_TRIO(XP_011680751.1)**  **-RA-Q-IKLIDFGDAMDLWSSPPPFYHELNANPEFCSPEL-LGGNEIDYGTDVW-SIGALSYVMLSGISPF------LDES-------------L--EET**

**Sp_MYLK_A1(XP_030830851.1)**  **-GF-Q-LKIIDFGLARKY-EP-DNDVKVLCGTPEFVAPEV-ISYDAITPLTDMW-SVGVICYVLLSGLSPF------LGDS-------------D--SET**

**Sp_MYLK_A2(XP_030830851.1)**  **-GS-R-IKLIDFGLARDF-NP-AQSTKVMFGTPEFVAPEV-INYDVIGFTTDMW-SVGVICYILLSGLSPF------MGDN-------------D--AET**

**Sp_OBSCN_A(XP_030830075.1)**  **-DD---IKLIDFGLAAVL-KE-GEDITCKFGTPEFVAPEV-VNKQPVSTGADVW-GVGVIAFILLSGISPF------AGED-------------D--RQT**

**Sp_OBSCN_B(XP_030830075.1)**  **-DV---VRLIDFGSCRVIQKDGESKTKGIDVLPEFMAPELAVKGGSIDYETDIW-PLGVMVFTWLSGTSPF------LGRN-------------Q--EKT**

**Sp_STK17(XP_030846519.1)**  **ESD---IKLIDFGIARYL-NQ-GEEIRDIQGTPDYVAPEI-LNYDPITLSTDMW-SIGVLTYVMLTGISPF------AGDT-------------K--QET**

**Sp_twitchin(XP_030829654.1)**  **-DS---VKIIDFGLAAKL-DP-DERVKIAYAEPDYASPEV-LNGDQLGFSTDMW-SIGLIVYMLLSGLHPF------ENDA-------------D--R--**

**Ta_CON_A1(GHJI01005353.1)**  **-MD---IKLIDFGLAHKY-NP-KDKIKVICGTPEFVAPEV-INFEPISFSADMW-SVGVVTYILLSGLSPF------MGEN-------------D--GET**

**Ta_CON_A2(GHJI01005353.1)**  **-YD-D-IKLIDFGMARKF-QA-QDSLKVLFGTPEFVAPEV-VSYEKISPATDMW-SIGVITYVLLSGLSPF------MGDN-------------D--HDT**

**Ta_TRIO(GHJI01000868.1)**  **-KF---IKLVGFSHAICLDDC---HMIDFDGDVEFAAPEV-ISQSGISSMSDMW-SLGAITYLGLTGLSPF------YKKK-------------K--KDT**

310 320 330 340 350

....|....|....|....|....|....|....|....|....|....|....

**Aa_CON_A(GHAI01170451.1)**  **LANVTTAE-WDF-DDPVFEDISQEAKEFI-EDLLVKNQKK-RSTVKECFEHPWL**

**Aa_CON_B(GHAI01170451.1)**  **MASVTALD-YRF-EQKAFASITEEAKSFI-KRIIIRIPEK-RPGAGKLLEDPWL**

**Aa_TRIO(GHAI01159172.1)**  **CQNITEIR-YRF-PNEFFCDIGSEVKDLI-EELLIHDQSH-RPDAKECLRFPWI**

**Am_CON_A1(XP_044172413.1)**  **IQNVSNAE-WDF-EDEAFDVVSDMSKKFI-EELLIRDPGK-RSDVFHCLNHDWL**

**Am_CON_A2(XP_044172413.1)**  **LTNVQLAD-WDF-DDPVFDEISEEAKDFI-SSLLVLKTNK-RATVDQCLSHRWF**

**Am_CON_B(XP_029213098.1)**  **SQEIKKCK-WNL-DAKAFARCTSEVKKFI-TALLQFDVKK-RMSAEQAMKDDWL**

**Am_DAPK(XP_029207283.2)**  **FTNIQNVD-YEF-DEEYFSEISKQAKDFI-SKLLLKNPRD-RLSARDCLKHSWL**

**Am_TRIO(XP_029203193.2)**  **CQKITQVK-YHL-NPKHFEPISDQAKDFI-TSLLIKNPKK-RADCQHCLNSSWI**

**Aq_TRIO(XP_019864448.1)**  **LQSIKKKK--LF-EPRKFSYVSIELKDAI-LHLLQTKLCN-RLSSTELLQHKWL**

**Aq_TRIO_like_A(XP_019854769.1)** **SQRLSRAQ-YDL-SLQLFHSTSPQAIDFI-SALLKKQPAE-RLTAASALEHQWI**

**Aq_TRIO_like_B(XP_019854769.1)** **INNIHTLN-INYKINPIIQRLSLDCTNMIFDGLLLTNESK-RLSSHQCQRHRWL**

**Aq_STK17(XP_003383221.1)**  **FCNVSMCE-YDF-EDEVFDEVSQEAKDFI-EELLQKKPSD-RLTATKCLSHPWM**

**Bf_CON(XP_035661587.1)**  **EQNVRELR-WAF-DAEAFDNLSDECLDFV-DGLLWKDKNS-RYTAQECLEHPWL**

**Bf_DAPK(XP_035665897.1)**  **FANICAVD-FSF-DDEFFGNTSDLAKDFI-RTLLVKHPGR-RASVTDCLSHPWI**

**Bf_TRIO(XP_035662010.1)**  **CTNISKVD-YCF-PEEYFTEVTDLAKQFV-ASFLLADPSQ-RAQAAAGLEHPWV**

**Bf_MYLK(XP_035660911.1)**  **LNNVTEGV-WDF-EDEAFDSISGDAKNFI-EKLLLKDQGS-RLTAAQCMSHPWL**

**Bf_MYLK2(XP_035661607.1)**  **LTNVTLAE-FDF-DDEAFDNISDDAKNFI-EQLLLKDKEN-RNTAVTALQHCWL**

**Bf_OBSCN_A(XP_035660917.1)**  **YTRVKAGR-WAF-DQKIFNHISNEAKDFI-TKMLVVDPKK-RPTASQCLEDVWI**

**Bf_OBSCN_B(GESZ01107032.1+XP_0** **IMKVSKGR-YDI--SRAFNNATPYCRQFV-QDCLVLSPSDRRPSAFYLLNHDWL**

**Bfo_TRIO_like(GHXY01112106.1)**  **--KVIPPQMRAF-HNKNASVLGDSALSFL-TSLLQNEPRR-RPTGQACLNSTWI**

**Bf_STK17(XM_035832943.1)**  **FLNISTLA-YDF-PEELFLDVSADAQDFI-KSLLVKEPED-RATAKECLLHPWL**

**Bm_TRIO_like(GKLW01050522.1)**  **PPQMRAFH-----NTKGNSVLGDNTLVFL-SSLLQNDPRR-RPNGQGCLNSTWI**

**Ce_DAPK(NP_490840.2)**  **FSNITRVR-YHF-SDRYFKNTSKHAKDFI-YRLFVRDVDQ-RATVEECLQHPWI**

**Ce_MYLK(NP_509689.1)**  **YCNVEKGV-WEF--TEEFDTVTEEAKDFV-TKLLVYDQSK-RMLPHECLQHPWI**

**Ce_OBSCN_A(NP_001343714.1)**  **LANVDSCQ---F-DSSPLGNFSYDAGDFV-KKLLTEIPVS-RLTVDEALDHPWI**

**Ce_OBSCN_B(NP_001343714.1)**  **KENVINVK-CD--PNLIPVNASQECLSFA-TWALKKSPVR-RMRTDEALSHKFL**

**Ce_titin(G4SLH0.2)**  **LANVSASD-WDF-DDPSWDDVSDLAKDFI-CRLMIKDKRK-RMSVQDALRHPWI**

**Ce_twitchin(NP_502274.2)**  **LRNVKSCD-WNM-DDSAFSGISEDGKDFI-RKLLLADPNT-RMTIHQALEHPWL**

**Cg_CON_A1(XP_034304307.1)**  **FVNINRCD-YDF-DDEVWQNISSEARDFI-KNLLIPNKSK-RMTIFEALDHPWL**

**Cg_CON_A2(XP_034304307.1)**  **LSNVTRGD-YDF-DDEAFDEISDLAKDFI-NKTIKLNKKK-RLTIDQCLEHPWL**

**Cg_TRIO(XP_034303731.1)**  **CANIVKND-FCF-PEEYFSEISNEAIDLI-KVMLVDHIQS-RPSAQVCLESLWI**

**Cg_MYLK(XP_011441364.2)**  **LVNVTSAK-WDF-SAEEFESISKEAKDFI-SRLLVKDPRK-RMTSRQCLDHIWL**

**Cg_OBSCN_A(XP_034299497.1)**  **LQSTIQYR-WQE-----VDGLSPDAKDFL-SKILIRNQKE-RMTVDGCLSHPWI**

**Cg_OBSCN_B(XP_034299497.1)**  **FANIAYNR-YNA--LSLYENITKEALKFI-FRVLKRVARN-RMTADECLEDKWL**

**Cg_STK17(XP_011422577.1)**  **FLNISQVN-LDF-PENLFKETSPQAQDFI-TRLLVKEPED-RMTAKQCMQHPWL**

**Cg_twitchin(XP_034332726.1)**  **LQNVARCD-WEF-AEEAFSQVSPEAKDFI-RRLLIRRPQE-RMTVHDCLDHAWL**

**Ct_CON(ELT96026.1+AMQN01011565** **FLNITHGV-LDF-DSPLWTNISQCARDWI-KKALVKSPKT-RMTINEALSHPWL**

**Ct_DAPK(ELT88671.1)**  **LANISAVD-FSF-DCEDFANTSLLAKNFI-QGLLLRNPNE-RATVYDCLRHPWI**

**Ct_TRIO(ELU10047.1)**  **CAHIMHND-FSF-PDDFFGAISPEARDFI-SHIVVSDMRQ-RPSAQNCVEHPWM**

**Ct_MYLK(AMQN01000602.1+ELU1638** **LQNVIDGD-YDF-DYPEFEAISSDAKDLV-SKLLVKQNTN-RLSARECLDHSWL**

**Ct_MYLK2(ELT87547.1)**  **FVNVTLTK-WDF-DDDIFDDISDDAKEFI-ETLLMLDPRK-RQSASEALQHKWL**

**Ct_OBSCN(ELU05514.1)**  **LLAVQNGT-WSF-DEESFANISSDLKDFI-SKLLVKDPKK-RVTASAALEHPFI**

**Ct_STK17(ELU16209.1)**  **FLNVSQVN-LDF-PDDIFSDVSSQAIDFM-KQVLVRDPKK-RPSATQCLNHPWF**

**Ct_twitchin(ELT89874.1)**  **MDNVSACD-WDF-DGETFKGISDIGKDFI-RKLIVKNPQK-RMTVHESLDHPWL**

**Dj_DAPK1(IAAB01043597.1)**  **FNSIMRCQ-LNL-EDSCCSMMSDLAKQFL-SSLIVKAPSH-RISSSACLQHPWI**

**Dj_DAPK2(IAAB01051648.1)**  **FQNIVNCR-LDF-EEDVCSDMSEMAKSFI-NSLLVYDPKK-RATASSCLKHPWI**

**Dj_MYLK(IAAB01042781.1)**  **FANILRCN-YSF-KYDEFKEISNDAKSFI-KSLLNKDIKK-RISASECLAHTWL**

**Dj_STK17(IAAB01043480.1)**  **MLNVSKIN-YNF-PEDKFTKSLDEAKDFI-SKLIVKKPNM-RLSATEADRHKWL**

**Dj_twitchin(IAAB01071515.1)**  **LQNVKKCA-YDF-NDDIFKSVSDDAKDFI-RKLLIRTPQK-RMTVYECLDHPWL**

**Dm_MYLK(NP_001260832.1)**  **MSNVTIAK-YDF-EDECFNGISPECLDFI-AKLLAKDLST-RMTAAECMKHKWL**

**Dm_OBSCN_A(XP_034109413.1)**  **LTKIREGR-WDF-KDEIWTHISDDGRDFI-SRLLLYSPEE-RMDVKTALKHPWF**

**Dm_OBSCN_B(XP_034109413.1)**  **KQNISFVR-YRF--ENLFKEVTPEATRFI-MLLFKRHPTK-RPYTEDCLEHRWL**

**Dm_projectin(NP_995598.1)**  **LKNVKACD-WDF-DVESFKYISEEAKDFI-RKLLVRNKEK-RMTAHECLLHPWL**

**Dm_STK17(NP_001162723.1)**  **FLNISQCA-LTF-PDNLFGGVSPVAIDFI-RRALRIKPND-RMNATGCLDHIWL**

**Dm_stretchinMLCK(NP_725510)**  **FSNITRAD-YDY-DDEAFDCVSQEAKDFI-SQLLVHRKED-RLTAQQCLASKWL**

**Ef_CON(GIUK01093179.1)**  **YLNITQGKGANF-STEIWTSVSNYAKNWI-SRLLVKDSKQ-RMTVAEALAHPWL**

**Ef_DAPK(GIUK01016195.1)**  **LSNVSAIK-YDL-DNDYFDGISAHAKDFI-SQLLIREPHK-RATVDDCLNHPWM**

**Ef_DAPK(GIUK01061946.1)**  **LANASLCK-YVF-DEESFCKTSDLAKDFI-QKLLIREPQK-RLSATDCLHHPWI**

**Ef_MYLK2(GIUK01050934.1)**  **LVNVTKAQ-FDF-DDEAFDEISDDAKDFI-SKLLVKDQRK-RMWVEDSLRHPWM**

**Ef_MYLK(GIUK01094627.1)**  **LSNVSKGK-YDF-AYSEFDKVSSDAKDFI-SRLLVTDKKK-RITARQCLDHVWL**

**Ef_MYLK(GIUK01083042.1)**  **LSNVTTGK-FDF-KSAEFQSVSEQAKDFI-RKLLVLDKLK-RMTATMCLDHIWL**

**Ef_OBSCN_A(GIUK01083331.1)**  **LLQVQEGK-WEF-DSEAFADISAEGKDFI-DRLLEVNPKK-RMTATQAIEHPFI**

**Ef_OBSCN_B(GIUK01083331.1)**  **SANIRHVR-YDA--NALYHNVTKYAMKFI-YQTLKRNPRS-RLTVEECLDHRWL**

**Ef_STK17(GIUK01063378.1)**  **YLNISQVN-LDF-PDELFENISKDAMNFM-RSVLIKDPLD-RLTARQCLNHAWF**

**Ef_twitchin(GIUK01013637.1)**  **VNNVKKCD-WNF-DAEGFKGISDQAKDFI-KHLLVRDPTK-RMTIHDALDHSWL**

**Ef_twitchin(GIUK01097374.1)**  **LENVKKCD-WTF-DPETFKGISDIGKDFI-KKLLVKVPQR-RMTIHEALEHPWL**

**Eg_DAPK(KAH9282855.1)**  **FQNILECS-VTF-DRDEFLDVSPEARDFI-TRLLRKNPRK-RDTASQCLRHPWV**

**Eg_MYLK(XP_024351289.1)**  **LSNIMRCS-YTF-DYPEFKDISADAKDFI-KSLLNKNPRK-RNTATQCLAHPWL**

**Eg_STK17(KAH9283880.1)**  **LSNITQMK-IDY-PSHLFEVVSPLALDFI-RSLIKRNPKA-RLSASQCCQHEWL**

**Eg_twitchin(KAH9283145.1)**  **LRNVSRAS-YDF-SDDAFQDVSDNAKDFI-SKLLIKAPEK-RMNVFEALNHPWL**

**Hc_TRIO_like(GHXS01109180.1)**  **PPQMRAFN-----NTRTKSVFGETTLVFL-SSLLQNDPRR-RPNAQGCLNSTWI**

**Hs_CON(NP_003310.4)**  **IENIMNAE-YTF-DEEAFKEISIEAMDFV-DRLLVKERKS-RMTASEALQHPWL**

**Hs_DAPK1(NP_004929.2)**  **LANVSAVN-YEF-EDEYFSNTSALAKDFI-RRLLVKDPKK-RMTIQDSLQHPWI**

**Hs_DAPK2(NP_055141.2)**  **LANITAVS-YDF-DEEFFSQTSELAKDFI-RKLLVKETRK-RLTIQEALRHPWI**

**Hs_DAPK3(NP_001339.1)**  **LTNISAVN-YDF-DEEYFSNTSELAKDFI-RRLLVKDPKR-RMTIAQSLEHSWI**

**Hs_KALRN(NP_001375348.1)**  **CINVCRVD-FSF-PHEYFCGVSNAARDFI-NVILQEDFRR-RPTAATCLQHPWL**

**Hs_MYLK(NP_444253.3)**  **LANVTSAT-WDF-DDEAFDEISDDAKDFI-SNLLKKDMKN-RLDCTQCLQHPWL**

**Hs_MYLK2(NP_149109.1)**  **LNNVLSGN-WYF-DEETFEAVSDEAKDFV-SNLIVKDQRA-RMNAAQCLAHPWL**

**Hs_MYLK3(NP_872299.2)**  **MNFIVNCS-WDF-DADTFEGLSEEAKDFV-SRLLVKEKSC-RMSATQCLKHEWL**

**Hs_MYLK4(NP_001012418.2)**  **LNNILACR-WDL-EDEEFQDISEEAKEFI-SKLLIKEKSW-RISASEALKHPWL**

**Hs_OBSCN_A(NP_001373054.1)**  **LLNVLEGR-VSW-SSPMAAHLSEDAKDFI-KATLQRAPQA-RPSAAQCLSHPWF**

**Hs_OBSCN_B(NP_001373054.1)**  **QRGLRKGL-VRL--SRCYAGLSGGAVAFL-RSTLCAQPWG-RPCASSCLQCPWL**

**Hs_SPEG_A(NP_005867.3)**  **LMNIRNYN-VAF-EETTFLSLSREARGFL-IKVLVQD-RL-RPTAEETLEHPWF**

**Hs_SPEG_B(NP_005867.3)**  **EARIVGGR-FDA--FQLYPNTSQSATLFL-RKVLSVHPWS-RPSLQDCLAHPWL**

**Hs_ST17A(NP_004751.2)**  **FLNISQMN-LSY-SEEEFDVLSESAVDFI-RTLLVKKPED-RATAEECLKHPWL**

**Hs_ST17B(NP_004217.1)**  **YLNISQVN-VDY-SEETFSSVSQLATDFI-QSLLVKNPEK-RPTAEICLSHSWL**

**Hst_TRIO(GKDX01093008.1)**  **RDNVLTVR-YSF-PAKLFRDVSESAKDLV-AKMLVKDMSN-RISSLQCLGHPWL**

**Hst_TRIO_like_A(GKDX01095742.1** **TERIKEAR-YDF-YDSQFEQISPEAKEFI-SALLQKSTEY-RMTASQALQHSWI**

**Hst_TRIO_like_B(GKDX01095742.1** **RSNIANIH-LVT-ESEDFWSLSSDCKDFLTQRVLIKNPRR-RLTANQCQRHRWL**

**Hs_TRIO(NP_009049.2)**  **CLNICRLD-FSF-PDDYFKGVSQKAKEFV-CFLLQEDPAK-RPSAALALQEQWL**

**Hv_CON_A(XP_047137784.1)**  **LTNVTNGD-WDF-DDPVFEDISDEAKDFI-SDLLVKDPKK-RATVKDCLDHPWF**

**Hv_CON_B(XP_047137784.1)**  **KDAITALD-FRF-EPREFSTVTEEAKTFI-KRILIRAPEK-RPSAQQCLQDPWF**

**Hv_DAPK1(XP_012558089.1)**  **YEAIVKVD-YDF-EDDSFEQISCHAKDFI-SGLLVKQPAD-RLTAEECLKHDWL**

**Hv_DAPK2(XP_047139830.1)**  **LEAIGRVD-YEF-DDESFGHISNYALEFI-SNLLIKQPKK-RLTAEGCLKHDWL**

**Hv_TRIO(XP_047137671.1)**  **CENITQLR-YKF-PGDFFGDISDEAKDFI-EDLLVAEQSL-RPNAKKCLESTWL**

**Ml_TRIO_like(GFAT01019191.1)**  **PPQMRAFH-----NTPGNSVLGDNTLVFL-SSLLQNDPRR-RLNGQGCLNSTWI**

**Nv_CON_A1(XP_048579265.1)**  **IQNVSGAE-WDF-EDESFEVVSDMSKKFI-EELLIRDPKK-RNDIYKCLGHEWL**

**Nv_CON_A2(XP_048579265.1)**  **LANVQTAE-WDF-DDPVFDDISDEAKDFI-EKLLVLKATA-RISVAKCMEHPWL**

**Nv_CON_B(XP_048579265.1)**  **EEAVKQAR-CTF-VGRAFRSLTDHSKGFI-EKLLYKIPSK-RLEVAGALTHEWL**

**Nv_DAPK(XP_032239611.2)**  **FSNISHVD-YEF-DDEYFKEISQPAKDFI-EGLLIKKPSD-RMTAKECLAHEWL**

**Nv_TRIO(XP_032241348.2)**  **CERIRKAN-FSF-PNKHFSAISSQAKDFI-SSLLIADITK-RADCQDCFKSDWI**

**Nv_STK17(XP_001629087.2)**  **CYNVSLCA-IDF-PESHFDNISYTAQDFI-RTVLQRCPGD-RPTVEDCLSHPWL**

**Om_TRIO_like_A(KAI6652169.1)**  **AYNILTCN-YSL-QESVFQDISQEAQDFI-QQLLVLDSQV-RLTANGCLSHPWV**

**Om_TRIO_like_B(KAI6652169.1)**  **HEPVIREK-CQL----LSSHLSIFGQQFV-EQALVINPTR-RMTAEIAATHPWF**

**Om_STK17(KAI6649438.1)**  **LSNVSQAK-YDF-DDEVFEGVSQNAKEFI-RNLLVLNPKR-RPDAEQCLRHQWL**

**Os_CON(XP_036371388.1)**  **FAYINNID-YDF-DDEAWENVSEIAKDFI-ANLLVKDKRE-RMTIDEALNHPWL**

**Os_DAPK(XP_029643431.1)**  **YQNITSVD-YDY--EDYFETTSKLAKDFI-DKLLVKNPRK-RASATDCMSHPWI**

**Os_TRIO(XP_029657616.1)**  **CSNIVRND-YSF-PDEYFAGISPEAKDLA-RSMLMEDMNK-RPLAQHCLESPWI**

**Os_MYLK(XP_029655504.1)**  **LSNVTQAV-YDF-DDETFDEISDAAKDFI-SKLLVKKKEN-RMTIHMCLQHEWL**

**Os_OBSCN_A(XP_036361934.1)**  **LNKIENGE-WHF-DEEAFKHISDEAKDFI-TSLLKKEPLF-RLEIDECLSHPWI**

**Os_OBSCN_B(XP_036361934.1)**  **LANIAYNR-FDG--SNMYENISKEALKFI-SKTIKRIPGN-RMTVDDCLEHKWL**

**Os_STK17(XP_029636789.1)**  **YLNISQVN-LDF-PDYLFESISYGAIDFM-KNLLIKDPRC-RMDAAQCLTHAWI**

**Os_twitchin(XP_036360273.1)**  **LQNVKRCD-WDF-DEEAFSNVSAEAKDFI-KRLLLRNPAK-RLTVHEAIDHAWL**

**Pc_DAPK(XP_045596115.1)**  **FNNVTAVD-YTF-DVEYFCGTSDLAKDFI-CRLLVKDSRK-RLTAEESLAHPWI**

**Pc_MYLK(XP_045598454.1)**  **MTNVTRNK-YDF-EDEAFLSVSEDAKDFI-QKLLVLDKSL-RLTPAQCLRHDWL**

**Pc_OBSCN(XP_045586627.1)**  **LTKVQKGE-LNF-DMEAFTHISDEAKDFI-AKLLVFKADM-RMDVKTALKHPWM**

**Pc_projectin(XP_045599281.1+BA** **LKNVKACD-WDF-DEEAFSNVSNEAKDFI-RRLLIKNKEK-RMTAHECLMHAWL**

**Pc_STK17(XP_045598575.1)**  **FLNISQGQ-VDF-PKELFCDVSDQAIDFI-TRLLVVNPSC-RLTVDEAIQHQWL**

**Pc_stretchinMLCK(XP_045616175.** **FANITLAE-FDF-DDDAFSAITDDAKDFI-TSLLIKEKEE-RLTAEECFNHPWL**

**Sk_CON(XP_006823011.1)**  **LRNVSKSE-WDF-DEEAFDDISDEALDFI-EKLLVKEKPQ-RMSVQEAIRHPWL**

**Sk_DAPK1(XP_006823401.1)**  **YENIVAVD-YEF-DDQYFSKTSEFAKDFI-EKLFVKDARK-RATVTECLNHPWI**

**Sk_TRIO(XP_006819276.1)**  **CVNIAKID-YSF-PQEYFSDISQAATNFI-SNLLKEDPEE-RLSAKACLENAWI**

**Sk_MYLK(XP_006823003.1)**  **ICNVTTAE-WDF-EDESFDEISDAAKDFI-EKLLVLDPRE-RNLAKDCREHDWL**

**Sk_OBSCN_A(XP_006823009.1)**  **LVNVKNGK-WSF-EDEVFNKVTEEAKDFI-SRLLVLDPSI-RMTTEECLDHPWL**

**Sk_OBSCN_B(XP_006823009.1)**  **MAHILTTH-CDL--SLLYQQVSQHARDFI-NKTLQRHASD-RLNVLESLQHPWL**

**Sk_STK17(XP_002741442.1)**  **FLNISQCK-ADF-SSDLWKDISPLAVDFI-KRLLVVQPTK-RYKMKDCLEHPWI**

**Sk_twitchin(XP_006825350.1)**  **-GRVKRCD-WTF-DSDSFKGISDGAKDFI-KKLLVADKHE-RMTAVDALEHQWL**

**Sp_CON(XP_030830392.1)**  **LKNVATGE-WDF-DEEAFADISDEGLDWI-ERILIKGKEG-RMTIQEALDHPWL**

**Sp_DAPK(XP_030832558.1)**  **YENVTAIN-YSFEDDDFFSSTSELAKDFI-DHLLLKDPRK-RATVDQCLSHPWI**

**Sp_TRIO(XP_011680751.1)**  **NMNIIRVD-YSF-PEEYFKNVSQNAKHFI-TGLLHADSSE-RKTTSECLMNHWF**

**Sp_MYLK_A1(XP_030830851.1)**  **LNNVTMGE-WDF-EDEAFDGISNCAKDFI-SDLLVKDQRD-RTSVDDSFKHPWL**

**Sp_MYLK_A2(XP_030830851.1)**  **LNNVTLAE-WDF-EDEAFDAISEDAKTFI-EGLLIQKKEE-RMTAAECLQHHWL**

**Sp_OBSCN_A(XP_030830075.1)**  **LLNVRGGQ-WDF-DDEVWDDISDEAQDFI-WLLFEMNADK-RPGLKEVSEHPWL**

**Sp_OBSCN_B(XP_030830075.1)**  **IYNITRMK-YNI--SSLYPNASPEAKAFL-QAIFKEKPID-RPTVADCLEHSWV**

**Sp_STK17(XP_030846519.1)**  **FLNISQLN-LDF-AEEDFSCHSPEARDFI-QSLCVIDAEK-RLSAKECLDHPWI**

**Sp_twitchin(XP_030829654.1)**  **---IKSCK-WSF-DKSAFREISPEAQDFI-SRLLIKDKSE-RMAAHEALDHPWL**

**Ta_CON_A1(GHJI01005353.1)**  **LQNVTNAE-WDF-DDEIFDELSENSKNFM-EGLIQKDPKS-RFTIEQALNHSWL**

**Ta_CON_A2(GHJI01005353.1)**  **LSNVTACN-WDF-DDDIFDDISAEAKSFI-SSLLRRDPKQ-RATVNEALDHAWL**

**Ta_TRIO(GHJI01000868.1)**  **CKMIVSGS-Y---DSTFLKDMSDDCRNFI-KHCLAIDPCK-RKSASWCLTSGWL**
